# Supplementary material for: Remarkable catalytic activity of dinitrogen-bridged dimolybdenum complexes bearing NHC-based PCP-pincer ligands toward nitrogen fixation
Source: Nat Commun. 2017 Apr 4;8:14874. doi: 10.1038/ncomms14874 (PMC5382288; doi:10.1038/ncomms14874)
Supplement: Supplementary Information — Supplementary figures, supplementary tables, supplementary methods and supplementary references. [file ncomms14874-s1.pdf]

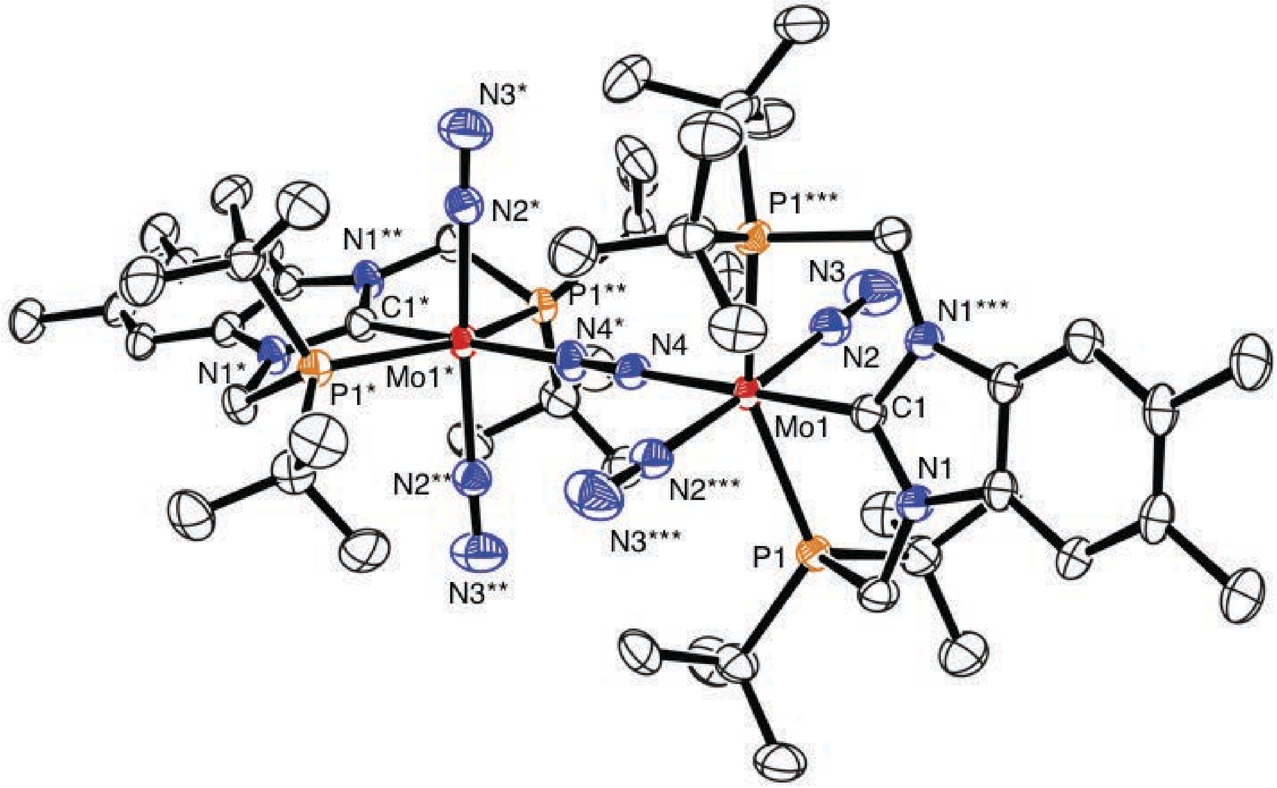

Supplementary Figure 1 | ORTEP drawing of 1c. Thermal ellipsoids are shown at the 50% probability level. Hydrogen atoms are omitted for clarity. Asterisks (*) denote atoms related by the symmetrical operation –x, –y, +z. Asterisks (**) denote atoms related by the symmetrical operation –x, +y, –z+1. Asterisks (***) denote atoms related by the symmetrical operation +x, –y, –z+1. Selected bond lengths and angles are included in Supplementary Table 5.

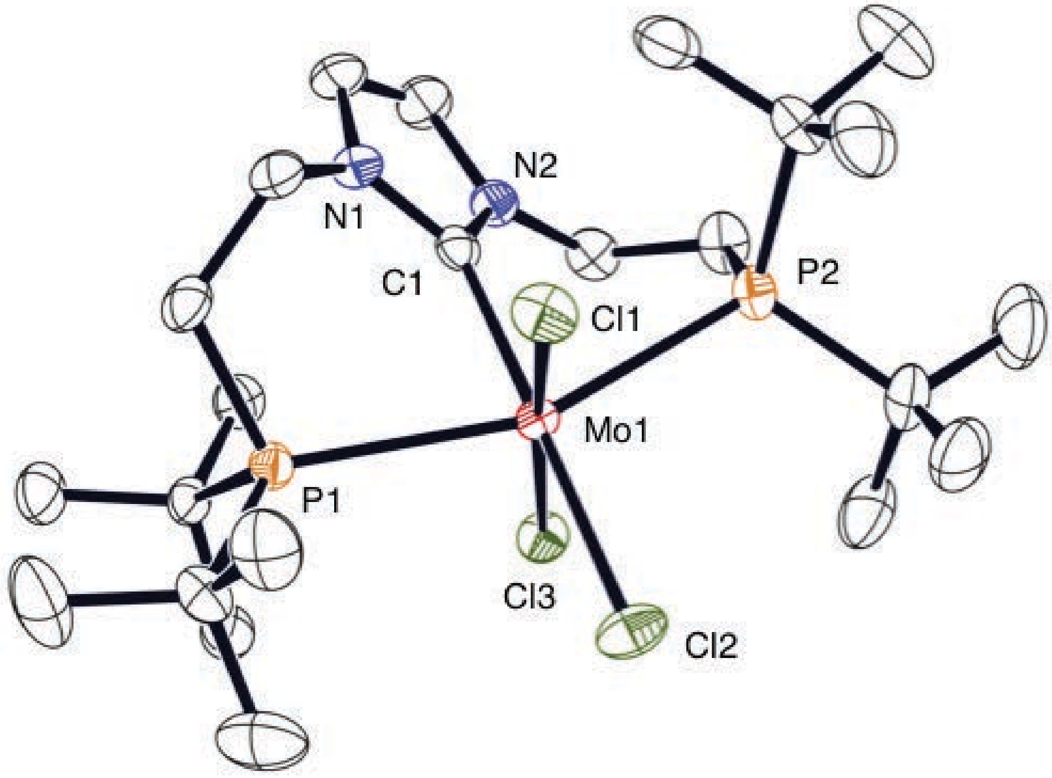

Supplementary Figure 2 | ORTEP drawing of 3b. Thermal ellipsoids are shown at the 50% probability level. Hydrogen atoms and solvated molecules are omitted for clarity. Selected bond lengths and angles are included in Supplementary Table 6.

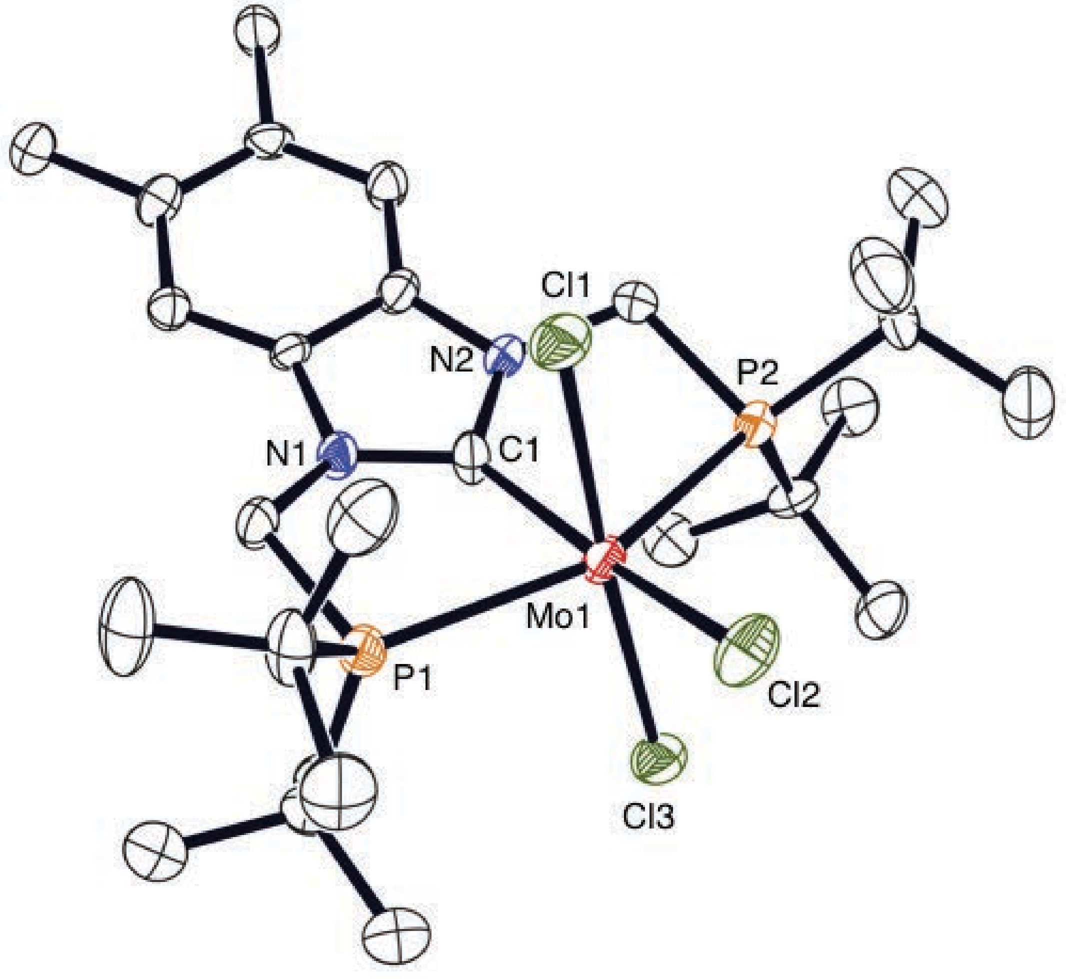

Supplementary Figure 3 | An ORTEP drawing of 3c. Thermal ellipsoids are shown at the 50% probability level. Only one of the two crystallographically independent molecules is shown, while hydrogen atoms and solvated molecules are omitted for clarity. Selected bond lengths and angles are included in Supplementary Table 7.

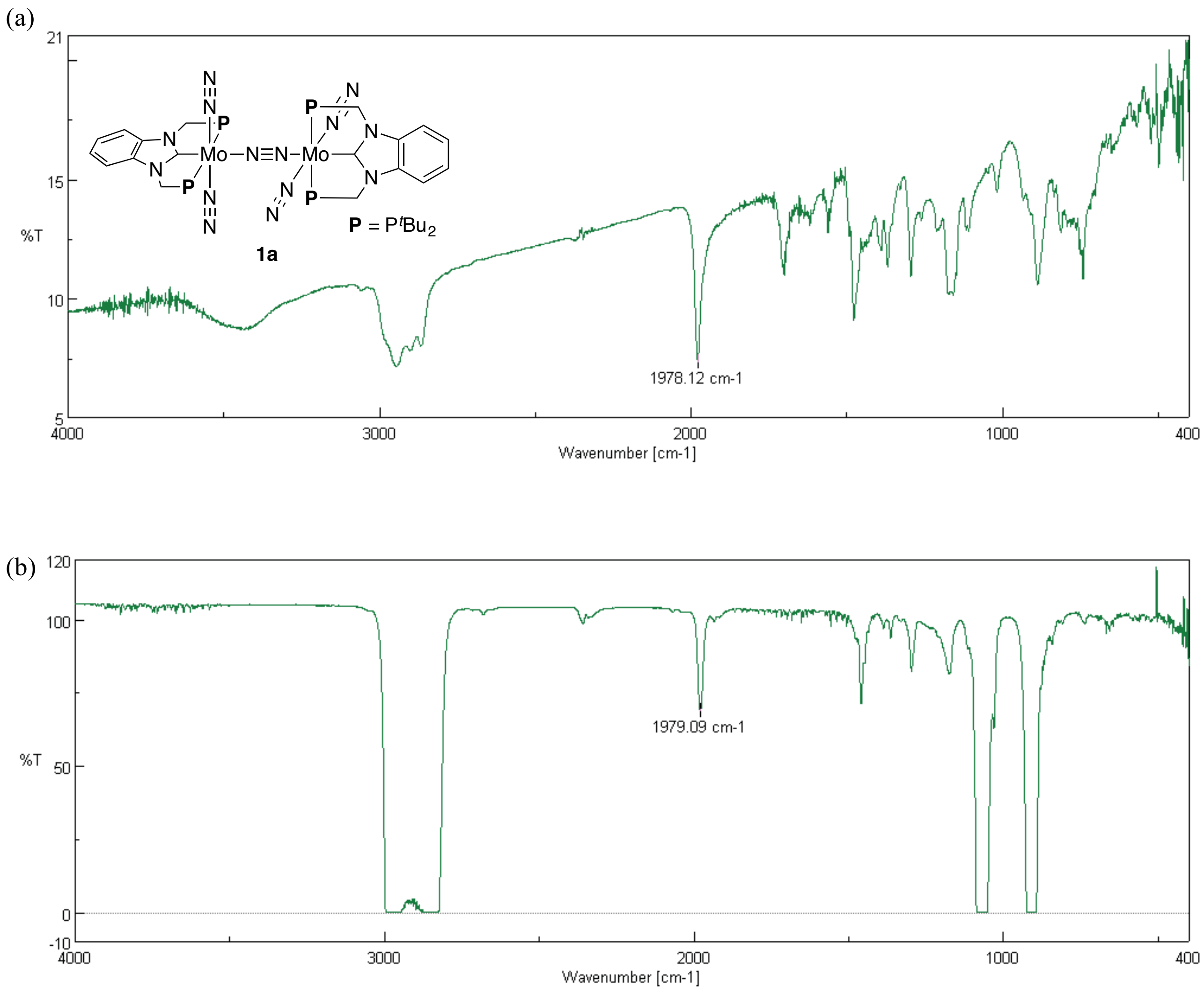

Supplementary Figure 4 | IR spectra of 1a. (a) IR spectrum of **1a** in KBr. (b) IR spectrum of **1a** in THF.

(a)

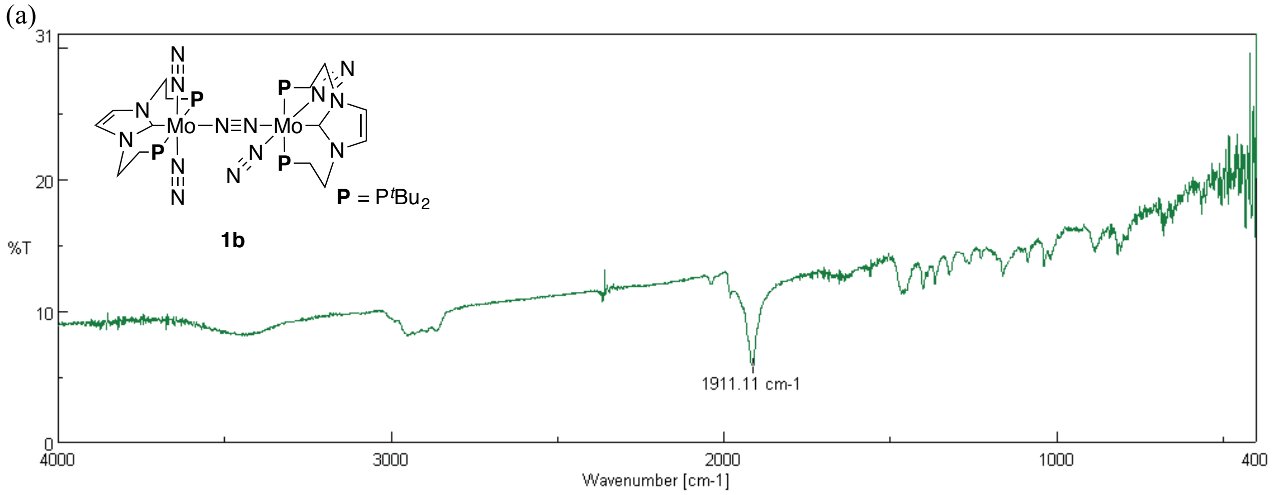

(b)

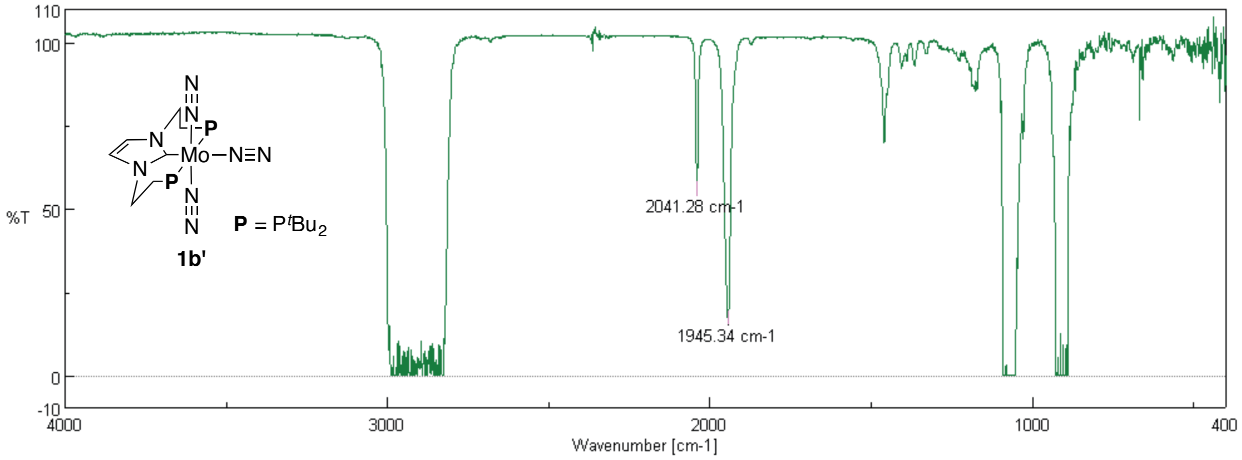

Supplementary Figure 5 | IR spectra of 1b. (a) IR spectrum of **1b** in KBr. (b) IR spectrum of **1b′** in THF. This spectrum was measured after confirming that almost all of **1b** was converted into **1b′** in THF by $^{31}P\{^1H\}$ NMR.

(a)

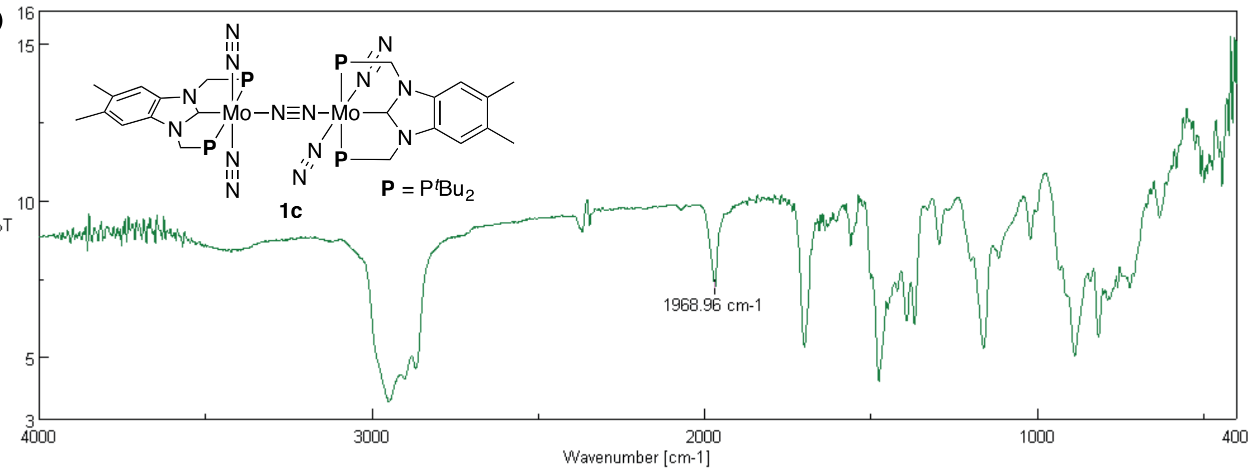

(b)

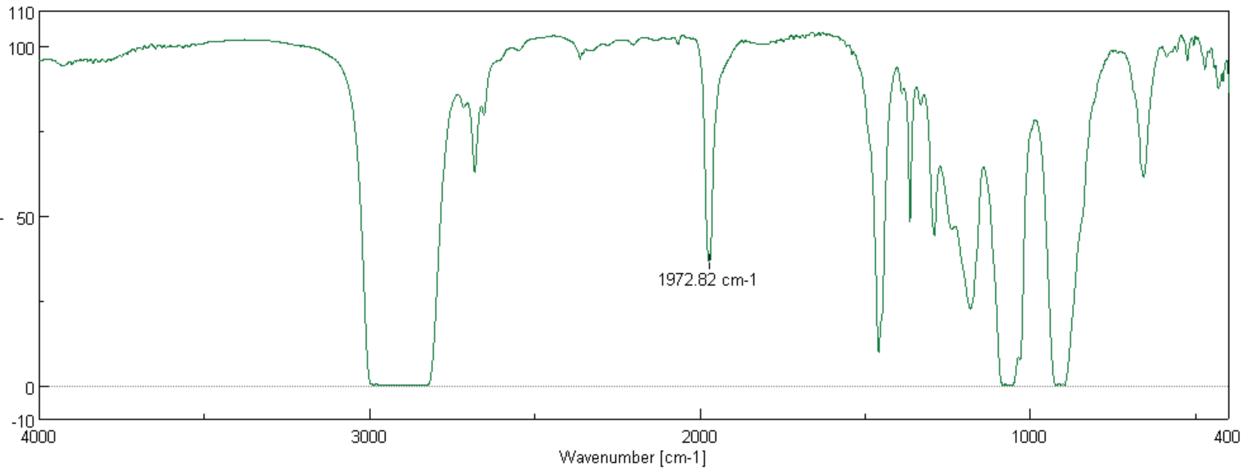

Supplementary Figure 6 | IR spectra of 1c. (a) IR spectrum of **1c** in KBr. (b) IR spectrum of **1c** in THF.

(a)

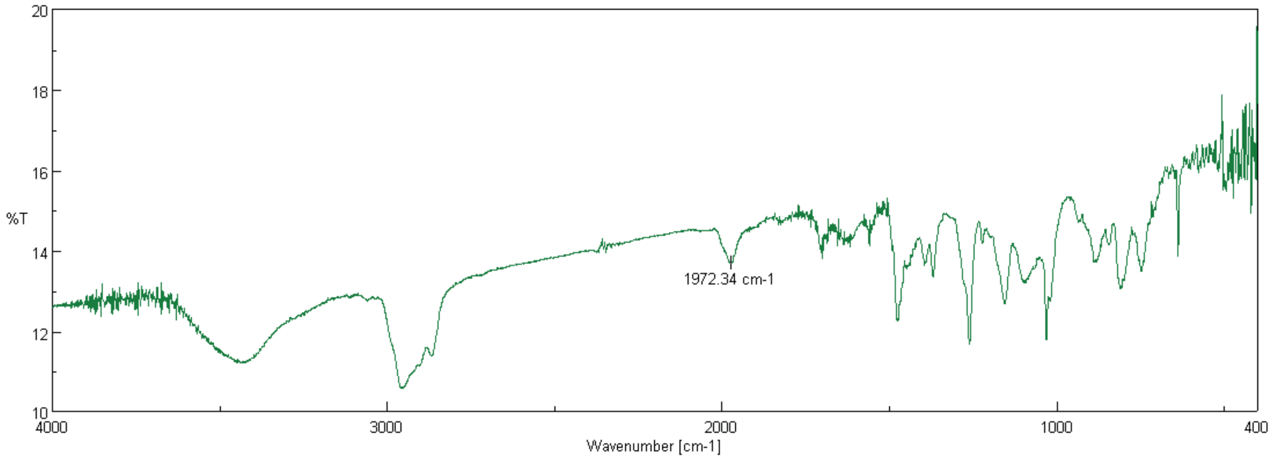

(b)

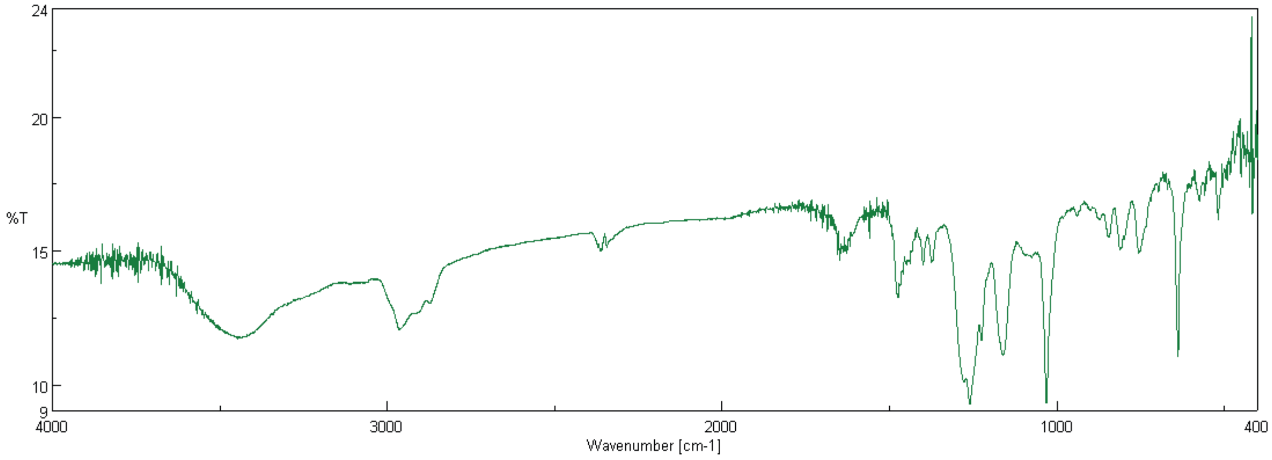

Supplementary Figure 7 | IR spectra of reaction mixture (a) obtained from the supernatant solution of the reaction of 1a and 2 equivalents of [LutH]OTf in toluene (b) obtained from the precipitate of the reaction of 1a and 2 equivalents of [LutH]OTf in toluene.

(a)

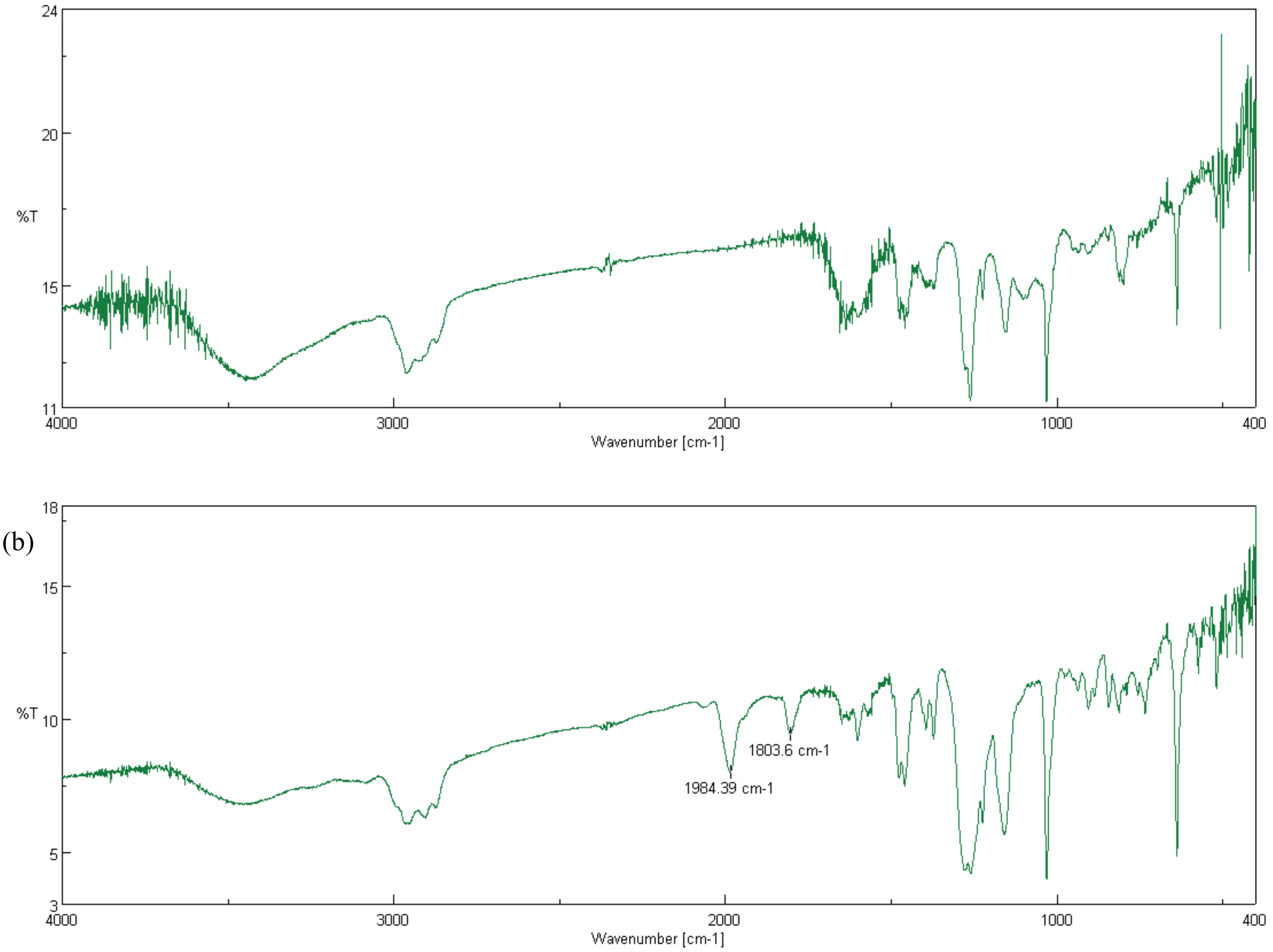

Supplementary Figure 8 | IR spectra of reaction mixture (a) obtained from the supernatant solution of the reaction of 2 and 2 equivalents of [LutH]OTf in toluene. (b) obtained from the precipitate of the reaction of 2 and 2 equivalents of [LutH]OTf in toluene.

(a)

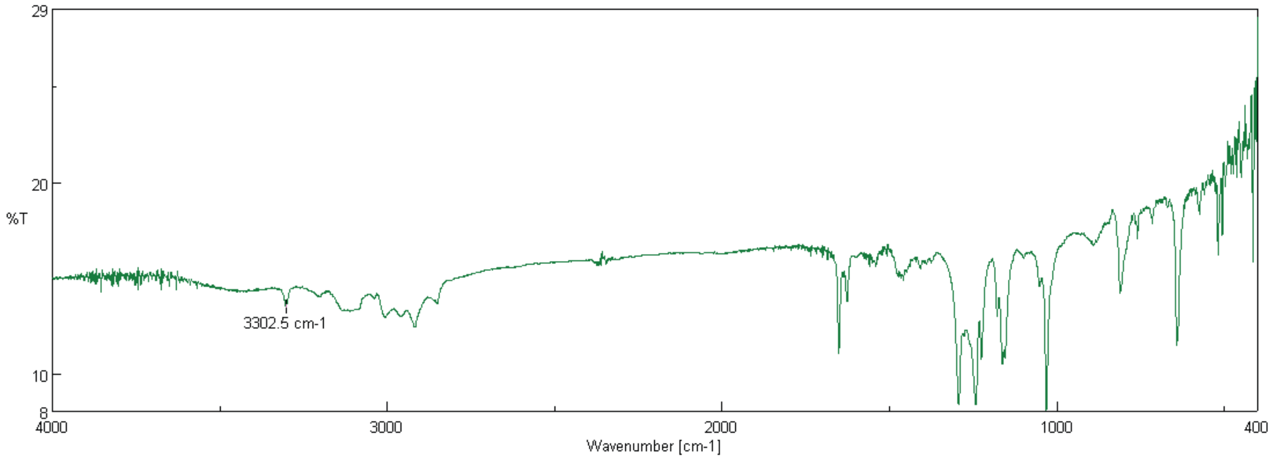

(b)

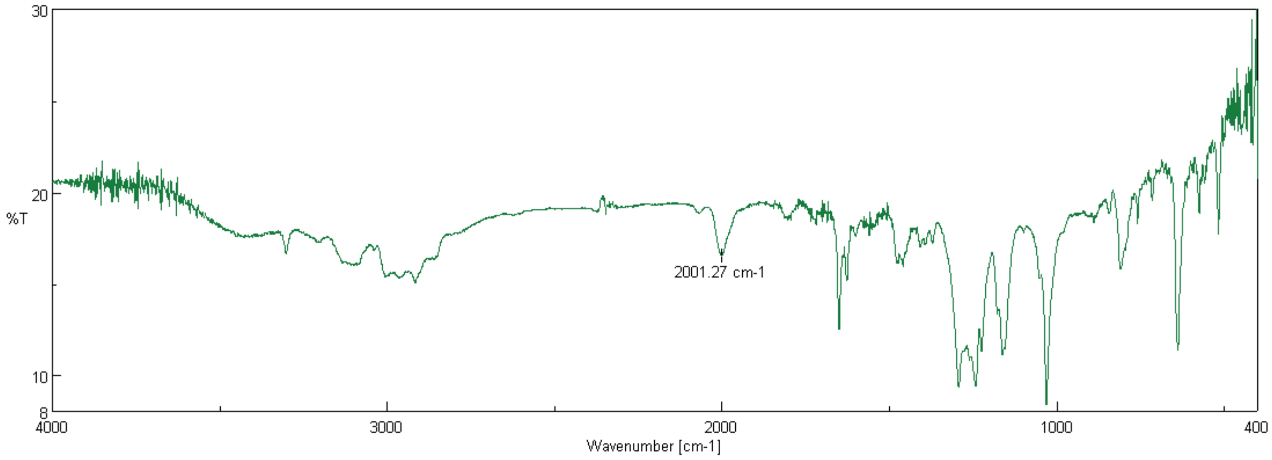

Supplementary Figure 9 | IR spectra of reaction mixture (a) obtained from the supernatant solution of the reaction of 2 and 5 equivalents of [LutH]OTf in toluene. (b) obtained from the precipitate of the reaction of 2 and 5 equivalents of [LutH]OTf in toluene.

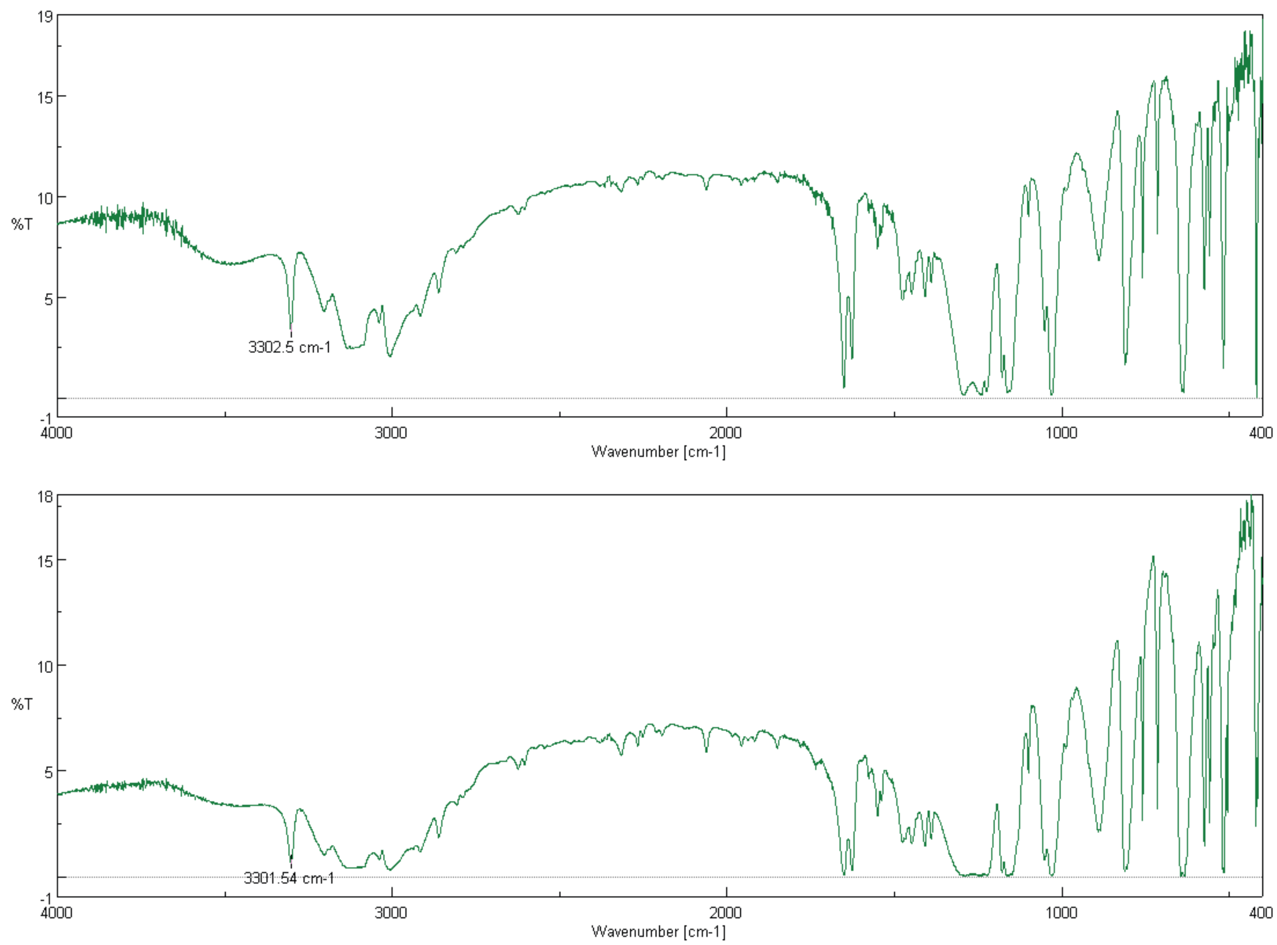

Supplementary Figure 10 | IR spectra of reaction mixture (a) obtained from the supernatant solution of the reaction of 1a and 96 equivalents of [LutH]OTf in toluene. (b) obtained from the precipitate of the reaction of 1a and 96 equivalents of [LutH]OTf in toluene.

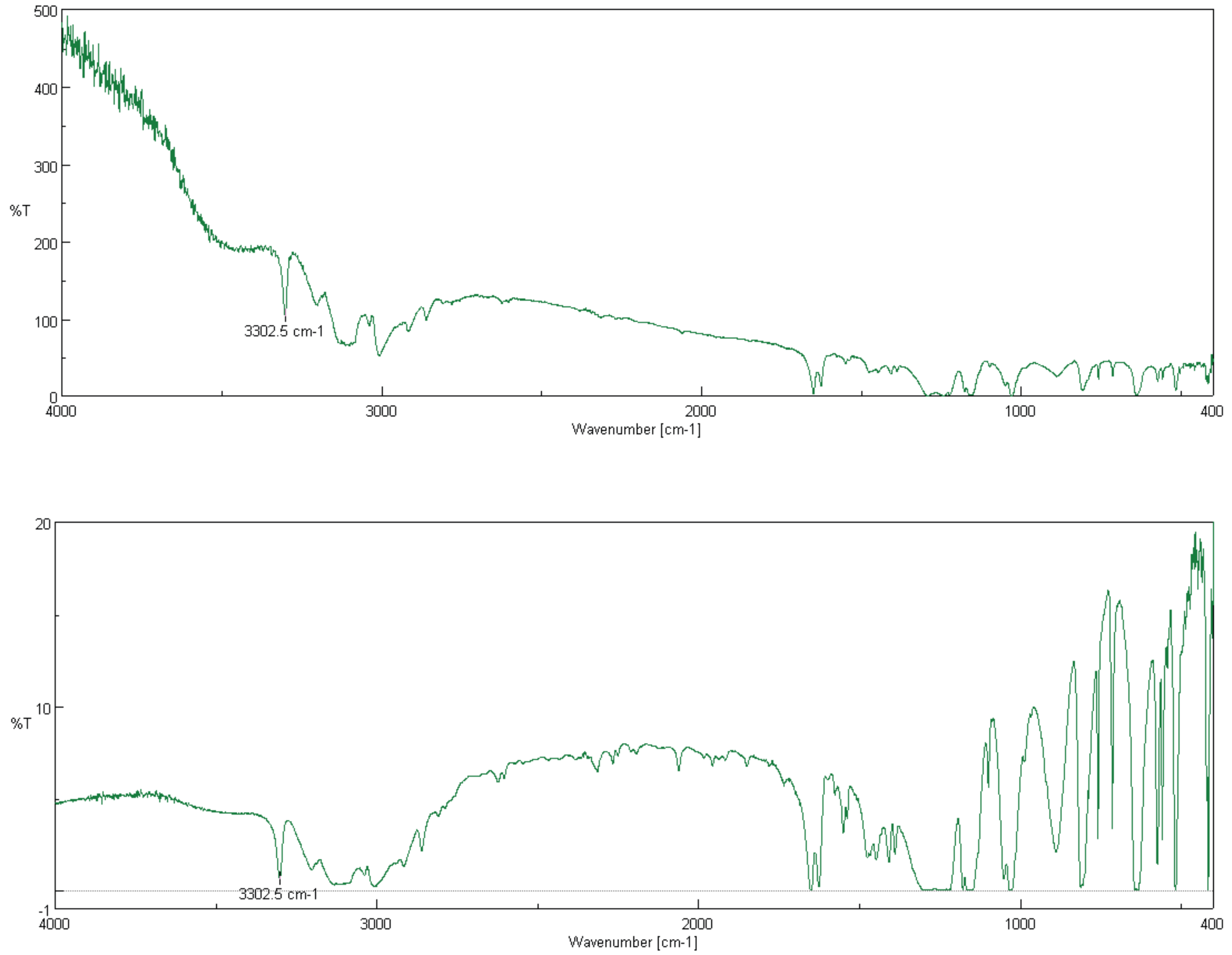

Supplementary Figure 11 | IR spectra of reaction mixture (a) obtained from the supernatant solution of reaction of 2 and 96 equivalents of [LutH]OTf in toluene. (b) obtained from the precipitate of the reaction of 2 and 96 equivalents of [LutH]OTf in toluene.

(a)

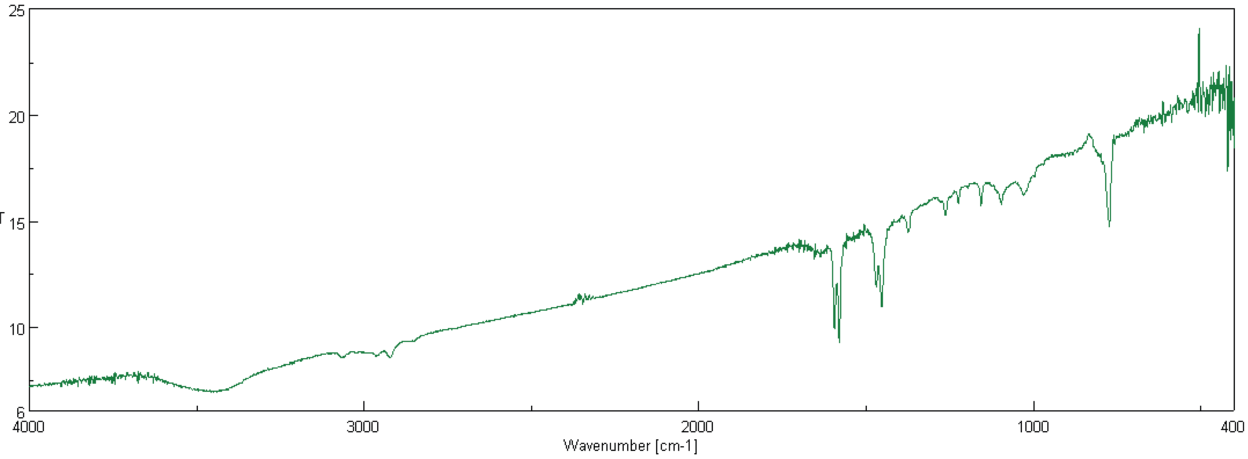

(b)

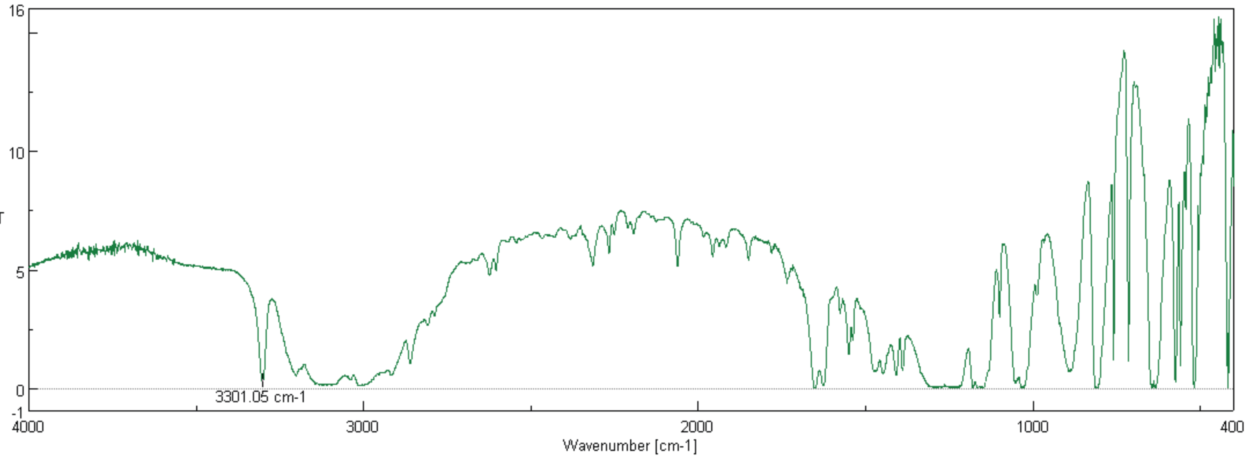

Supplementary Figure 12 | IR spectra of (a) 2,6-lutidine (b) 2,6-lutidinium trifluoromethanesulfonate ([LutH]OTf).

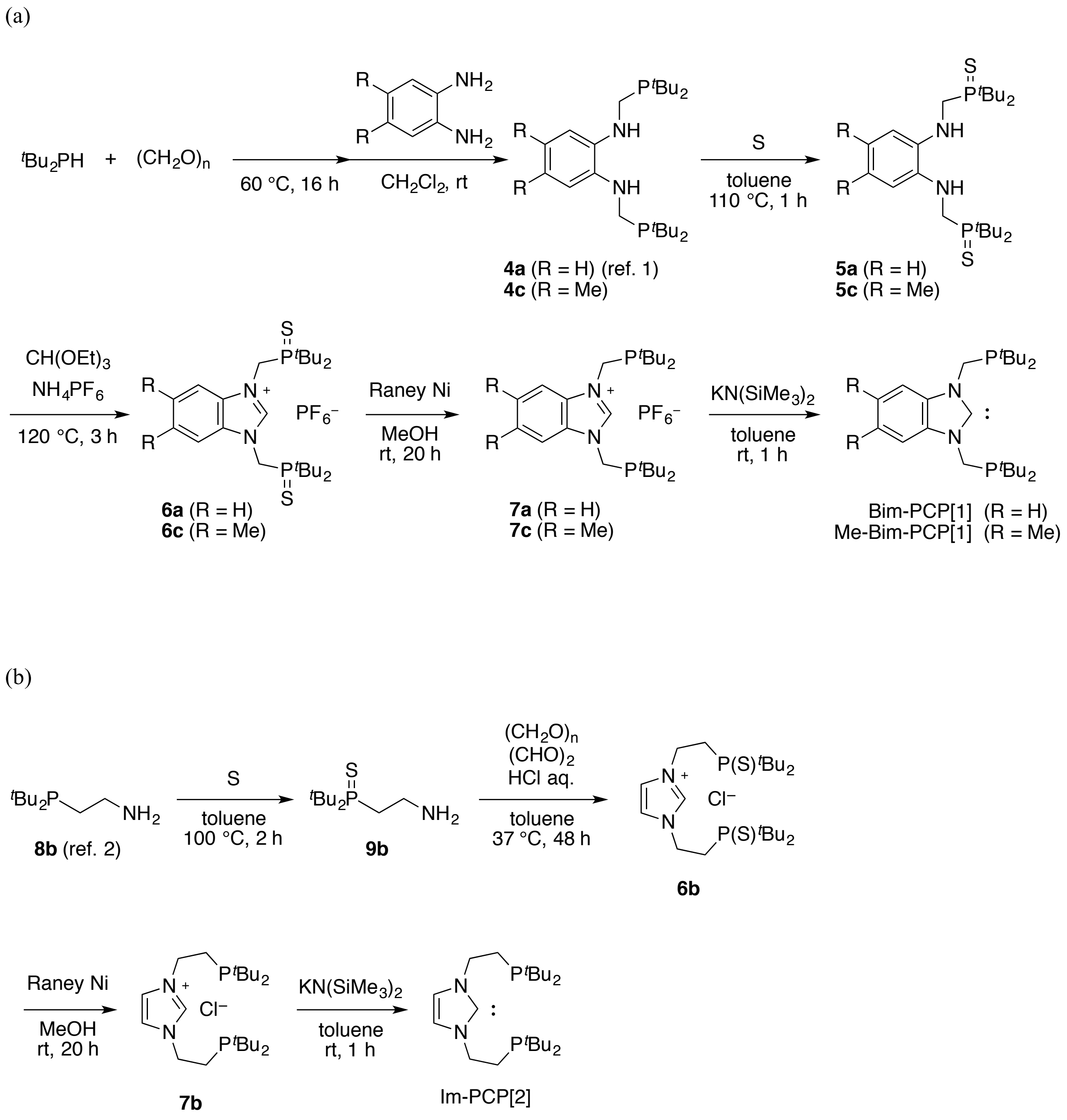

Supplementary Figure 13 | Entire scheme for preparation of Bim-PCP[1], Im-PCP[2], Me-Bim-PCP[1] (a) Scheme for preparation of Bim-PCP[1] and Me-Bim-PCP[1]. (b) Scheme for preparation of Im-PCP[2].

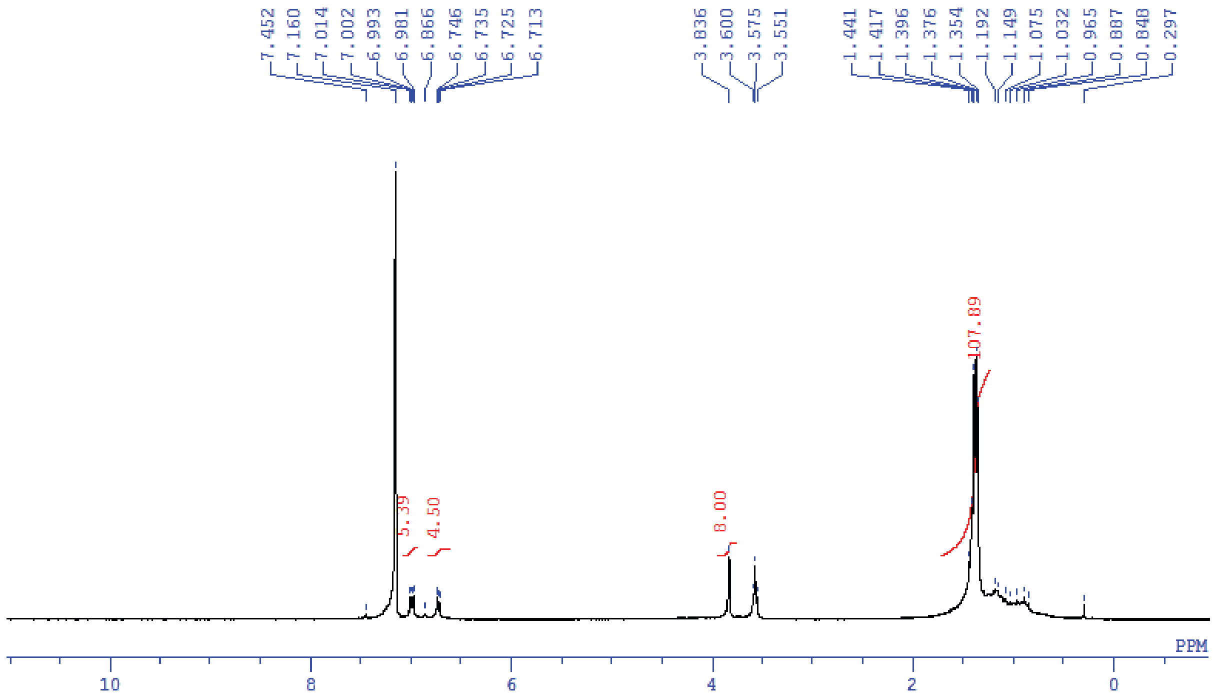

Supplementary Figure 14 | ^{1}H NMR (C_6D_6) spectrum of 1a.

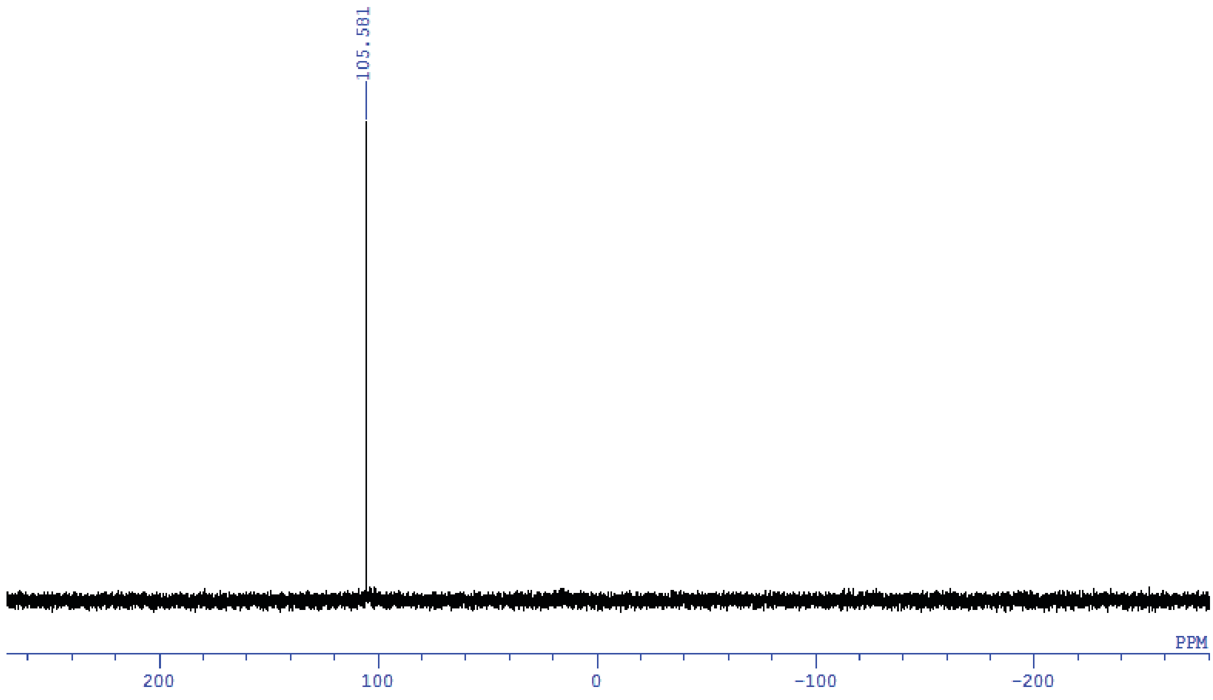

Supplementary Figure 15 | ^{31}P{^{1}H} NMR spectrum of 1a.

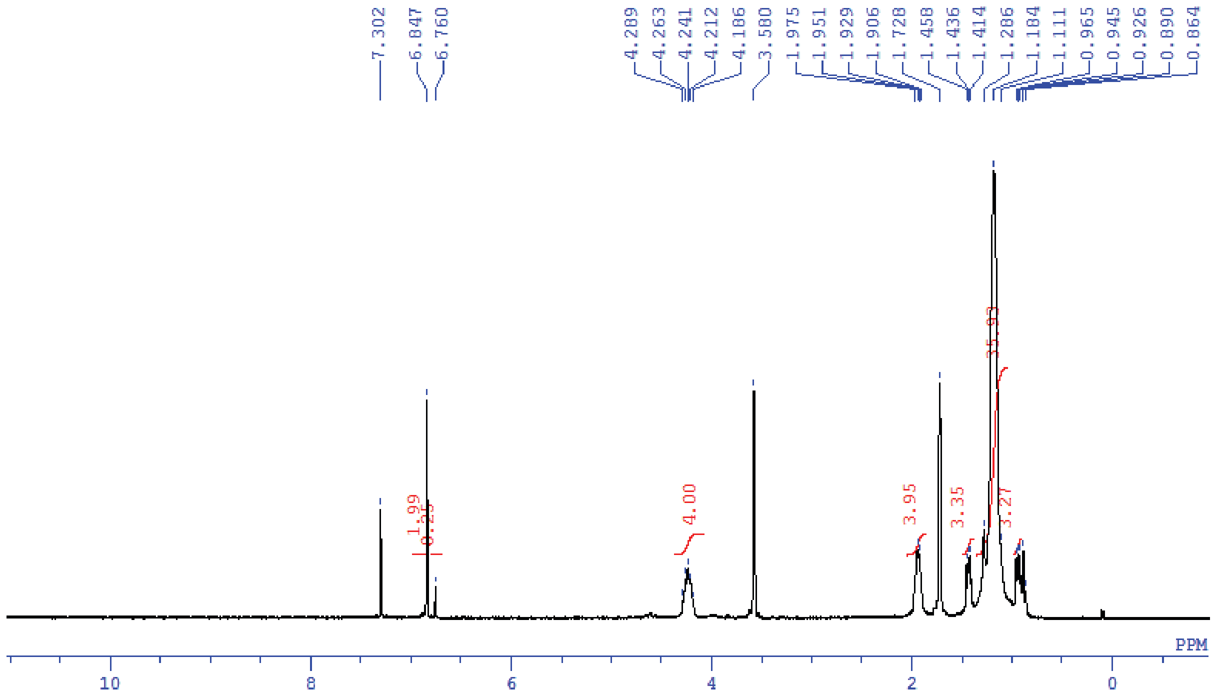

Supplementary Figure 16 | ^{1}H NMR (THF-d_8) spectrum of 1b.

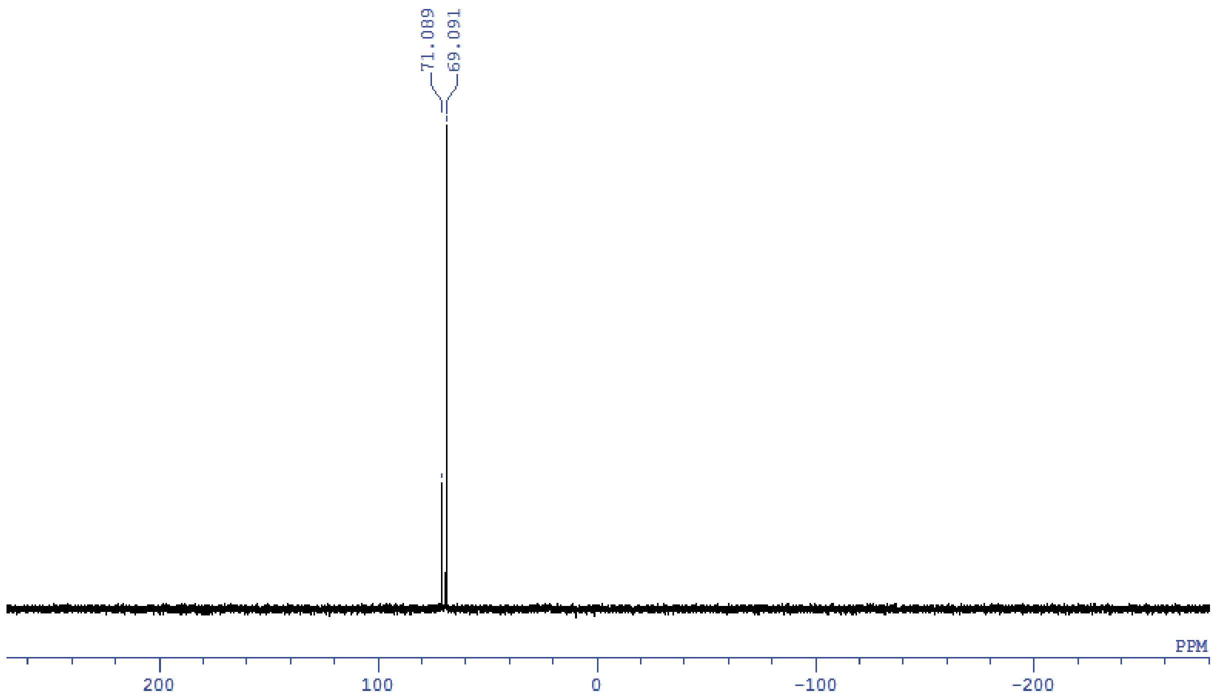

Supplementary Figure 17 | ^{31}P{^{1}H} NMR (THF-d_8) spectrum of 1b.

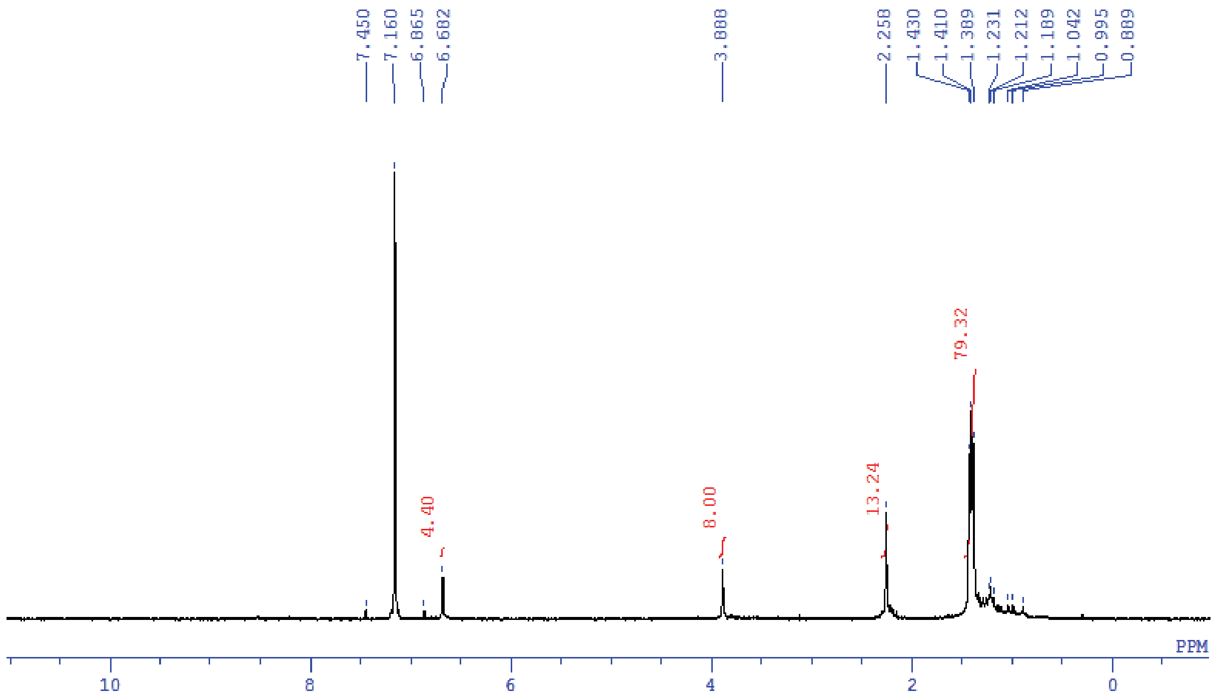

Supplementary Figure 18 | ^{1}H NMR (C_6D_6) spectrum of 1c.

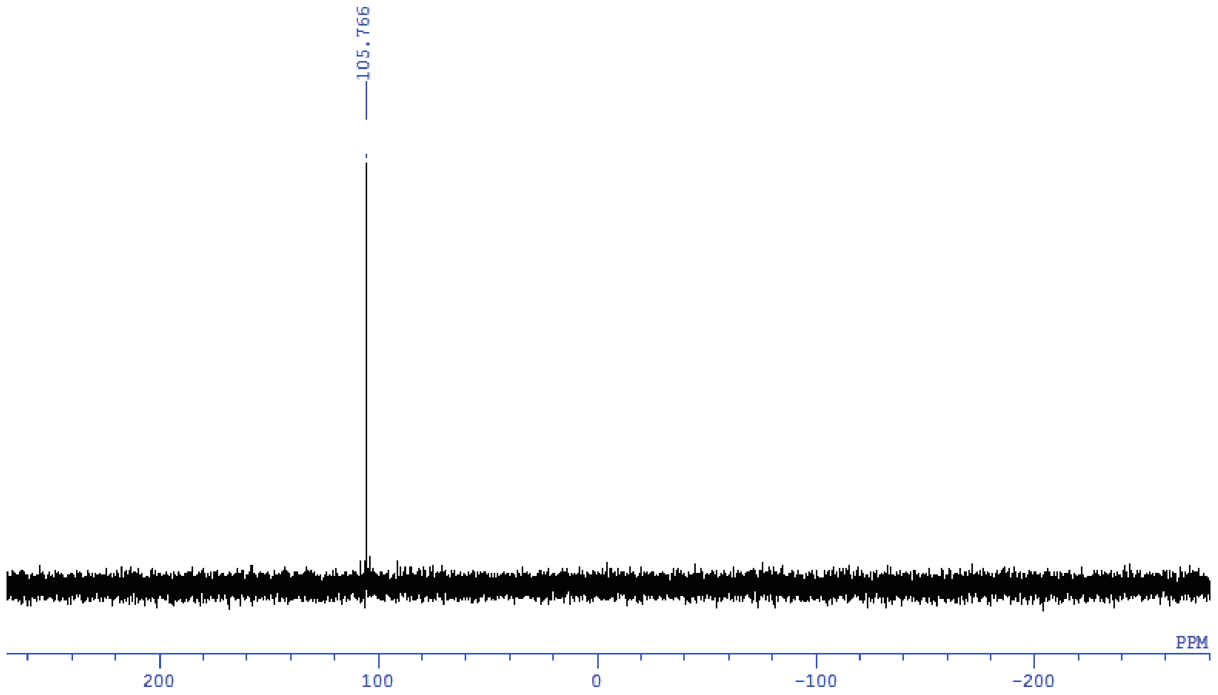

Supplementary Figure 19 | $^{31}P\{^{1}H\}$ NMR (C_6D_6) spectrum of 1c.

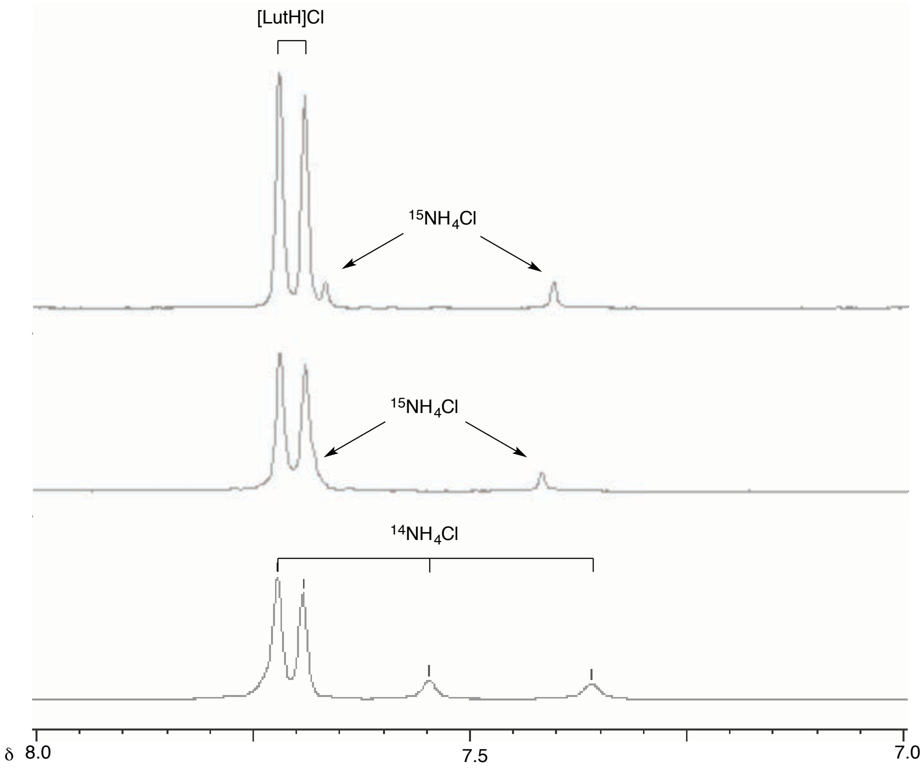

Supplementary Figure 20 | ^{1}H NMR (DMSO-d_6) spectra of (a) $^{15}NH_4Cl$ obtained from the reaction of 1a with [LutH]OTf and CrCp*$_2$ under $^{15}N_2$, (b) a mixture of authentic $^{15}NH_4Cl$ and [LutH]Cl, (c) a mixture of authentic $^{14}NH_4Cl$ and [LutH]Cl.

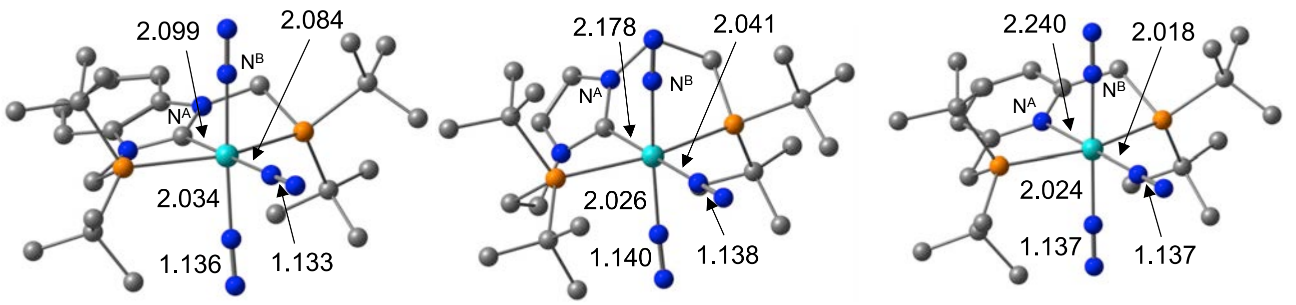

N^A–C–Mo–N^B = 69.6° | N^A–C–Mo–N^B = 43.8° | C–N^A–Mo–N^B = 67.7°

1a′ | **1b′** | **2′**

Supplementary Figure 21 | Optimized structures of mononuclear molybdenum-dinitrogen complexes 1a′, 1b′, and 2′. Hydrogen atoms are omitted for clarity.

Supplementary Table 1 | X-ray crystallographic data for 1a, 1b, 1c

	**1a**	**1b**	**1c**
chemical formula	$C_{50}H_{88}Mo_2N_{14}P_4$	$C_{46}H_{92}Mo_2N_{14}P_4$	$C_{54}H_{96}Mo_2N_{14}P_4$
CCDC number	1482254	1482255	1482256
formula weight	1201.11	1157.10	1257.22
dimensions of crystals	0.20 × 0.12 × 0.03	0.25 × 0.15 × 0.02	0.20 × 0.05 × 0.02
crystal color, habit	red, platelet	brown, platelet	red, block
crystal system	monoclinic	orthorhombic	orthorhombic
space group	*C*2	*Ccce*	*I*222
a, Å	23.2844(9)	22.3373(8)	10.4281(4)
b, Å	11.4601(4)	37.4463(14)	12.6656(4)
c, Å	11.8307(4)	22.4667(9)	24.5490(8)
α, deg	90	90	90
β, deg	111.6786(9)	90	90
γ, deg	90	90	90
V, Å^3	2933.63(18)	18792.3(12)	3242.38(18)
Z	2	16	2
ρ_{calcd}, g cm^{-3}	1.360	1.227	1.288
F(000)	1260.00	7320.00	1324.00
μ, cm^{-1}	5.818	5.421	5.295
trans. factors range	0.731–0.983	0.592–0.989	0.814–0.989
no. reflections measured	14444	71943	15882
no. unique reflections	6126 (R_{int} = 0.0409)	8580 (R_{int} = 0.1098)	3725 (R_{int} = 0.0318)
no. parameters refined	361	466	194
$R1$ ($I > 2\,\sigma(I)$)[a]	0.0323	0.0475	0.0217
$wR2$ (all data)[b]	0.0639	0.0953	0.0576
GOF (all data)[c]	1.021	1.011	1.039
Flack parameters	0.01(2)	-	0.14(2)
max diff peak / hole, e Å^{-3}	1.47/–1.06	0.58/–0.67	0.64/–0.40

[a] $R1 = \Sigma||F_o|-|F_c||/\Sigma|F_o|$. [b] $wR2 = [\Sigma w(F_o^2-F_c^2)^2/\Sigma w(F_o^2)^2]^{1/2}$, $w = 4F_o^2/[pF_o^2 + q\sigma(F_o^2)]$ [p = 0 (**1a**), 0.0004 (**1c**); q = 1.8 (**1a**), 1 (**1c**)]; $w = 1/[\sigma^2(F_o^2) + (0.0425P)^2 + 15.5765P]$, $P = (\mathrm{Max}(F_o^2, 0) + 2\,F_c^2)/3$ (**1b**). [c] GOF = $[\Sigma w(F_o^2-F_c^2)^2/(N_o-N_{params})]^{1/2}$.

Supplementary Table 2 | X-ray crystallographic data for 3b·1/3C_6H_{14}, 3c·0.5CH_2Cl_2.

	**3b**·1/3C_6H_{14}	**3c**·0.5CH_2Cl_2
chemical formula	$C_{25}H_{50.67}Cl_3MoN_2P_2$	$C_{27.5}H_{49}Cl_4MoN_2P_2$
CCDC number	1482258	1482259
formula weight	643.61	707.40
dimensions of crystals	0.07 × 0.07 × 0.05	0.20 × 0.15 × 0.07
crystal color, habit	orange, block	orange, platelet
crystal system	trigonal	monoclinic
space group	$R\overline{3}$	$P2_1/n$
a, Å	25.9925(13)	22.2435(10)
b, Å	25.9925(13)	11.8030(5)
c, Å	24.7973(6)	25.2407(12)
α, deg	90	90
β, deg	90	90.388(6)
γ, deg	120	90
V, $Å^3$	14508.8(11)	6626.6(5)
Z	18	8
ρ_{calcd}, g cm^{-3}	1.326	1.418
$F(000)$	6078.06	2944.00
μ, cm^{-1}	7.696	8.340
trans. factors range	0.653–0.962	0.332–0.943
no. reflections measured	46445	53829
no. unique reflections	7315 (R_{int} = 0.1093)	14631 (R_{int} = 0.1268)
no. parameters refined	333	756
$R1$ ($I > 2\sigma(I)$)[a]	0.0622	0.1267
$wR2$ (all data)[b]	0.1233	0.1610
GOF (all data)[c]	1.000	1.000
max diff peak / hole, e $Å^{-3}$	1.66/–0.66	1.26/–1.32

[a] $R1 = \Sigma||F_o|-|F_c||/\Sigma|F_o|$. [b] $wR2 = [\Sigma w(F_o^2-F_c^2)^2/\Sigma w(F_o^2)^2]^{1/2}$, $w = 4F_o^2/q\sigma(F_o^2)$ [q = 3.88 (**3b**·1/3C_6H_{14}), 2.815 (**3c**·0.5CH_2Cl_2)]. [c] GOF = $[\Sigma w(F_o^2-F_c^2)^2/(N_o-N_{params})]^{1/2}$.

Supplementary Table 3 | Selected bond lengths and angles of 1a

Bond lengths (Å)		Bond angles (deg)	
Mo(1)—C(1)	2.064(2)	P(1)—Mo(1)—P(2)	153.95(3)
Mo(1)—P(1)	2.4687(9)	Mo(1)—N(3)—N(4)	177.9(2)
Mo(1)—P(2)	2.4718(10)	Mo(1)—N(5)—N(6)	178.0(3)
Mo(1)—N(3)	2.025(3)	Mo(1)—N(7)—N(7*)	178.0(3)
Mo(1)—N(5)	2.032(3)	C(1)—Mo(1)—N(3)	100.83(12)
Mo(1)—N(7)	2.0769(18)	C(1)—Mo(1)—N(5)	82.96(12)
N(3)—N(4)	1.132(4)	C(1)—Mo(1)—N(7)	173.08(12)
N(5)—N(6)	1.129(4)		
N(7)—N(7*)	1.130(2)		

Supplementary Table 4 | Selected bond lengths and angles of 1b.

Bond lengths (Å)		Bond angles (deg)	
Mo(1)—C(1)	2.153(4)	P(1)—Mo(1)—P(2)	163.18(8)
Mo(1)—P(1)	2.5365(11)	Mo(1)—N(3)—N(4)	179.8(3)
Mo(1)—P(2)	2.5445(11)	Mo(1)—N(5)—N(6)	179.8(3)
Mo(1)—N(3)	2.005(3)	Mo(1)—N(7)—N(7*)	179.6(3)
Mo(1)—N(5)	2.003(4)	C(1)—Mo(1)—N(3)	90.70(13)
Mo(1)—N(7)	2.109(3)	C(1)—Mo(1)—N(5)	91.44(13)
N(3)—N(4)	1.129(5)	C(1)—Mo(1)—N(7)	179.55(14)
N(5)—N(6)	1.127(5)		
N(7)—N(7*)	1.154(4)		
Mo(2)—C(24)	2.149(7)	P(3)—Mo(2)—P(3*)	161.38(10)
Mo(2)—P(3)	2.5304(13)	Mo(2)—N(9)—N(10)	177.9(3)
Mo(2)—N(9)	2.012(4)	Mo(2)—N(11)—N(11*)	180.0
Mo(2)—N(11)	2.108(4)	C(24)—Mo(2)—N(9)	92.70(9)
N(9)—N(10)	1.124(6)	C(24)—Mo(2)—N(11)	180.0
N(11)—N(11*)	1.136(6)		

Supplementary Table 5 | Selected bond lengths and angles of 1c.

Bond lengths (Å)		Bond angles (deg)	
Mo(1)—C(1)	2.058(2)	P(1)—Mo(1)—P(1*)	150.842(19)
Mo(1)—P(1)	2.4840(4)	Mo(1)—N(2)—N(3)	178.12(18)
Mo(1)—N(2)	2.0272(15)	Mo(1)—N(4)—N(4*)	180.0
Mo(1)—N(4)	2.0803(17)	C(1)—Mo(1)—N(2)	92.50(5)
N(2)—N(3)	1.119(3)	C(1)—Mo(1)—N(4)	180.0
N(4)—N(4*)	1.134(2)		

Supplementary Table 6 | Selected bond lengths and angles of 3b.

Bond lengths (Å)		Bond angles (deg)	
Mo(1)—C(1)	2.4143(12)	P(1)—Mo(1)—P(2)	158.07(4)
Mo(1)—P(1)	2.6867(14)	C(1)—Mo(1)—Cl(1)	92.17(13)
Mo(1)—P(2)	2.6418(15)	C(1)—Mo(1)—Cl(2)	177.28(14)
Mo(1)—Cl(1)	2.4143(12)	C(1)—Mo(1)—Cl(3)	95.93(13)
Mo(1)—Cl(2)	2.4599(10)	Cl(1)—Mo(1)—Cl(2)	85.15(4)
Mo(1)—Cl(3)	2.4396(14)	Cl(1)—Mo(1)—Cl(3)	171.91(4)
		Cl(2)—Mo(1)—Cl(3)	86.76(4)
		C(1)—Mo(1)—P(1)	79.26(13)
		C(1)—Mo(1)—P(2)	78.82(13)

Supplementary Table 7 | Selected bond lengths and angles of 3c.

Bond lengths (Å)		Bond angles (deg)	
Mo(1)—C(1)	2.126(7)	P(1)—Mo(1)—P(2)	151.74(6)
Mo(1)—P(1)	2.6014(19)	C(1)—Mo(1)—Cl(1)	76.07(17)
Mo(1)—P(2)	2.6235(19)	C(1)—Mo(1)—Cl(2)	166.44(17)
Mo(1)—Cl(1)	2.4285(17)	C(1)—Mo(1)—Cl(3)	105.39(17)
Mo(1)—Cl(2)	2.437(2)	Cl(1)—Mo(1)—Cl(2)	91.31(6)
Mo(1)—Cl(3)	2.4072(17)	Cl(1)—Mo(1)—Cl(3)	176.38(6)
		Cl(2)—Mo(1)—Cl(3)	87.50(6)
		C(1)—Mo(1)—P(1)	75.90(18)
		C(1)—Mo(1)—P(2)	76.08(18)
Mo(2)—C(28)	2.123(6)	P(3)—Mo(2)—P(4)	149.13(5)
Mo(2)—P(3)	2.6150(18)	C(28)—Mo(2)—Cl(4)	74.93(18)
Mo(2)—P(4)	2.6091(18)	C(28)—Mo(2)—Cl(5)	163.34(18)
Mo(2)—Cl(4)	2.4534(18)	C(28)—Mo(2)—Cl(6)	109.38(18)
Mo(2)—Cl(5)	2.4572(18)	Cl(4)—Mo(2)—Cl(5)	88.66(6)
Mo(2)—Cl(6)	2.377(2)	Cl(4)—Mo(2)—Cl(6)	175.05(6)
		Cl(5)—Mo(2)—Cl(6)	87.15(6)
		C(28)—Mo(2)—P(3)	75.08(18)
		C(28)—Mo(2)—P(4)	75.77(18)

Supplementary Table 8 | Catalytic reaction using larger amounts of [LutH]OTf and CrCp*$_2$ Catalytic reactions were performed in the presence of [LutH]OTf (1.92 mmol) and CrCp*$_2$ (1.44 mmol) using typical experimental procedure. These results for the various volume of a toluene solution of CrCp*$_2$ (4.0 mL to 6.0 mL), drop time of a toluene solution of CrCp*$_2$ (30 to 120 min; total reaction time is 20 h), and catalyst loading (0.001 or 0.002 mmol) are shown.

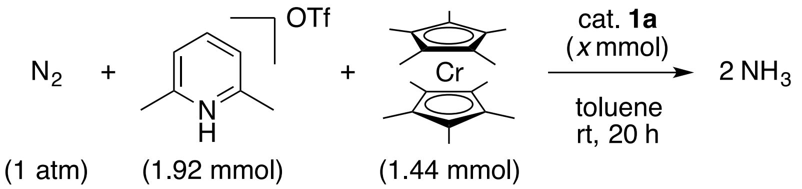

run	volume of CrCp*$_2$ solution (mL)	drop time (min)	x (mmol)	NH_3 (equiv)[a]	NH_3 (%)[b]	H_2 (equiv)[a]	H_2 (%)[b]
1	4.0	60	0.0021	1.2×10^2	50	8.8	3
2	4.0	30	0.0010	1.4×10^2	29	1.2×10^2	17
3	4.0	60	0.0010	1.2×10^2	25	2.3×10^2	32
4[c]	4.0	60	0.0010	1.3×10^2	28	2.2×10^2	31
5	4.0	120	0.0010	63	13	63	9
6	5.0	60	0.0010	$(2.0\pm0.2)\times10^2$	42±5	$(1.0\pm0.4)\times10^2$	14±6
7	6.0	60	0.0010	1.4×10^2	30	2.2×10^2	32

[a] Equivs based on **1a**.
[b] Yields based on CrCp*$_2$.
[c] **1a** was used after it was dried under vacuum for 20 h.

Supplementary Table 9 | Catalytic reaction using 1c as a catalyst. Catalytic reactions of **1c** were performed in the presence of [LutH]OTf (1.92 mmol) and $CrCp^*_2$ (1.44 mmol) using typical experimental procedure. These results for volume of a toluene solution of $CrCp^*_2$ (4.0 mL or 5.0 mL) and the various drop time of a toluene solution of $CrCp^*_2$ (60 or 300 min; total reaction time is 20 h) are shown.

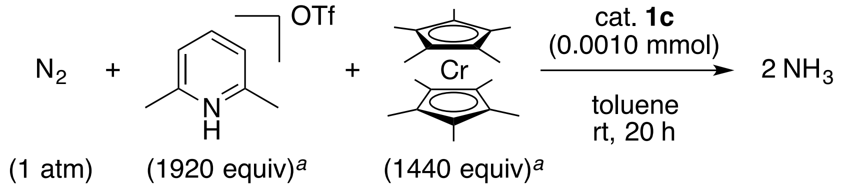

run	volume of $CrCp^*_2$ solution (mL)	drop time (min)	NH_3 (equiv)[a]	NH_3 (%)[b]	H_2 (equiv)[a]	H_2 (%)[b]
1	4.0	60	1.6×10^2	33	1.8×10^2	24
2	4.0	300	1.9×10^2	39	1.8×10^2	25
3	5.0	300	2.3×10^2	48	1.2×10^2	16

[a] Equivs based on **1c**.
[b] Yields based on $CrCp^*_2$.

Supplementary Table 10 | The amount of ammonia and dihydrogen produced in the catalytic reaction employing 1a and 1c. Catalytic reactions were performed in the presence of [LutH]OTf (0.96 mmol), $CrCp^*_2$ (0.72 mmol), and catalyst **1a** or **1c** (0.0033 mmol) using typical experimental procedure.

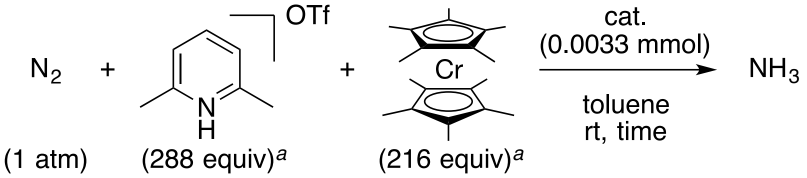

	**1a**		**1c**	
time (h)	NH_3 (equiv)[a]	H_2 (equiv)[a]	NH_3 (equiv)[a]	H_2 (equiv)[a]
0.33	12	6.3	17	0.6
0.67	27	11	40	3.4
1	42	20	53	4.8
2	40	19	52	8.3
20	46	12	53	6.7

[a] Equivs based on catalyst.

Supplementary methods.

General.

^{1}H NMR (270 MHz), ^{31}P{^{1}H} NMR (109 MHz), ^{13}C{^{1}H} NMR (68 MHz), and ^{15}N{^{1}H} NMR (27 MHz) spectra were recorded on a JEOL Excalibur 270 spectrometer in suitable solvents, and spectra were referenced to residual solvent (^{1}H, ^{13}C{^{1}H}) or external standard (^{31}P{^{1}H}: H_3PO_4, ^{15}N{^{1}H}: CH_3NO_2). IR spectra were recorded on a JASCO FT/IR 4100 Fourier Transform infrared spectrometer. Absorption spectra were recorded on a Shimadzu MultiSpec-1500. Evolved dihydrogen was quantified by a gas chromatography using a Shimadzu GC-8A with a TCD detector and a SHINCARBON ST (6 m × 3 mm). Elemental analyses were performed at Microanalytical Center of The University of Tokyo. Mass spectra were measured on a JEOL JMS-700 mass spectrometer. Melting points were measured on a Stanford Research Systems OptiMelt.

All manipulations were carried out under an atmosphere of nitrogen by using standard Schlenk techniques or glovebox techniques unless otherwise stated. Toluene was distilled from a dark-blue Na/benzophenone ketyl solution and degassed, and stored over molecular sieves 4A in a nitrogen-filled glove box. Other solvents were dried by general methods, and degassed before use. $CoCp_2$ (Aldrich) was sublimed before use. *o*-$C_6H_4(NHCH_2P^tBu_2)_2$ (**4a**),[1] $^tBu_2P(CH_2)_2NH_2$ (**8b**),[2] [$MoCl_3$(thf)$_3$],[3] [LutH]OTf,[4] $CrCp^*_2$,[5] and $CoCp^*_2$[6] [LutH]BAr^F_4 (Ar^F = 3,5-$(CF_3)_2C_6H_3$),[7] were prepared according to the literature methods. [ColH]OTf, [PicH]OTf were prepared in a method similar to [LutH]OTf. All the other reagents were commercially available.

Preparation of 4c

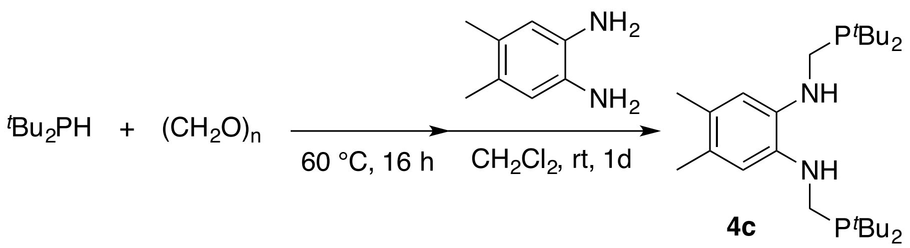

Compound **4c** was prepared according to a similar procedure to **4a**.[1] A mixture of ^{t}Bu$_2$PH (2.02 g, 13.8 mmol) and paraformaldehyde (398 mg, 13.3 mmol) was stirred at 60 °C for 16 h. CH_2Cl_2 (10 mL) and 4,5-dimethyl-1,2-diaminobenzene (903 mg, 6.63 mmol) were added to the resulting liquid and stirred at room temperature for one day. The solvent was removed under vacuum. The resulting solid was extracted with hexane (3 mL x 7) and recrystallized from hexane at –30 °C to give an analytically pure **4c** as colorless crystals (1.80 g, 3.98 mmol, 60%).

^{1}H NMR (C_6D_6): δ 6.84 (s, 2H, Ar*H*), 3.53 (br s, 2H, N*H*), 3.28 (s, 4H, NCH_2P), 2.30 (s, 6H, ArCH_3), 1.11 (d, $^3J_{P-H}$ = 10.8 Hz, 36H, P^t*Bu*$_2$). ^{13}C{^{1}H} NMR (C_6D_6): δ 137.0 (d, $^3J_{P-C}$ = 12.3 Hz, NH*C*CH), 126.5 (s, Ar), 114.6 (s, Ar), 39.6 (d, $^1J_{P-C}$ = 15.1 Hz, *C*H$_2$P), 31.2 (d, $^1J_{P-C}$ = 21.8 Hz, P*C*(CH$_3$)$_3$), 29.9 (d, $^2J_{P-C}$ = 13.4 Hz, C(*C*H$_3$)$_3$), 19.6 (s, Ar–*C*H$_3$). ^{31}P{^{1}H} NMR (C_6D_6): δ 28.8 (s, *P*^{t}Bu$_2$). Anal. Calcd. for $C_{26}H_{50}N_2P_2$: C, 68.99; H, 11.13; N, 6.19. Found: C, 69.18; H, 11.22; N, 6.33.

Preparation of 5a and 5c

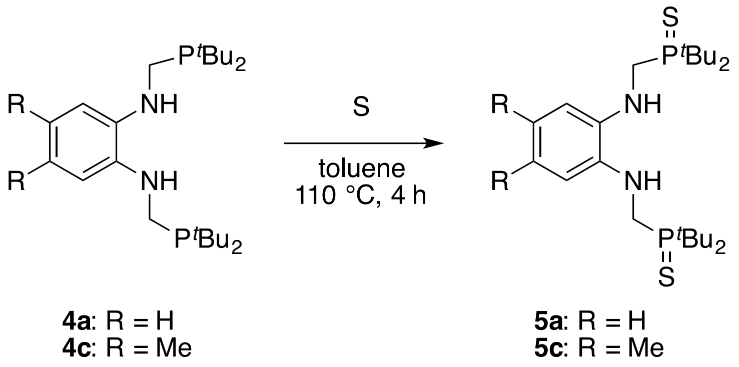

A typical procedure for the preparation of **5a** is described below. A solution of *o*-C$_6$H$_4$(NHCH$_2$P^{t}Bu$_2$)$_2$ (**4a**, 2.63 g, 6.19 mmol) and elemental sulfur (397 mg, 12.4 mmol) in toluene (35 mL) was stirred at 110 °C for 4 h. The solvent was removed under vacuum and the residue was purified by column chromatography on silica gel (CH_2Cl_2) to give an analytically pure **5a** as a white solid (2.70 g, 5.52 mmol, 89%).

5a: M.p. = 183.0 °C (decomp.). ^{1}H NMR ($CDCl_3$): δ 6.84–6.79 (m, 2H, Ar*H*), 6.72–6.67 (m, 2H, Ar*H*), 4.61–4.56 (br m, 2H, N*H*), 3.37 (d, $^2J_{P–H}$ = 5.4 Hz, 2H, NC*H*$_2$P), 3.35 (d, $^2J_{P–H}$ = 5.4 Hz, 2H, NC*H*$_2$P), 1.40 (d, $^3J_{P–H}$ = 15.1 Hz, 36H, P^t*Bu*$_2$). ^{13}C{^{1}H} NMR ($CDCl_3$): δ 138.0 (d, $^3J_{P–C}$ = 10.6 Hz, N*C*CH), 119.4 (s, Ar), 111.6 (s, Ar), 37.6 (d, $^1J_{P–C}$ = 40.7 Hz, N*C*H$_2$P), 36.0 (d, $^1J_{P–C}$ = 46.2 Hz, P*C*(CH_3)$_3$), 27.6 (d, $^2J_{P–C}$ = 1.1 Hz, PC(*C*H$_3$)$_3$). ^{31}P{^{1}H} NMR ($CDCl_3$): δ 78.3 (s, *P*^{t}Bu$_2$). Anal. Calcd. for $C_{24}H_{46}N_2P_2S_2$: C, 58.98; H, 9.49; N, 5.73. Found: C, 58.84; H, 9.68; N, 5.71.

5c: A white solid. 96% yield. M.p. = 138.5 °C (decomp.). ^{1}H NMR ($CDCl_3$): δ 6.50 (s, 2H, Ar*H*), 4.59 (br s, 2H, N*H*) 3.38 (d, $^2J_{P–H}$ = 6.5 Hz, 4H, NC*H*$_2$P), 2.19 (s, 6H, ArC*H*$_3$), 1.40 (d, $^3J_{P–H}$ = 15.1 Hz, 36H, P^t*Bu*$_2$). ^{13}C{^{1}H} NMR ($CDCl_3$): δ 136.2 (d, $^3J_{P–C}$ = 11.1 Hz, N*C*CH), 127.1 (s, Ar), 114.6 (s, Ar), 37.6 (d, $^1J_{P–C}$ = 40.7 Hz, N*C*H$_2$P), 36.8 (d, $^1J_{P–C}$ = 46.2 Hz, P*C*(CH_3)$_3$), 27.6 (d, $^2J_{P–C}$ = 1.1 Hz, PC(*C*H$_3$)$_3$), 19.1 (s, Ar–*C*H$_3$). ^{31}P{^{1}H} NMR ($CDCl_3$): δ 78.3 (s, *P*^{t}Bu$_2$). Anal. Calcd. for $C_{26}H_{50}N_2P_2S_2$: C, 60.43; H, 9.75; N, 5.42. Found: C, 60.18; H, 9.83; N, 5.40.

Preparation of 6a and 6c

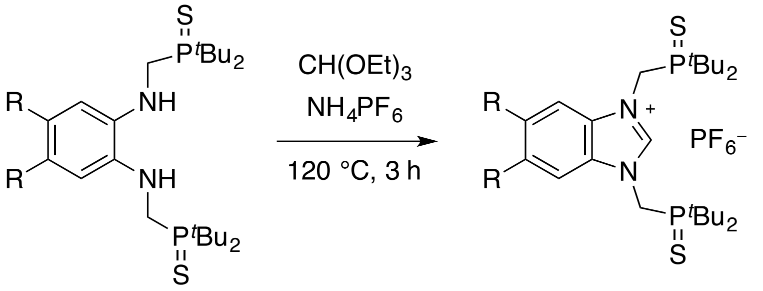

5a: R = H
5c: R = Me

6a: R = H
6c: R = Me

A typical procedure for the preparation of **6a** is described below. To a mixture of **5a** (2.56 g, 5.25 mmol) and NH_4PF_6 (864 mg, 5.30 mmol) was added CH(OEt)$_3$ (13 mL, 78 mmol) and stirred at 120 °C for 3 h. The volatiles were removed under vacuum. The resultant solid was washed with CH_2Cl_2/Et_2O = 2/5 (v/v, 35 mL) and Et_2O (5 mL x 2) and dried under vacuum to give an analytically pure **6a** as a white solid (3.23 g, 5.01 mmol, 95%).

6a: ^{1}H NMR ($CDCl_3$): δ 9.87 (s, 1H, NC*H*N), 7.95–7.90 (m, 2H, Ar*H*), 7.66–7.61 (m, 2H, Ar*H*), 4.95 (d, $^2J_{P–H}$ = 3.0 Hz, 4H, NC*H*$_2$P), 1.42 (d, $^3J_{P–H}$ = 15.7 Hz, 36H, P^t*Bu*$_2$). ^{13}C{^{1}H} NMR ($CDCl_3$): δ 143.3 (s, N*C*HN), 131.3 (s, Ar), 127.3 (s, Ar), 113.8 (s, Ar), 40.2 (d, $^1J_{P–C}$ = 32.3 Hz, N*C*H$_2$P), 38.9 (d, $^1J_{P–C}$ = 39.0 Hz, P*C*(CH_3)$_3$), 27.5 (s, PC(*C*H$_3$)$_3$). ^{31}P{^{1}H} NMR ($CDCl_3$): δ 78.3 (s, *P*^{t}Bu$_2$), –131.7 – –157.8 (m, *P*F$_6^-$). Anal. Calcd. for $C_{25}H_{45}F_6N_2P_3S_2$: C, 46.58; H, 7.04; N, 4.35. Found: C, 46.51; H, 7.10; N, 4.43.

6c: A white solid. 83% yield. ^{1}H NMR ($CDCl_3$): δ 9.79 (s, 1H, NC*H*N), 7.65 (s, 2H, Ar*H*), 4.89 (d, $^2J_{P–H}$ = 3.0 Hz, 4H, NC*H*$_2$P), 2.45 (s, 6H, ArC*H*$_3$), 1.41 (d, $^3J_{P–H}$ = 15.7 Hz, 36H, P^t*Bu*$_2$). ^{13}C{^{1}H} NMR ($CDCl_3$): δ 141.6 (s, Ar), 137.8 (s, Ar), 129.7 (s, Ar), 113.4 (s, Ar), 40.0 (d, $^1J_{P–C}$ = 32.3 Hz, N*C*H$_2$P), 38.8 (d, $^1J_{P–C}$ = 39.1 Hz, P*C*(CH_3)$_3$), 27.5 (s, PC(*C*H$_3$)$_3$), 20.6 (s, Ar–*C*H$_3$). ^{31}P{^{1}H} NMR ($CDCl_3$): δ 78.1 (s, *P*^{t}Bu$_2$), –131.7 – –157.8 (m, *P*F$_6^-$). Anal. Calcd. for $C_{27}H_{49}F_6N_2P_3S_2$: C, 48.21; H, 7.34; N, 4.16. Found: C, 48.00; H, 7.46; N, 4.47.

Preparation of 9b

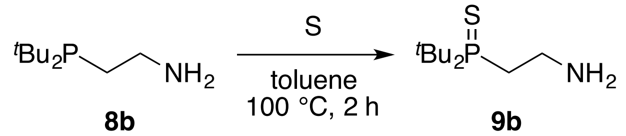

A solution of **8b** (730 mg, 3.86 mmol) and elemental sulfur (192 mg, 6.02 mmol) in toluene (20 mL) was stirred at 100 °C for 2 h. The solvent was removed under vacuum and the resulting oil was purified by column chromatography on silica gel (CH_2Cl_2 → CH_2Cl_2/MeOH 10/1 (v/v) → MeOH) and the resulting mixture was washed with Et_2O to afford **9b** as a white solid (347 mg, 1.57 mmol, 41%).

M.p. = ca. 185 °C (sublimed). ^{1}H NMR ($CDCl_3$): δ 3.48 (dt, 2H, $^3J_{P–H}$ = 16.7 Hz, and $^3J_{H–H}$ = 6.1 Hz, NCH_2), 2.48–2.41 (m, 2H, PCH_2), 1.35 (d, $^3J_{P–H}$ = 15.4 Hz, 18H, P^tBu_2). ^{13}C{^{1}H} NMR ($CDCl_3$): δ 38.2 (d, $^1J_{P–C}$ = 41.3 Hz, $PC(CH_3)_3$), 37.7 (s, NCH_2), 27.0 (d, $^2J_{P–C}$ = 1.2 Hz, $PC(CH_3)_3$), 18.3 (d, $^1J_{P–C}$ = 42.4 Hz, PCH_2). ^{31}P{^{1}H} NMR ($CDCl_3$): δ 74.5 (s, P^tBu_2). HRMS (FAB) Calcd. for $C_{10}H_{25}NPS$ $[M+H]^+$: 222.1445. Found: 222.1443.

Preparation of 6b

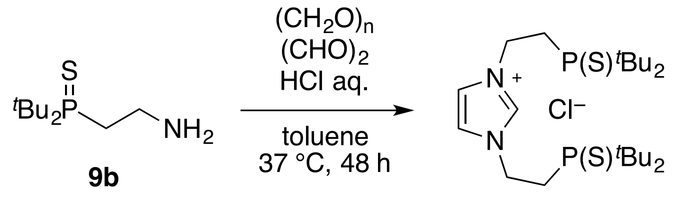

6b

To a suspension of **9b** (280 mg, 1.26 mmol) in toluene (6 mL) was added paraformaldehyde (38.1 mg, 1.27 mmol) and stirred at room temperature for 30 minutes. The resulting suspension was cooled to 0 °C, a suspension of **9b** (280 mg, 1.26 mmol) in toluene (6 mL) was added and stirred for another 30 minutes. Aqueous HCl (4.0 M, 0.32 mL, 1.3 mmol) was added to the suspension and allowed to warm to room temperature. After stirring for 1 h, aqueous glyoxal solution (8.8 M in water, 0.15 mL, 1.3 mmol) was added and the resulting suspension was stirred at 37 °C for 48 h. Et_2O and saturated aqueous $NaHCO_3$ solution was added to the suspension and the organic layer was removed. The suspension was washed with Et_2O (10 mL x 3). The solid was dissolved in CH_2Cl_2 (45 mL), extracted with CH_2Cl_2 (20 mL x 2) and the combined solution was dried over anhydrous $MgSO_4$. After filtration, the solvent was evaporated and dried under vacuum to give **6b** as a white solid (351 mg, 0.683 mmol, 54%).

^{1}H NMR ($CDCl_3$): δ 11.26 (s, 1H, NCHN), 7.58 (s, 2H, NCHCHN), 4.66–4.57 (m, 4H, NCH_2), 2.67–2.58 (m, 4H, PCH_2), 1.26 (d, $^3J_{P–H}$ = 15.7 Hz, 36H, P^tBu_2). ^{13}C{^{1}H} NMR ($CDCl_3$): δ 138.6 (s, NCHN), 122.1 (s, NCHCHN), 46.7 (s, NCH_2), 38.0 (d, $^1J_{P–C}$ = 41.3 Hz, $PC(CH_3)_3$), 27.0 (d, $^2J_{P–C}$ = 1.1 Hz, $PC(CH_3)_3$), 22.4 (d, $^1J_{P–C}$ = 39.1 Hz, PCH_2). ^{31}P{^{1}H} NMR ($CDCl_3$): δ 77.6 (s, P^tBu_2). Anal. Calcd. for $C_{23}H_{47}ClN_2P_2S_2$: C, 53.83; H, 9.23; N, 5.46. Found: C, 53.51; H, 8.91; N, 5.44.

Preparation of 7a, 7b and 7c

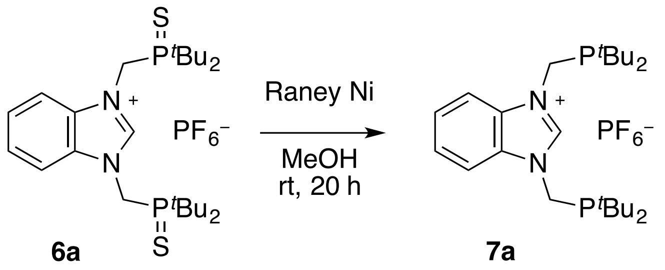

A typical procedure for the preparation of **7a** is described below. A suspension of **6a** (915 mg, 1.42 mmol) and Raney Nickel (13.6 g) in MeOH (35 mL) was stirred at room temperature for 20 h. The supernatant suspension was collected and the solvent was removed under vacuum. The residue was extracted with CH_2Cl_2 (5 mL x 4), filtered through Celite and the solvent was removed under vacuum. The resulting solid was washed with Et_2O (5 mL x 3) and then dried under vacuum to give an analytically pure **7a** as a yellow solid (478 mg, 0.824 mmol, 58%).

7a: ^{1}H NMR (THF-d_8): δ 9.71 (s, 1H, NC*H*N), 8.17–8.14 (m, 2H, Ar*H*), 7.67–7.63 (m, 2H, Ar*H*), 4.85 (d, $^2J_{P–H}$ = 1.4 Hz, 4H, NCH_2P), 1.22 (d, $^3J_{P–H}$ = 11.6 Hz, 36H, P^t*Bu*$_2$). ^{13}C{^{1}H} NMR (THF-d_8): δ 143.5 (t, $^3J_{P–C}$ = 9.8 Hz, N*C*HN), 133.1 (s, Ar), 127.2 (s, Ar), 115.4 (d, $^3J_{P–C}$ = 6.7 Hz, N*C*CH), 43.3 (d, $^1J_{P–C}$ = 28.4 Hz, N*C*H$_2$P), 32.7 (d, $^1J_{P–C}$ = 20.1 Hz, P*C*(CH_3)$_3$), 29.5 (d, $^2J_{P–C}$ = 13.4 Hz, PC(*C*H_3)$_3$). ^{31}P{^{1}H} NMR (THF-d_8): δ 26.8 (s, *P*^{t}Bu$_2$), –129.7 – –155.8 (m, *P*F_6^-). Anal. Calcd. for $C_{25}H_{45}F_6N_2P_3$: C, 51.72; H, 7.81; N, 4.83. Found: C, 51.85; H, 7.86; N, 5.09.

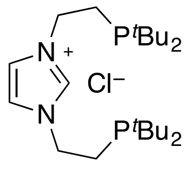

7b

7b: A yellow solid. 55% yield. ^{1}H NMR ($CDCl_3$): δ 11.31 (s, 1H, NC*H*N), 7.39 (s, 1H, NC*H*CHN), 7.38 (s, 1H, NCHC*H*N), 4.49 (dt, 4H, $^3J_{P–H}$ = 5.4 Hz, and $^3J_{H–H}$ = 7.4 Hz, NCH_2), 2.03 (dt, 4H, $^2J_{P–H}$ = 3.2 Hz, and $^3J_{H–H}$ = 7.4 Hz, PCH_2), 1.12 (d, $^3J_{P–H}$ = 11.3 Hz, 36H, P^t*Bu*$_2$). ^{13}C{^{1}H} NMR (THF-d_8): δ 140.5 (s, N*C*HN), 122.5 (s, N*CHC*HN), 51.1 (d, $^2J_{P–C}$ = 40.1 Hz, N*C*H_2), 32.0 (d, $^1J_{P–C}$ = 20.6 Hz, P*C*(CH_3)$_3$), 29.9 (d, $^2J_{P–C}$ = 13.9 Hz, PC(*C*H_3)$_3$), 23.6 (d, $^1J_{P–C}$ = 23.4 Hz, P*C*H_2). ^{31}P{^{1}H} NMR ($CDCl_3$): δ 21.0 (s, *P*^{t}Bu$_2$). HRMS (FAB) Calcd. for $C_{23}H_{47}N_2P_2$ [M–Cl]$^+$: 413.3215. Found: 413.3206.

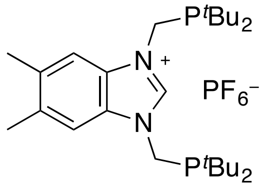

7c

7c: A yellow solid. 58% yield. ^{1}H NMR (THF-d_8): δ 9.58 (s, 1H, NC*H*N), 7.92 (s, 2H, Ar*H*), 4.76 (s, 4H, NCH_2P), 2.46 (s, 6H, ArCH_3), 1.22 (d, $^{3}J_{P-H}$ = 11.6 Hz, 36H, P$^{t}Bu_2$). ^{13}C{^{1}H} NMR (THF-d_8): δ 142.1 (t, $^{3}J_{P-C}$ = 11.7 Hz, N*C*HN), 137.6 (s, Ar), 131.6 (s, Ar), 114.7 (d, $^{3}J_{P-C}$ = 5.6 Hz, N*C*CH), 42.8 (d, $^{1}J_{P-C}$ = 27.3 Hz, N*C*H_2P), 32.6 (d, $^{1}J_{P-C}$ = 20.1 Hz, P*C*(CH_3)$_3$), 29.5 (d, $^{2}J_{P-C}$ = 13.9 Hz, PC(*C*H_3)$_3$), 20.3 (s, Ar–*C*H_3). ^{31}P{^{1}H} NMR (THF-d_8): δ 27.3 (s, P^{t}Bu$_2$), –142.7 (sept, $^{1}J_{P-F}$ = 710.6 Hz, PF_6^-). Anal. Calcd. for $C_{27}H_{49}F_6N_2P_3$: C, 53.28; H, 8.12; N, 4.60. Found: C, 53.35; H, 7.97; N, 4.81.

Preparation of Bim-PCP[1], Im-PCP[2], and Me-Bim-PCP[1]

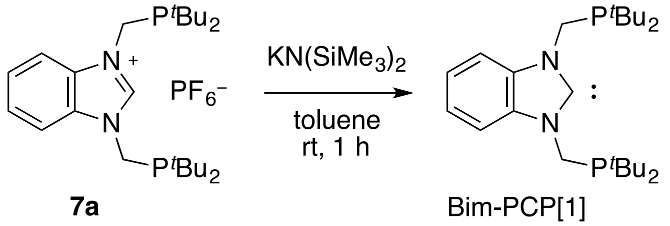

A typical procedure for the preparation of Bim-PCP[1] is described below. To a mixture of **7a** (36.3 mg, 0.0625 mmol) and $KN(SiMe_3)_2$ (17.3 mg, 0.0867 mmol) was added toluene (3 mL) and the resulting suspension was stirred for 1 hour at room temperature. The suspension was filtered through Celite and the filter cake was washed with toluene (1 mL x 2). The solvent of the combined solution was removed under vacuum to obtain a pale brown solid. The obtained PCP ligands were used in the subsequent reactions without further purification.

Bim-PCP[1]: ^{1}H NMR (C_6D_6): δ 7.92–7.87 (m, 2H, Ar*H*), 7.15–7.11 (m, 2H, Ar*H*), 4.71 (d, $^2J_{P–H}$ = 3.0 Hz, 4H, NCH_2P), 1.14 (d, $^3J_{P–H}$ = 10.5 Hz, 36H, P^t*Bu*$_2$). ^{13}C{^{1}H} NMR (THF-d_8): δ 228.8 (s, $C_{carbene}$), 136.5 (s, Ar), 121.1 (s, Ar), 112.7 (d, $^3J_{P–C}$ = 39.7 Hz, N*C*CH), 45.1 (d, $^1J_{P–C}$ = 23.9 Hz, NCH_2P), 32.3 (d, $^1J_{P–C}$ = 23.3 Hz, P*C*$(CH_3)_3$), 30.1 (d, $^2J_{P–C}$ = 13.4 Hz, PC$(CH_3)_3$). ^{31}P{^{1}H} NMR (C_6D_6): δ 14.5 (s, *P*^{t}Bu$_2$).

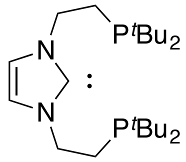

Im-PCP[2]

Im-PCP[2]: A yellow oil. ^{1}H NMR (THF-d_8): δ 6.92 (s, 2H, NC*H*C*H*N), 4.11–4.02 (m, 4H, NCH_2), 1.94–1.86 (m, 4H, PCH_2), 1.14 (d, $^3J_{P–H}$ = 10.8 Hz, 36H, P^t*Bu*$_2$). ^{13}C{^{1}H} NMR (THF-d_8): δ 214.8 (s, $C_{carbene}$), 118.8 (s, N*C*H*C*HN), 52.6 (d, $^2J_{P–C}$ = 39.7 Hz, NCH_2), 31.7 (d, $^1J_{P–C}$ = 21.8 Hz, P*C*$(CH_3)_3$), 29.8 (d, $^2J_{P–C}$ = 14.0 Hz, PC$(CH_3)_3$), 25.6 (d, $^1J_{P–C}$ = 23.5 Hz, PCH_2). ^{31}P{^{1}H} NMR (THF-d_8): δ 23.8 (s, *P*^{t}Bu$_2$).

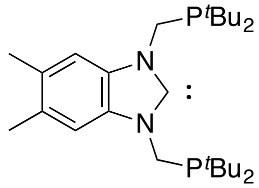

Me-Bim-PCP[1]

Me-Bim-PCP[1]: A brown solid. ^{1}H NMR (THF-d_8): δ 7.43 (s, 2H, Ar*H*), 4.66 (d, $^2J_{P–H}$ = 3.0 Hz, 4H, NCH_2P), 2.32 (s, 6H, ArCH_3), 1.22 (d, $^3J_{P–H}$ = 10.8 Hz, 36H, P^t*Bu*$_2$). ^{13}C{^{1}H} NMR (THF-d_8): δ 227.7 (s, $C_{carbene}$), 135.2 (s, Ar), 129.5 (s, Ar), 113.3 (d, $^3J_{P–C}$ = 10.0 Hz, N*C*CH), 45.1 (d, $^1J_{P–C}$ = 23.5 Hz, NCH_2P), 32.3 (d, $^1J_{P–C}$ = 22.9 Hz, P*C*$(CH_3)_3$), 30.1 (d, $^2J_{P–C}$ = 13.4 Hz, PC$(CH_3)_3$), 20.3 (s, Ar–CH_3). ^{31}P{^{1}H} NMR (THF-d_8): δ 16.4 (s, *P*^{t}Bu$_2$).

Reaction of 1a with $^{15}N_2$

To a 20 mL Schlenk flask was placed **1a**. The Schlenk flask was evacuated and $^{15}N_2$ gas was backfilled. Then C_6D_6 was added to the flask and the reaction mixture was stirred at room temperature for 1 h. The ^{15}N NMR spectrum of the reaction mixture was recorded on a JEOL JNM-ECS 400 spectrometer.

^{15}N NMR (C_6D_6, 41 MHz): δ 7.2 (s, Mo–*N*≡*N*–Mo), –13.0 (d, $^{1}J_{N–N}$ = 5.4 Hz, Mo–N≡*N*), –32.0 (br s, Mo–*N*≡N).

Reaction of 1b with $^{15}N_2$

^{15}N-enriched sample of **1b** was prepared by bubbling $^{15}N_2$ gas to a THF-d_8 solution of **1b**.

^{15}N NMR (THF-d_8): δ 1.2 (d, $^{1}J_{N–N}$ = 6.0 Hz, Mo–N≡*N*(equatorial)), –20.8 (d, $^{1}J_{N–N}$ = 6.1 Hz, Mo–N≡*N*(axial)), –27.8 (dt, $^{1}J_{N–N}$ = 6.0 Hz, and $^{2}J_{N–P}$ = 1.6 Hz, Mo–*N*≡N(equatorial)), –30.5 (dt, $^{1}J_{N–N}$ = 6.1 Hz, and $^{2}J_{N–P}$ = 2.3 Hz, Mo–*N*≡N(axial)).

Catalytic reduction of dinitrogen to ammonia under N_2 atmosphere

A typical experimental procedure for the catalytic reduction of dinitrogen into ammonia using the dinitrogen complex **1a** is described below. The reaction was carried out in a nitrogen-filled glove box. In a 50 mL Schlenk flask were placed **1a**·$1.3C_4H_8O·0.4C_6H_{14}$ (12.9 mg, 0.0097 mmol) and 2,6-lutidinium trifluoromethanesulfonate [LutH]OTf (247 mg, 0.960 mmol). Toluene (1.0 mL) was added under N_2 (1 atm), and then a solution of $CrCp*_2$ (232 mg, 0.719 mmol) in toluene (4.0 mL) was added to the stirred suspension in the Schlenk flask with a syringe pump at a rate of 4.0 mL per hour. After the addition of $CrCp*_2$, the mixture was further stirred at room temperature for 19 h. The amount of dihydrogen of the catalytic reaction was determined by GC analysis. The reaction mixture was evaporated under reduced pressure, and the distillate was trapped in dilute H_2SO_4 solution (0.5 M, 10.00 mL). Aqueous solution of potassium hydroxide (30 wt%; 5 mL) was added to the residue to fully liberate ammonia, and the mixture was distilled into another dilute H_2SO_4 solution (0.5 M, 10.00 mL). The amount of NH_3 present in each of the H_2SO_4 solutions was determined by the indophenol method.[8] The amount of ammonia collected before base distillation of the reaction mixture was 0.0338 mmol and that collected after base distillation was 0.146 mmol, respectively. The total amount of ammonia was 0.179 mmol (18.4 mol equiv per **1a**·$1.3C_4H_8O·0.4C_5H_{12}$). The yields of ammonium salt by using various reducing agents are listed in Table 1 in the Article.

For the experiments shown in Table 2, 2.0 μmol of catalyst **1a** was used, and the same procedure was applied.

For the experiments shown in Figure 5, A solution of $CrCp*_2$ (1.44 mmol) in 5 mL of toluene was added to a mixture of the catalyst (1.0 μmol) and [LutH]OTf (1.96 mmol) in 1 mL of toluene at room temperature over a period of 1 h (for **1a**) or 5 h (for **1c**), followed by stirring at room temperature for another 19 h (for **1a**) or 15 h (for **1c**) under 1 atm of dinitrogen gas. The quantification of dihydrogen and ammonia was carried out by the same procedure.

For the experiments shown in Supplementary Tables 8 and 9, A solution of $CrCp*_2$ (1.44 mmol) in the indicated volume of toluene was added to a mixture of the catalyst (1.0 μmol) and [LutH]OTf (1.96 mmol) in 1 mL of toluene at room temperature over a period of the indicated time (*n* hours), followed by stirring at room temperature for another (20–*n*) hours under 1 atm of dinitrogen gas. The quantification of dihydrogen and ammonia was carried out by the same procedure.

Time profiles for the formation of ammonia

A typical procedure is as follows. In a 50 mL Schlenk flask were placed **1a** or **1c** (0.0033 mmol) and [LutH]OTf (0.96 mmol). Toluene (1.0 mL) was added under N_2 (1 atm), and then a solution of $CrCp^*_2$ (0.72 mmol) in toluene (4.0 mL) was slowly added to the stirred mixture in the Schlenk flask with a syringe pump at a rate of 4.0 mL per hour. After the indicated time (0.33 h, 0.67 h, 1 h, 2 h, and 20 h), the amount of molecular dihydrogen produced in the catalytic reaction was determined by GC analysis. The amount of ammonia was determined by the indophenol method utilized procedure previously described.[8] The results are summarized in Supplementary Table 10.

Reactions of further addition of proton source and reductant

A solution of reductant ($CrCp^*_2$ for **1a** and $CoCp_2$ for **2**; 216 equiv/cat.) in toluene (4.0 mL) was added to a mixture of **1a** or **2** (0.0033 mmol for **1a** and 0.010 mmol for **2**) and [LutH]OTf (288 equiv/cat.) in toluene (1.0 mL) at a rate of 4.0 mL per hour at room temperature under 1 atm of dinitrogen gas, followed by stirring for 19 h, which are the same reaction conditions as the time profile experiments.[9] Then, [LutH]OTf (288 equiv/cat.) was added in one portion and another solution of the same reductant as the one added before (216 equiv/cat.) in toluene (4.0 mL) was added at a rate of 4.0 mL per hour, followed by stirring for another 1 h at room temperature under 1 atm of dinitrogen gas. The amount of molecular dihydrogen produced in the catalytic reaction was determined by GC analysis. The amount of ammonia was determined by the indophenol method utilized procedure previously described.[8] From the reaction with **1a**, 69 equiv of ammonia and 36 equiv of dihydrogen were obtained. From the reaction with **1b**, 21.0 equiv of ammonia and 63.5 equiv of dihydrogen were obtained**.**

Catalytic reaction under $^{15}N_2$ employing 1a as catalyst

In a 50 mL Schlenk flask were placed **1a**·$2C_6H_{14}$ (13.6 mg, 0.0099 mmol) and [LutH]OTf (247 mg, 0.959 mmol), and the mixture was exposed to $^{15}N_2$. Toluene (1 mL) was added to the mixture, and then a solution of $CrCp^*_2$ (232 mg, 0.720 mmol) in toluene (4 mL) was added to the stirred suspension in the Schlenk flask with a syringe pump at a rate of 4 mL per hour. After the addition of $CrCp^*_2$, the mixture was further stirred at room temperature for 19 h. KO^{t}Bu (4 mmol) in THF/MeOH (5/1 v/v, 6 mL) and was added to the resultant dark brown suspension, and the mixture was stirred for 1.5 h at room temperature. The volatile components in the mixture were collected by trap-to-trap distillation to the Schlenk flask to which was added HCl in Et_2O (2 M, 5 mL). The obtained white suspension was dried up under vacuum to afford a white solid which contains $^{15}NH_4Cl$ and [LutH]Cl. ^{1}H NMR spectrum of this solid is shown in Supplementary Figure 20.

^{1}H NMR (DMSO-d_6): δ 7.54 (d, $^1J_{N–H}$ = 71.0 Hz, $^{15}N\mathit{H}_4Cl$). $^{15}N\{^1H\}$ NMR (DMSO-d_6): δ–351.7 (s, $^{15}\mathit{N}H_4Cl$).

Reaction of 1a or 2 with [LutH]OTf.

A typical experimental procedure for the reaction of **1a** or **2** with [LutH]OTf is described below. To a mixture of **1a** (2.9 mg, 2.4 μmol) and [LutH]OTf (1.2 mg, 4.7 μmol) were added toluene (1 mL) and the reaction mixture was stirred for 2 min at room temperature. The supernatant solution of the reaction mixture was separated. Then the solution and the solid were dried under vacuum. The IR spectra of the solid from the supernatant solution and from the precipitate are shown in Supplementary Figure 7.

Supplementary Figures 8 and 9 show the IR spectra of the reaction mixture obtained from reactions of **2** and 2 equivalents or 5 equivalents of [LutH]OTf, respectively.

Supplementary Figures 10 and 11 show the IR spectra of the reaction mixture obtained from reactions of **1a** or **2** and 96 equivalents of [LutH]OTf, respectively.

Supplementary Figure 12a shows the IR spectra of 2,6-lutidine. Supplementary Figure 12b shows the IR spectra of [LutH]OTf.

X-ray crystallography.

Crystallographic data of **1a**, **1b**, **1c**, **3b**·1/3C_6H_{14}, **3c**·0.5CH_2Cl_2 are summarized in Supplementary Tables 1–2. The ORTEP drawings of **1c**, **3b**·1/3C_6H_{14}, **3c**·0.5CH_2Cl_2 are shown in Supplementary Figures 1–3 and selected bond lengths and angles of **1a**, **1b**, **1c**, **3b**·1/3C_6H_{14}, **3c**·0.5CH_2Cl_2 are shown in Supplementary Tables 3–7. Diffraction data for **1a**, **1b**, **1c**, **3b**·1/3C_6H_{14}, **3c**·0.5CH_2Cl_2 were collected for the 2θ range of 5˚ to 55˚ at –75 °C (for **1b**, **3b**·1/3C_6H_{14}), –100 °C (for **1c**), or –150 ˚C (for **1a, 3c**·0.5CH_2Cl_2) on a Rigaku RAXIS RAPID imaging plate area detector with graphite-monochromated Mo Kα radiation (λ = 0.71075 Å), with VariMax optics. Intensity data were corrected for Lorenz-polarization effects and for empirical absorption (ABSCOR). The structure solution and refinements were carried out by using the *CrystalStructure* crystallographic software package.[10] The positions of the non-hydrogen atoms were determined by direct methods (SIR 92[11] for **1c**, SIR 97[12] for **3b**·0.5CH_2Cl_2 and **3c**·0.5CH_2Cl_2, SIR2002[13] for **1a,** SHELXS97[14] for **1b**) and subsequent Fourier syntheses (DIRDIF-99[15] for **1a**, **1c**, **3b**·0.5CH_2Cl_2 and **3c**·0.5CH_2Cl_2, SHELXL 2014[14] for **1b**), and were refined on F_o^2 using all unique reflections by full-matrix least-squares with anisotropic thermal parameters except for some carbon atoms refined isotropically, including hexane carbon atoms (C(24)–C(28)) in **3b**·1/3C_6H_{14}. For the crystal of **1b**, we have investigated the raw diffraction data by using R-AXIS RAPID AUTO Ver. 3.11, which has ruled out the possibility of both non-merohedral and merohedral twins. Thus, choice of *Ccca* space group is appropriate for the crystal of **1b**. The unit cell of **1b** contains a solvent accessible void of 718 Å^3. The ^{1}H NMR and elemental analysis of the crystals indicated that the void was occupied by hexane molecules, which could not be located appropriately. The diffused electron density associated with the solvent molecule was removed by SQUEEZE routine in PLATON.[16] For the crystal of **3b**·1/3C_6H_{14}, we have constructed a model of hexane consisting of five located carbon atoms (C(24) to C(28)) solved as a rigid group with atom occupancies of 0.4 (6/15), where one hexane molecule (six carbon atoms) is disordered among three asymmetric units to form a 15-membered ring (C(24)–C(25)–C(26)–C(27)–C(28)–C(24)*–C(25)*–C(26)*–C(27)*–C(28)*–C(24)'–C(25)'–C(26)'–C(27)'–C(28)'–). Positions of hydrogen atoms of hexane cannot be refined because of this disorder. All the other hydrogen atoms were placed at the calculated positions with fixed isotropic parameter. For the crystal of **3c**·0.5CH_2Cl_2, we have investigated the raw diffraction data by using R-AXIS RAPID AUTO Ver. 3.11, which has ruled out the possibility of both non-merohedral and merohedral twins. As the beta angle of **3c** is almost 90 degrees, the R_{int} value is not good.

Computational Methods.

DFT calculations were performed with the Gaussian 09 program (Rev. C01).[17] The functional and basis sets adopted in the present study are basically similar to those in our previous works on the catalytic activity of dinitrogen-bridged dimolybdenum complexes[18] for comparison. Geometry optimizations were carried out with the B3LYP* functional. The B3LYP* functional is a reparametrized version of the B3LYP hybrid functional[19–22] developed by Reiher and co-workers for proper estimation of the energy difference between different spin states.[23,24] The B3LYP and B3LYP* energy expressions are given as Supplementary Equation (1):

$$E_{XC}^{B3LYP} = a_0 E_X^{HF} + (1–a_0)E_X^{LSDA} + a_X E_X^{B88} + a_c E_C^{LYP} + (1–a_c)E_C^{VWN} \quad (1)$$

where a_0 = 0.20 (B3LYP) or 0.15 (B3LYP*), a_x = 0.72, a_c = 0.81 and in which E_X^{HF} is the Hartree-Fock exchange energy; E_X^{LSDA} is the local exchange energy from the local spin density approximation; E_X^{B88} is Becke's gradient correction[19] to the exchange functional; E_C^{LYP} is the correlation functional developed by Lee *et al.*[21]; and E_C^{VMN} is the correlation energy calculated using the local correlation functional of Vosko, Wilk and Nusair (VWN)[22]. The SDD (Stuttgart/Dresden pseudopotentials) basis set[25,26] and 6-31G(d) basis sets[27–30] were employed for the Mo atoms and the other atoms, respectively. Optimized structures were confirmed to have the appropriate number of imaginary frequencies by vibrational analysis. Calculated vibrational frequencies were corrected with a scaling factor of 0.960.[31] An appropriate connection between a reactant and a product for each reaction step was confirmed by IRC[32–34] and quasi-IRC calculations. In the quasi-IRC calculation, the geometry of a transition state was at first shifted by perturbing the geometries very slightly along the reaction coordinate and released for equilibrium optimization. Cartesian coordinates of optimized intermediates and transition states are shown in Supplementary Tables 11–32. To discuss the energetics of the first protonation processes, single-point energy calculations at the optimized geometries were performed by using the 6-311+G(d,p) basis set[35–37] instead of 6-31G(d). Solvation effects of toluene were taken into account by using the polarizable continuum model (PCM).[38] Zero-point energy corrections were applied for energy changes and activation energies calculated for each reaction step in the protonation processes.

Supplementary References

1. Segawa, Y., Yamashita, M. & Nozaki, K. Syntheses of PBP pincer iridium complexes: a supporting boryl ligand. *J. Am. Chem. Soc.* **131**, 9201–9203 (2009).

2. Jia, W. *et al.* Aminophosphine ligands $R_2P(CH_2)_nNH_2$ and ruthenium hydrogenation catalysts $RuCl_2(R_2P(CH_2)_nNH_2)_2$. *Dalton Trans.* 8301–8307 (2009).

3. Stoffelbach, F., Saurenz, D. & Poli, R. Improved preparations of molybdenum coordination compounds from tetrachlorobis(diethyl ether)molybdenum(IV). *Eur. J. Inorg. Chem.* 2699–2703 (2001).

4. Arashiba, K., Miyake, Y. & Nishibayashi, Y. A molybdenum complex bearing PNP-type pincer ligands leads to the catalytic reduction of dinitrogen into ammonia. *Nat. Chem.* **3**, 120–125 (2011).

5. Robbins, J. L., Edelstein, N., Spencer, B. & Smart, J. C. Syntheses and electronic structures of decamethylmetallocenes. *J. Am. Chem. Soc.* **104**, 1882–1893 (1982).

6. Yandulov, D. V & Schrock, R. R. Synthesis of tungsten complexes that contain hexaisopropylterphenyl-substituted triamidoamine ligands, and reactions relevant to the reduction of dinitrogen to ammonia. *Can. J. Chem.* **83**, 341–357 (2005).

7. Yandulov, D. V. & Schrock, R. R. Reduction of dinitrogen to ammonia at a well-protected reaction site in a molybdenum triamidoamine complex. *J. Am. Chem. Soc.* **124**, 6252–6253 (2002).

8. Weatherburn, M. W. Phenol-hypochlorite reaction for determination of ammonia. *Anal. Chem.* **39**, 971–974 (1967).

9. Kuriyama, S. *et al.* Catalytic formation of ammonia from molecular dinitrogen by use of dinitrogen-bridged dimolybdenum–dinitrogen complexes bearin PNP-pincer ligands: remarkable effect of substituent at PNP-pincer ligand. *J. Am. Chem. Soc.* **136**, 9719–9731 (2014).

10. *CrystalStructure 4.0*: *Single Crystal Structure Analysis Software*; Rigaku Corp: Tokyo, Japan, and MSC: The Woodlands, TX, 2010.

11. Altomare, A., Cascarano, G., Giacovazzo, C. & Guagliardi, A. Completion and refinement of crystal structures with *SIR92*. *J. Appl. Crystallogr.* **26**, 343–350 (1993).

12. Altomare, A. et al. *SIR97*: a new tool for crystal structure determination and refinement. *J. Appl. Crystallogr.* **32**, 115–119 (1999).

13. Burla, M. C. *et al. SIR2002*: the program. *J. Appl. Crystallogr.* **36**, 1103 (2003).

14. Sheldrick, G. M. A short history of SHELX. *Acta crystallogr.* **A64**, 112-122 (2008).

15. Beurskens, P. T. *et al. The DIRDIF-99 Program System*; Crystallography Laboratory, University of Nijmegen: Nijmegen, The Netherlands, 1999.

16. Spek, A. L. *PLATON: A Multipurpose Crystallographic Tool.* Utrecht University: Utrecht, The Netherlands, 1998.

17. Frisch, M. J. *et al. Gaussian 09*, Revision C.01; Gaussian, Inc.: Wallingford CT, 2010.

18. Tanaka, H., Nishibayashi, Y. & Yoshizawa, K. Interplay between theory and experiment for ammonia

synthesis catalyzed by transition metal complexes. *Acc. Chem. Res.* **49**, 987–995 (2016).

19. Becke, A. D. Density-functional exchange-energy approximation with correct asymptotic behavior. *Phys. Rev.* A **38**, 3098–3100 (1988).

20. Becke, A. D. Density-functional thermochemistry. III. The role of exact exchange. *J. Chem. Phys.* **98**, 5648–5652 (1993).

21. Lee, C., Yang, W. & Parr, R. G. Development of the Colle-Salvetti correlation-energy formula into a functional of the electron density. *Phys. Rev. B* **37**, 785–789 (1988).

22. Vosko, S. H., Wilk, L. & Nusair, M. J. Accurate spin-dependent electron liquid correlation energies for local spin density calculations: a critical analysis. *Can. J. Phys.* **58**, 1200–1211 (1980).

23. Reiher, M., Salomon, O. & Hess, B. A. Reparameterization of hybrid functionals based on energy differences of states of different multiplicity. *Theor. Chem. Acc.* **107**, 48–55 (2001).

24. Reiher, M. Theoretical study of the $Fe(phen)_2(NCS)_2$ spin-crossover complex with reparametrized density functionals. *Inorg. Chem.* **41**, 6928–6935 (2002).

25. Dolg, M., Wedig, U., Stoll, H. & Preuß, H. Energy-adjusted *ab initio* pseudopotentials for the first row transition elements. *J. Chem. Phys*. **86**, 866–872 (1987).

26. Andrae, D., Häußermann, U., Dolg, M., Stoll, H. & Preuß, H. Energy-adjusted *ab initio* pseudopotentials for the second and third row transition elements. *Theor. Chim. Acta.* **77**, 123–141 (1990).

27. Ditchfield, R., Hehre, W. J. & Pople, J. A. Self-consistent molecular-orbital methods. IX. An extended Gaussian-type basis for molecular orbital studies of organic molecules. *J. Chem. Phys*. **54**, 724–728 (1971).

28. Hehre, W. J., Ditchfield, R. & Pople, J. A. Self-consistent molecular orbital methods. XII. Further extensions of Gaussian-type basis sets for use in molecular orbital studies of organic molecules. *J. Chem. Phys. 56*, 2257–2261 (1972).

29. Hariharan, P. C. & Pople, J. A. The influence of polarization functions on molecular orbital hydrogenation energies. *Theor. Chem. Acc.* **28**, 213–222 (1973).

30. Francl, M. M. *et al.* Self-consistent molecular orbital methods. XXIII. A polarization-type basis set for second-row elements. *J. Chem. Phys*. **77**, 3654–3665 (1982).

31. Scott, A. P. & Radom, L. Harmonic vibrational frequencies: an evaluation of Hartree-Fock, Møller-Plesset, quadratic configuration interaction, density functional theory, and semiempirical scale factors. *J. Phys. Chem*. **100**, 16502–16513 (1996).

32. Fukui, K. Formulation of the reaction coordinate. *J. Phys. Chem.* **74**, 4161–4163 (1970).

33. Fukui, K. The path of chemical reactions – the IRC approach. *Acc. Chem. Res.* **14**, 363–368 (1981).

34. Gonzalez, C. & Schlegel, H. B. Reaction path following in mass-weighted internal coordinates. *J. Phys. Chem.* **94**, 5523–5527 (1990).

35. Krishnan, R., Binkley, J. S., Seeger, R. & Pople, J. A. Self-consistent molecular orbital methods. XX. A basis set for correlated wave functions. *J. Chem. Phys.* **72**, 650–654 (1980).

36. McLean, A. D. & Chandler, G. S. Contracted Gaussian basis sets for molecular calculations. I. Second row atoms, $Z = 11–18$. *J. Chem. Phys.* **72**, 5639–5648 (1980).
37. Clark, T., Chandrasekhar, J., Spitznagel, G. W. & Schleyer, P. v. R. Efficient diffuse function-augmented basis sets for anion calculations. III. The 3-21+G basis set for first row elements, Li–F. *J. Comput. Chem.* **4**, 294–301 (1983).
38. Tomasi, J., Mennucci, B. & Cammi, R. Quantum mechanical continuum solvation models. *Chem. Rev.* **105**, 2999–3094 (2005).
