# Supplementary material for: Remarkable catalytic activity of dinitrogen-bridged dimolybdenum complexes bearing NHC-based PCP-pincer ligands toward nitrogen fixation
Source: Nat Commun. 2017 Apr 4;8:14874. doi: 10.1038/ncomms14874 (PMC5382288; doi:10.1038/ncomms14874)
Supplement: Supplementary Data 1 — Information of Cartesian Coordinates. [file ncomms14874-s2.docx]

**Cartesian coordinate of 1a.** Units are presented in Å.

SCF energy = -4224.65713232 hartree

ZPE = 1.342762 hartree

SCF energy (in toluene) = -4225.58055749 hartree

-------------------------------------------------------

Atom Coordinates (Angstroms)

X Y Z

--------------------------------------------------------

Mo -2.681687 -0.000438 -0.000337

P -3.319466 -1.806896 1.689472

P -3.318886 1.806320 -1.690071

N -2.556513 1.367098 1.497172

N -2.422046 2.144858 2.315962

N -2.556754 -1.368252 -1.497668

N -2.422384 -2.146275 -2.316214

N -0.573979 -0.000494 -0.000314

N 0.573955 -0.000431 -0.000094

N -5.617625 -0.512588 0.967788

N -5.617606 0.513345 -0.967812

C -2.546886 -1.995741 3.464944

C -1.338008 -2.951566 3.406618

C -3.527223 -2.458151 4.565300

C -2.014810 -0.609078 3.872113

C -3.739244 -3.593926 1.041575

C -4.301984 -4.562585 2.101945

C -4.814588 -3.463330 -0.062592

C -2.468719 -4.223671 0.427976

C -5.069121 -1.177270 2.138723

C -4.774400 0.000045 -0.000178

C -6.954942 -0.326332 0.628390

C -6.954932 0.328182 -0.627855

C -5.069042 1.177712 -2.138889

C -3.737487 3.593603 -1.042147

C -2.466545 4.222434 -0.428455

C -4.812986 3.463711 0.061954

C -4.299463 4.562677 -2.102526

C -2.546662 1.994802 -3.465720

C -1.337307 2.950036 -3.407583

C -2.015419 0.607894 -3.873134

C -3.527028 2.457875 -4.565767

H -0.792242 -2.896391 4.359958

H -1.630123 -3.996764 3.260477

H -0.633847 -2.672714 2.612349

H -2.989334 -2.484732 5.525244

H -4.364642 -1.760475 4.693225

H -3.937943 -3.456672 4.397704

H -1.271133 -0.240163 3.159664

H -2.809306 0.141399 3.952129

H -1.534314 -0.687706 4.859127

H -3.563359 -4.837547 2.861818

H -5.190129 -4.164398 2.609460

H -4.609356 -5.492652 1.600079

H -5.786645 -3.154700 0.342208

H -4.532390 -2.759187 -0.847783

H -4.956371 -4.448489 -0.531691

H -2.732500 -5.194107 -0.020009

H -2.037817 -3.603026 -0.360680

H -1.692466 -4.409833 1.176898

H -5.745409 -1.981058 2.453846

H -4.974617 -0.465041 2.969472

H -5.744919 1.981941 -2.453784

H -4.975241 0.465445 -2.969683

H -2.036308 3.601599 0.360415

H -1.689982 4.407820 -1.177256

H -2.729619 5.193183 0.019274

H -4.954088 4.448937 0.531124

H -5.785251 3.155820 -0.342915

H -4.531365 2.759329 0.847141

H -4.606138 5.492972 -1.600662

H -3.560624 4.837089 -2.862399

H -5.187899 4.165158 -2.610049

H -0.791925 2.894919 -4.361149

H -1.628910 3.995317 -3.260981

H -0.632963 2.670633 -2.613675

H -1.271511 0.238665 -3.161097

H -2.810284 -0.142244 -3.952731

H -1.535423 0.686294 -4.860406

H -4.364907 1.760718 -4.693514

H -3.937087 3.456637 -4.397978

H -2.989386 2.484210 -5.525856

Mo 2.681668 -0.000165 0.000126

P 3.319576 -1.806884 -1.689363

P 3.318714 1.806763 1.689727

N 2.556607 1.367206 -1.497545

N 2.422223 2.144844 -2.316464

N 2.556689 -1.367751 1.497685

N 2.422338 -2.145690 2.316310

N 5.617728 -0.512672 -0.967455

N 5.617453 0.513565 0.967987

C 2.547296 -1.995918 -3.464915

C 1.338354 -2.951676 -3.406615

C 3.527802 -2.458616 -4.564999

C 2.015393 -0.609295 -3.872435

C 3.739104 -3.593870 -1.041227

C 4.301788 -4.562737 -2.101439

C 4.814436 -3.463253 0.062958

C 2.468457 -4.223395 -0.427656

C 5.069366 -1.177461 -2.138405

C 4.774370 0.000237 0.000251

C 5.068759 1.178093 2.138909

C 3.737624 3.593915 1.041663

C 2.466806 4.222866 0.427850

C 4.813202 3.463772 -0.062324

C 4.299690 4.563002 2.102002

C 2.546268 1.995552 3.465231

C 1.336948 2.950809 3.406753

C 2.014952 0.608701 3.872762

C 3.526504 2.458754 4.565330

H 0.792782 -2.896698 -4.360075

H 1.630410 -3.996850 -3.260171

H 0.634044 -2.672631 -2.612550

H 2.990099 -2.485323 -5.525044

H 4.365321 -1.761053 -4.692889

H 3.938385 -3.457150 -4.397131

H 1.271502 -0.240253 -3.160278

H 2.809944 0.141141 -3.952308

H 1.535209 -0.688048 -4.859590

H 3.563159 -4.837758 -2.861288

H 5.189978 -4.164702 -2.608999

H 4.609068 -5.492750 -1.599418

H 5.786552 -3.154851 -0.341871

H 4.532348 -2.758942 0.848032

H 4.956052 -4.448354 0.532227

H 2.732111 -5.193724 0.020629

H 2.037417 -3.602530 0.360753

H 1.692338 -4.409719 -1.176682

H 5.745643 -1.981353 -2.453289

H 4.975087 -0.465345 -2.969278

H 5.744626 1.982334 2.453792

H 4.974799 0.465948 2.969791

H 2.036477 3.601994 -0.360949

H 1.690259 4.408462 1.176623

H 2.730048 5.193516 -0.019992

H 4.954536 4.448955 -0.531515

H 5.785367 3.155720 0.342661

H 4.531552 2.759417 -0.847517

H 4.606665 5.493159 1.600062

H 3.560833 4.837711 2.861738

H 5.187966 4.165346 2.609699

H 0.791331 2.895737 4.360186

H 1.628589 3.996077 3.260170

H 0.632800 2.671384 2.612679

H 1.271048 0.239427 3.160745

H 2.809789 -0.141461 3.952453

H 1.534926 0.687208 4.860010

H 4.364429 1.761662 4.693171

H 3.936511 3.457536 4.397526

H 2.988767 2.485096 5.525367

C -8.147675 -0.652840 1.271643

C -9.344984 -0.318860 0.624235

C -9.344980 0.322724 -0.622681

C -8.147666 0.655691 -1.270603

H -8.151901 -1.144535 2.241842

H -10.292000 -0.562283 1.101674

H -10.291995 0.566949 -1.099712

H -8.151891 1.147395 -2.240797

C 9.344869 0.322166 0.623771

C 8.147466 0.655471 1.271353

C 6.954821 0.328090 0.628374

C 6.955000 -0.326615 -0.627773

C 8.147824 -0.653455 -1.270690

C 9.345044 -0.319612 -0.623045

H 10.291820 0.566277 1.100989

H 8.151549 1.147331 2.241468

H 8.152190 -1.145298 -2.240812

H 10.292126 -0.563291 -1.100223

--------------------------------------------------------

**Cartesian coordinate of 1b.** Units are presented in Å.

SCF energy = -4074.67992035 hartree

ZPE = 1.367496 hartree

SCF energy (in toluene) = -4075.57655326 hartree

-------------------------------------------------------

Atom Coordinates (Angstroms)

X Y Z

--------------------------------------------------------

Mo -2.709825 -0.000053 -0.000002

P -3.105647 -2.151359 1.405754

P -3.105737 2.151378 -1.405707

N -2.628748 1.038250 1.726061

N -2.547242 1.619972 2.705520

N -2.628616 -1.038375 -1.726053

N -2.547059 -1.620155 -2.705473

N -0.577011 -0.000046 0.000032

N 0.576984 -0.000124 0.000043

N -5.748435 -0.197257 1.057972

N -5.748289 0.196916 -1.058415

C -1.780126 -2.698582 2.723787

C -0.522815 -3.223752 2.006891

C -2.222303 -3.742431 3.770700

C -1.387145 -1.411480 3.478142

C -3.739166 -3.741796 0.461079

C -4.174252 -4.921603 1.358349

C -4.963843 -3.314169 -0.380165

C -2.658103 -4.264675 -0.506006

C -4.596518 -1.859510 2.549136

C -5.306287 -0.504701 2.415123

C -4.882439 -0.000116 -0.000149

C -7.079997 -0.127590 0.666391

C -7.079906 0.127157 -0.667032

C -5.305976 0.504502 -2.415486

C -4.596444 1.859457 -2.549274

C -3.739613 3.741620 -0.460961

C -2.658677 4.264692 0.506158

C -4.964238 3.313650 0.380182

C -4.174956 4.921374 -1.358176

C -1.780081 2.698935 -2.723431

C -0.522917 3.224005 -2.006211

C -1.386905 1.412036 -3.478034

C -2.222150 3.743030 -3.770145

H 0.310521 -3.267238 2.722135

H -0.663529 -4.234570 1.606843

H -0.213720 -2.564747 1.188103

H -1.426067 -3.837143 4.524466

H -3.135836 -3.459338 4.308254

H -2.376844 -4.735913 3.342498

H -0.971716 -0.662161 2.800982

H -2.229688 -0.954369 4.013711

H -0.616587 -1.656532 4.223618

H -3.318500 -5.439046 1.802706

H -4.855638 -4.634136 2.168431

H -4.704993 -5.658457 0.736408

H -5.806448 -2.977240 0.236836

H -4.718585 -2.515281 -1.083861

H -5.312019 -4.181901 -0.960754

H -3.091210 -5.066260 -1.124500

H -2.291179 -3.490336 -1.181281

H -1.802641 -4.692806 0.024985

H -5.327375 -2.661828 2.399633

H -4.258499 -1.935536 3.588628

H -4.660284 0.302719 2.762013

H -6.201721 -0.501286 3.050401

H -7.895548 -0.257386 1.364542

H -7.895362 0.256857 -1.365313

H -4.659765 -0.302776 -2.762319

H -6.201307 0.500964 -3.050907

H -5.327478 2.661595 -2.399674

H -4.258381 1.935732 -3.588735

H -2.291425 3.490361 1.181267

H -1.803405 4.693232 -0.024818

H -3.092023 5.066008 1.124831

H -5.312654 4.181258 0.960810

H -5.806719 2.976569 -0.236910

H -4.718842 2.514762 1.083823

H -4.705648 5.658194 -0.736150

H -3.319347 5.438882 -1.802725

H -4.856492 4.633820 -2.168101

H 0.310498 3.267846 -2.721340

H -0.663814 4.234658 -1.605818

H -0.213849 2.564750 -1.187616

H -0.971414 0.662627 -2.801016

H -2.229358 0.954938 -4.013754

H -0.616330 1.657340 -4.223410

H -3.135607 3.460058 -4.307893

H -2.376765 4.736402 -3.341700

H -1.425820 3.837968 -4.523783

Mo 2.709806 -0.000032 0.000019

P 3.105725 -2.151324 -1.405749

P 3.105674 2.151411 1.405714

N 2.628611 1.038329 -1.726003

N 2.547022 1.620106 -2.705423

N 2.628712 -1.038380 1.726059

N 2.547170 -1.620163 2.705479

N 5.748362 -0.197043 -1.058111

N 5.748307 0.197092 1.058283

C 1.780147 -2.698737 -2.723643

C 0.522927 -3.223917 -2.006599

C 2.222329 -3.742688 -3.770451

C 1.387021 -1.411745 -3.478116

C 3.739468 -3.741694 -0.461129

C 4.174473 -4.921499 -1.358443

C 4.964259 -3.313979 0.379907

C 2.658565 -4.264604 0.506120

C 4.596490 -1.859350 -2.549234

C 5.306153 -0.504482 -2.415245

C 4.882411 0.000009 0.000064

C 7.079942 -0.127307 -0.666601

C 7.079907 0.127417 0.666826

C 5.306047 0.504650 2.415378

C 4.596467 1.859577 2.549195

C 3.739394 3.741707 0.460935

C 2.658396 4.264679 -0.506169

C 4.964029 3.313826 -0.380238

C 4.174682 4.921512 1.358110

C 1.780051 2.698831 2.723528

C 0.522793 3.223797 2.006399

C 1.387045 1.411876 3.478126

C 2.222089 3.742942 3.770238

H -0.310454 -3.267566 -2.721780

H 0.663760 -4.234668 -1.606425

H 0.213835 -2.564831 -1.187875

H 1.426057 -3.837565 -4.524157

H 3.135815 -3.459614 -4.308094

H 2.376960 -4.736100 -3.342112

H 0.971542 -0.662400 -2.801019

H 2.229498 -0.954609 -4.013767

H 0.616458 -1.656950 -4.223536

H 3.318684 -5.438960 -1.802707

H 4.855762 -4.634021 -2.168602

H 4.705299 -5.658339 -0.736558

H 5.806749 -2.977029 -0.237242

H 4.719078 -2.515078 1.083615

H 5.312571 -4.181674 0.960469

H 3.091805 -5.066130 1.124596

H 2.291669 -3.490259 1.181406

H 1.803068 -4.692820 -0.024747

H 5.327430 -2.661599 -2.399775

H 4.258417 -1.935399 -3.588707

H 4.660058 0.302894 -2.762070

H 6.201545 -0.500975 -3.050581

H 7.895463 -0.257029 -1.364801

H 7.895394 0.257161 1.365063

H 4.659887 -0.302656 2.762237

H 6.201411 0.501144 3.050753

H 5.327457 2.661741 2.399534

H 4.258473 1.935850 3.588678

H 2.291173 3.490295 -1.181237

H 1.803114 4.693191 0.024810

H 3.091673 5.065995 -1.124890

H 5.312361 4.181459 -0.960881

H 5.806556 2.976812 0.236828

H 4.718673 2.514918 -1.083871

H 4.705327 5.658335 0.736048

H 3.319058 5.438995 1.802656

H 4.856247 4.634020 2.168031

H -0.310574 3.267566 2.721588

H 0.663580 4.234463 1.606004

H 0.213722 2.564522 1.187823

H 0.971612 0.662433 2.801111

H 2.229570 0.954864 4.013807

H 0.616473 1.657086 4.223534

H 3.135655 3.460091 4.307864

H 2.376496 4.736356 3.341813

H 1.425837 3.837735 4.523977

--------------------------------------------------------

**Cartesian coordinate of 2.** Units are presented in Å.

SCF energy = -3961.72355933 hartree

ZPE = 1.285164 hartree

SCF energy (in toluene) = -3962.57526443 hartree

-------------------------------------------------------

Atom Coordinates (Angstroms)

X Y Z

--------------------------------------------------------

Mo 2.629024 -0.000035 -0.000028

Mo -2.629027 0.000035 0.000015

P -3.154934 2.179589 1.171040

P 3.154958 -2.179594 1.170973

N 4.863725 -0.000032 0.000022

N -4.863728 0.000038 0.000097

P -3.155049 -2.179563 -1.171006

P 3.155023 2.179568 -1.171048

N 0.577239 -0.000019 -0.000051

N -0.577242 0.000013 -0.000041

N 2.605777 1.445669 2.811131

N 2.610769 0.914821 1.803403

N 2.610797 -0.914906 -1.803440

N 2.605787 -1.445822 -2.811136

N -2.605732 -1.445666 2.811175

N -2.610743 -0.914825 1.803444

N -2.610825 0.914909 -1.803396

N -2.605832 1.445815 -2.811096

H -2.491718 3.748886 4.682731

H 2.491792 -3.748908 4.682667

H 3.894636 -2.800256 4.186641

H -3.894590 2.800272 4.186709

C -3.112928 3.475342 3.816265

C 3.112994 -3.475349 3.816199

H 2.306904 -0.854599 3.804618

H -2.306928 0.854561 3.804673

H 3.593399 -4.390207 3.459578

H -3.593307 4.390213 3.459641

H 0.968167 -1.906553 4.284531

H -0.968160 1.906469 4.284601

C -1.566529 1.566721 3.425997

C 1.566530 -1.566781 3.425933

H -0.432032 3.929772 3.227112

H 0.432116 -3.929870 3.227042

C -2.215870 2.795954 2.759536

C 2.215914 -2.795990 2.759469

H 4.486892 -0.943296 2.747389

H -4.486858 0.943290 2.747463

H -0.900928 1.033962 2.739777

H 0.900917 -1.034038 2.739711

H -5.427297 2.371898 2.303832

H 5.427334 -2.371897 2.303740

C -1.074415 3.749281 2.352874

C 1.074493 -3.749356 2.352805

H -3.423247 5.392460 1.607261

H 3.423128 -5.392492 1.607093

H -0.442919 3.313177 1.568763

H 0.442983 -3.313274 1.568693

C -4.791431 1.573754 1.899234

C 4.791459 -1.573755 1.899153

H -7.481016 1.356156 1.674243

H 7.481041 -1.356144 1.674128

H -1.435326 4.724229 2.008126

H 1.435439 -4.724292 2.008058

H -5.033763 4.641296 1.657777

H 5.033666 -4.641383 1.657740

H 4.588987 2.450933 1.392083

H -4.589002 -2.450936 1.392129

C -4.205236 4.928568 0.997324

C 4.205173 -4.928604 0.997223

H 2.822617 5.075012 1.327206

H -2.822619 -5.075014 1.327236

C -6.966512 0.752193 0.929987

C 6.966525 -0.752180 0.929881

H -4.588872 5.706217 0.319111

H 4.588827 -5.706241 0.319007

C -5.570708 0.743171 0.910624

C 5.570720 -0.743164 0.910537

H 5.025269 4.163342 1.497384

H -5.025267 -4.163350 1.497437

H 2.136380 3.445050 1.378143

H -2.136389 -3.445050 1.378177

C 4.832569 3.335011 0.799004

C -4.832580 -3.335018 0.799054

C 2.496165 4.227307 0.705058

C -2.496173 -4.227305 0.705091

H 8.771578 -0.000026 0.000126

H -8.771581 0.000050 0.000253

C -3.686971 3.747240 0.153025

C 3.687001 -3.747228 0.152934

H 5.766475 3.140390 0.258907

H -5.766492 -3.140408 0.258964

C 7.683673 -0.000028 0.000098

C -7.683676 0.000047 0.000211

H 1.652882 4.571976 0.099246

H -1.652891 -4.571968 0.099274

H -1.652850 4.572023 -0.099263

H 1.652865 -4.571928 -0.099511

H -5.766429 3.140361 -0.258877

H 5.766504 -3.140401 -0.258817

C 3.687013 3.747209 -0.153006

C -3.687027 -3.747209 -0.152964

C -2.496146 4.227360 -0.705062

C 2.496212 -4.227273 -0.705244

C -4.832534 3.335027 -0.798969

C 4.832636 -3.335018 -0.798974

H -5.025276 4.163367 -1.497329

H 5.025394 -4.163339 -1.497352

H 4.588849 5.706226 -0.319086

H -4.588857 -5.706229 -0.319048

C -6.966590 -0.752102 -0.929623

C 6.966571 0.752121 -0.929723

H -2.136369 3.445115 -1.378171

H 2.136506 -3.444991 -1.378347

C -5.570785 -0.743094 -0.910372

C 5.570767 0.743106 -0.910455

C 4.205275 4.928544 -0.997296

C -4.205289 -4.928542 -0.997256

H 5.033847 4.641285 -1.657696

H -5.033866 -4.641283 -1.657651

H -2.822617 5.075072 -1.327190

H 2.822691 -5.074975 -1.327382

H 7.481127 1.356078 -1.673948

H -7.481158 -1.356054 -1.673843

H -4.588916 2.450975 -1.392072

H 4.589084 -2.450937 -1.392060

H 1.435318 4.724126 -2.008306

H -1.435341 -4.724105 -2.008288

C 4.791564 1.573688 -1.899118

C -4.791600 -1.573675 -1.899049

H 3.423299 5.392394 -1.607287

H -3.423315 -5.392386 -1.607253

H 5.427478 2.371805 -2.303694

H -5.427522 -2.371789 -2.303618

H 0.443010 3.312995 -1.568961

H -0.443031 -3.312964 -1.568978

C 1.074509 3.749139 -2.353048

C -1.074546 -3.749118 -2.353045

H 4.487015 0.943224 -2.747356

H -4.487060 -0.943210 -2.747290

C 2.216072 2.795907 -2.759623

C -2.216126 -2.795896 -2.759599

H 0.901244 1.033829 -2.740033

H -0.901309 -1.033808 -2.740039

H 0.432157 3.929556 -3.227325

H -0.432215 -3.929537 -3.227337

C 1.566877 1.566646 -3.426174

C -1.566955 -1.566630 -3.426165

H 3.593447 4.390287 -3.459541

H -3.593499 -4.390290 -3.459488

H 3.894917 2.800404 -4.186660

H -3.895000 -2.800412 -4.186602

C 3.113171 3.475395 -3.816250

C -3.113240 -3.475394 -3.816207

H 0.968574 1.906377 -4.284828

H -0.968669 -1.906357 -4.284833

H 2.307361 0.854543 -3.804793

H -2.307453 -0.854533 -3.804766

H 2.492016 3.748930 -4.682757

H -2.492100 -3.748924 -4.682726

--------------------------------------------------------

**Cartesian coordinate of 1a′.** Units are presented in Å.

SCF energy = -2167.06058053 hartree

ZPE = 0.675284 hartree

SCF energy (in toluene) = -2167.54262486 hartree

-------------------------------------------------------

Atom Coordinates (Angstroms)

X Y Z

--------------------------------------------------------

Mo 0.000008 -1.073050 0.000191

P -2.470079 -0.438242 0.064462

P 2.470094 -0.438393 -0.064384

N 0.019104 -1.171615 2.031501

N 0.048727 -1.271385 3.162709

N -0.018951 -1.171987 -2.031106

N -0.048168 -1.272012 -3.162302

N -0.000072 -3.157101 0.000376

N -0.000096 -4.289665 0.000480

N -1.022755 1.862291 0.391879

N 1.022843 1.862217 -0.391845

C -3.783250 -1.235111 1.255506

C -4.447337 -2.438171 0.553735

C -4.865885 -0.268141 1.782084

C -3.021310 -1.785079 2.476323

C -3.375799 -0.004714 -1.603665

C -4.809234 0.543036 -1.451367

C -2.554759 1.088847 -2.327413

C -3.424772 -1.265064 -2.495664

C -2.294466 1.306450 0.834013

C 0.000032 1.026324 0.000056

C -0.659538 3.201011 0.257728

C 0.659693 3.200964 -0.257768

C 2.294507 1.306282 -0.833990

C 3.376059 -0.004780 1.603607

C 3.425113 -1.265032 2.495752

C 2.555072 1.088823 2.327331

C 4.809457 0.543032 1.451161

C 3.782841 -1.235347 -1.255844

C 4.446756 -2.438818 -0.554610

C 3.020389 -1.784778 -2.476588

C 4.865577 -0.268542 -1.782526

H -5.017479 -3.015284 1.296478

H -5.149295 -2.139741 -0.232078

H -3.700803 -3.112470 0.114163

H -5.532326 -0.821645 2.460738

H -4.433772 0.553657 2.367282

H -5.488908 0.163753 0.995706

H -2.265265 -2.520085 2.183507

H -2.525404 -0.997764 3.054289

H -3.738390 -2.281816 3.147108

H -5.514931 -0.207661 -1.081565

H -4.861035 1.420334 -0.793798

H -5.169280 0.862461 -2.440910

H -2.602950 2.053262 -1.806388

H -1.503853 0.820814 -2.452884

H -2.981986 1.240313 -3.329716

H -3.833801 -0.991007 -3.479887

H -2.434989 -1.696824 -2.658862

H -4.071131 -2.044077 -2.079389

H -3.108527 1.984669 0.551447

H -2.299613 1.201627 1.926941

H 3.108615 1.984479 -0.551503

H 2.299601 1.201400 -1.926913

H 2.435363 -1.696840 2.659005

H 4.071527 -2.044054 2.079590

H 3.834117 -0.990825 3.479944

H 2.982302 1.240286 3.329634

H 2.603283 2.053232 1.806299

H 1.504162 0.820802 2.452789

H 5.169489 0.862685 2.440636

H 5.515215 -0.207675 1.081514

H 4.861181 1.420210 0.793425

H 5.016382 -3.015963 -1.297723

H 5.149142 -2.140855 0.230986

H 3.700144 -3.112945 -0.114909

H 2.264142 -2.519564 -2.183727

H 2.524610 -0.997152 -3.054243

H 3.737108 -2.281632 -3.147673

H 4.433502 0.553427 -2.367512

H 5.488877 0.163117 -0.996244

H 5.531739 -0.822094 -2.461415

C -1.329240 4.393727 0.526963

C -0.651923 5.589881 0.258607

C 0.652188 5.589834 -0.258805

C 1.329448 4.393631 -0.527088

H -2.337894 4.398176 0.933860

H -1.148978 6.537098 0.457413

H 1.149288 6.537014 -0.457668

H 2.338101 4.398005 -0.933986

--------------------------------------------------------

**Cartesian coordinate of 1b′.** Units are presented in Å.

SCF energy = -2092.07837395 hartree

ZPE = 0.687596 hartree

SCF energy (in toluene) = -2092.54862968 hartree

-------------------------------------------------------

Atom Coordinates (Angstroms)

X Y Z

--------------------------------------------------------

Mo -0.000003 -0.450716 -0.000001

P -2.559153 -0.097157 0.146579

P 2.559149 -0.097163 -0.146578

N 0.089452 -0.571288 2.020082

N 0.147577 -0.712761 3.149333

N -0.089467 -0.571297 -2.020083

N -0.147561 -0.712778 -3.149334

N -0.000006 -2.491884 0.000003

N -0.000010 -3.629990 0.000004

N -0.710281 2.584302 0.808830

N 0.710286 2.584303 -0.808831

C -3.630430 -1.451854 1.043732

C -3.563961 -2.778649 0.258531

C -5.111039 -1.091857 1.284418

C -2.961563 -1.696488 2.413780

C -3.485752 0.469280 -1.477101

C -4.930262 0.984866 -1.289995

C -2.664073 1.634470 -2.075400

C -3.523831 -0.689808 -2.495773

C -2.894878 1.425189 1.239617

C -1.657575 2.124938 1.824096

C -0.000001 1.727536 -0.000004

C -0.450321 3.916615 0.508849

C 0.450326 3.916615 -0.508848

C 1.657585 2.124941 -1.824093

C 2.894882 1.425185 -1.239612

C 3.485746 0.469277 1.477100

C 3.523870 -0.689823 2.495759

C 2.664038 1.634433 2.075425

C 4.930238 0.984904 1.289979

C 3.630435 -1.451854 -1.043729

C 3.563889 -2.778681 -0.258589

C 2.961640 -1.696411 -2.413826

C 5.111066 -1.091883 -1.284316

H -4.023121 -3.574537 0.864547

H -4.114898 -2.735069 -0.686502

H -2.534235 -3.079670 0.044474

H -5.539389 -1.808656 2.000807

H -5.253650 -0.089393 1.706815

H -5.707929 -1.163436 0.370220

H -1.941049 -2.069567 2.303222

H -2.929932 -0.800889 3.048015

H -3.539571 -2.458011 2.957991

H -5.630229 0.193714 -1.010837

H -5.014790 1.789998 -0.549564

H -5.277681 1.397524 -2.249276

H -2.637603 2.509256 -1.413000

H -1.635321 1.346366 -2.297300

H -3.137872 1.952184 -3.016283

H -3.796791 -0.293521 -3.485375

H -2.560771 -1.196557 -2.598737

H -4.279678 -1.436528 -2.229393

H -3.466436 2.159650 0.662052

H -3.532185 1.128998 2.081444

H -1.130394 1.468351 2.519165

H -1.975711 3.011280 2.388035

H -0.916951 4.732986 1.042814

H 0.916958 4.732987 -1.042810

H 1.130406 1.468360 -2.519170

H 1.975727 3.011285 -2.388025

H 3.466443 2.159641 -0.662042

H 3.532191 1.128992 -2.081436

H 2.560830 -1.196611 2.598717

H 4.279745 -1.436511 2.229369

H 3.796816 -0.293537 3.485364

H 3.137836 1.952148 3.016308

H 2.637534 2.509228 1.413038

H 1.635297 1.346294 2.297331

H 5.277664 1.397554 2.249262

H 5.630221 0.193775 1.010793

H 5.014730 1.790053 0.549561

H 4.023047 -3.574557 -0.864621

H 4.114788 -2.735162 0.686468

H 2.534144 -3.079678 -0.044589

H 1.941116 -2.069484 -2.303342

H 2.930055 -0.800779 -3.048016

H 3.539669 -2.457914 -2.958043

H 5.253725 -0.089412 -1.706680

H 5.707899 -1.163496 -0.370083

H 5.539445 -1.808674 -2.000697

--------------------------------------------------------

**Cartesian coordinate of 2′.** Units are presented in Å.

SCF energy = -2035.59560923 hartree

ZPE = 0.646664 hartree

SCF energy (in toluene) = -2036.04248382 hartree

-------------------------------------------------------

Atom Coordinates (Angstroms)

X Y Z

--------------------------------------------------------

Mo 0.000016 -0.629430 -0.000025

P -2.475140 -0.126805 -0.084486

N -0.000012 1.611016 0.000000

P 2.475149 -0.126775 0.084484

N 0.000033 -3.784578 -0.000094

N 0.000026 -2.647088 -0.000072

N 0.057918 -0.671760 -3.160851

N 0.027181 -0.655488 -2.023986

N -0.027079 -0.655586 2.023938

N -0.057616 -0.671921 3.160807

H -5.438845 -0.775775 -2.533724

H -4.372854 0.629972 -2.502452

C -4.806296 -0.160000 -1.876420

H -2.439319 -0.919292 -3.045223

H -5.463502 0.310218 -1.140837

H -3.595812 -2.254954 -3.046443

C -2.909727 -1.676250 -2.409811

H -4.912278 -2.865135 -1.203021

C -3.718395 -1.059289 -1.251198

H -2.104307 1.264817 -2.020917

H -2.128160 -2.353043 -2.049972

H -3.170680 2.166083 -0.941882

C -4.375088 -2.227245 -0.485879

H -5.550494 0.065966 0.957915

H -3.627850 -2.854240 0.017285

C -2.273161 1.534496 -0.967991

H -1.979345 4.224887 -0.849453

H -5.105379 -1.893725 0.258555

H -4.926929 1.694657 0.613614

H 1.612660 1.273668 -2.396627

C -4.877219 0.851838 1.314881

H 3.901177 -0.552610 -3.448798

C -1.099902 3.708369 -0.471805

H -5.278060 1.206953 2.276394

C -1.081549 2.311331 -0.460587

H 3.127079 1.690194 -3.208396

H 2.458838 -1.245565 -2.692672

C 2.666534 1.504761 -2.226689

C 3.461006 -0.860735 -2.488256

H -0.000053 5.510181 0.000004

C -3.434162 0.354460 1.534353

H 2.727455 2.440309 -1.657806

C -0.000041 4.422117 0.000002

H 4.068201 -1.684164 -2.100737

H -4.068047 -1.684126 2.100876

H -2.727552 2.440396 1.657586

C 3.434187 0.354428 -1.534370

C -3.460893 -0.860635 2.488328

C -2.666581 1.504908 2.226564

H -3.127147 1.690412 3.208247

H 5.278017 1.207017 -2.276465

C 1.099837 3.708391 0.471802

H -2.458699 -1.245392 2.692757

C 1.081514 2.311352 0.460577

C 4.877206 0.851928 -1.314929

H 4.926857 1.694792 -0.613714

H -3.901069 -0.552466 3.448854

H 1.979271 4.224925 0.849447

H -1.612697 1.273896 2.396536

H 5.105697 -1.893371 -0.258263

C 2.273150 1.534541 0.967960

H 5.550545 0.066130 -0.957922

H 3.170657 2.166145 0.941828

H 3.628203 -2.854044 -0.017414

C 4.375281 -2.227043 0.485979

H 2.104321 1.264876 2.020893

C 3.718323 -1.059237 1.251297

H 2.128123 -2.353302 2.049637

H 4.912378 -2.864970 1.203159

C 2.909500 -1.676405 2.409694

H 5.463325 0.310451 1.141440

H 4.372343 0.629980 2.502838

C 4.806001 -0.159895 1.876833

H 3.595535 -2.255036 3.046445

H 2.438823 -0.919560 3.045044

H 5.438465 -0.775666 2.534225

--------------------------------------------------------

**Cartesian coordinate of reactant complex for proton transfer from LutH^+^ to 1a (1a → A-PCP).** Units are presented in Å.

SCF energy =-4551.77561292 hartree

ZPE = 1.501441hartree

SCF energy (in toluene) = -4552.81811202 hartree

-------------------------------------------------------

Atom Coordinates (Angstroms)

X Y Z

--------------------------------------------------------

Mo 2.240784 -0.900529 0.140218

P 2.845497 -1.438317 -2.295039

P 2.886507 -0.714672 2.623231

N 2.565712 1.020684 -0.181607

N 2.718202 2.150598 -0.353898

N 1.695594 -2.865967 0.466056

N 1.339748 -3.927736 0.623697

N 0.157072 -0.533339 0.079130

N -0.980307 -0.367017 0.034574

N 5.179612 -1.300881 -0.872411

N 5.055993 -1.704382 1.275656

C 2.283943 -0.374474 -3.820088

C 0.965563 -0.935469 -4.392715

C 3.331855 -0.247107 -4.947762

C 1.988574 1.043297 -3.296324

C 2.936270 -3.295057 -2.862760

C 3.503706 -3.515970 -4.279521

C 3.865452 -4.051207 -1.883690

C 1.523622 -3.917762 -2.802909

C 4.706189 -1.005591 -2.216566

C 4.298897 -1.334029 0.187025

C 6.470669 -1.641798 -0.467277

C 6.389060 -1.910475 0.919207

C 4.450722 -1.813225 2.594536

C 3.640024 0.969817 3.236098

C 2.558590 2.064533 3.108727

C 4.833970 1.329603 2.320565

C 4.173771 0.975266 4.683255

C 1.922364 -1.507571 4.111078

C 0.872535 -0.508808 4.640736

C 1.156828 -2.727298 3.563627

C 2.812919 -2.002785 5.271689

H 0.551947 -0.213076 -5.111131

H 1.103492 -1.879627 -4.929767

H 0.209063 -1.085062 -3.612237

H 2.918859 0.394410 -5.740423

H 4.257375 0.231464 -4.601532

H 3.596204 -1.201004 -5.409212

H 1.216678 1.036551 -2.520461

H 2.880659 1.525883 -2.880258

H 1.631487 1.663881 -4.131937

H 2.843512 -3.135489 -5.064798

H 4.498475 -3.072586 -4.415797

H 3.611829 -4.597607 -4.446810

H 4.915024 -3.752252 -1.996446

H 3.585005 -3.916944 -0.836908

H 3.809476 -5.126063 -2.108420

H 1.601312 -4.996396 -3.004007

H 1.054120 -3.797751 -1.824489

H 0.851482 -3.493235 -3.554584

H 5.304979 -1.563515 -2.945954

H 4.819418 0.065258 -2.432131

H 5.182538 -1.526370 3.359165

H 4.148131 -2.851015 2.787298

H 2.155763 2.127640 2.094449

H 1.721346 1.903261 3.794557

H 3.006252 3.037869 3.367227

H 5.173634 2.345697 2.573692

H 5.688778 0.659734 2.475628

H 4.576367 1.310998 1.259515

H 4.672387 1.938458 4.869592

H 3.380419 0.876500 5.430263

H 4.919711 0.191212 4.863161

H 0.206234 -1.030623 5.342211

H 1.319000 0.329744 5.185824

H 0.244273 -0.109330 3.834187

H 0.450093 -2.442887 2.778309

H 1.822602 -3.501802 3.166247

H 0.583500 -3.181176 4.385146

H 3.514104 -2.784598 4.953017

H 3.384215 -1.208675 5.756590

H 2.166565 -2.453322 6.039149

Mo -3.033395 -0.043391 -0.011487

P -4.074970 -2.404468 0.030630

P -3.285487 2.504005 -0.088846

N -2.920466 -0.026099 2.017772

N -2.768907 -0.016880 3.143605

N -2.867513 -0.115000 -2.036378

N -2.692908 -0.183262 -3.156782

N -6.116299 -0.604712 0.312755

N -5.788317 1.443032 -0.377377

C -3.551774 -3.822072 1.248554

C -2.432777 -4.670532 0.611817

C -4.705595 -4.741956 1.707952

C -2.967616 -3.149887 2.505796

C -4.558979 -3.215760 -1.665261

C -5.351274 -4.535046 -1.562016

C -5.457347 -2.214292 -2.430385

C -3.273666 -3.479070 -2.482251

C -5.790313 -1.959741 0.728814

C -5.125713 0.287104 -0.029679

C -5.052997 2.625758 -0.792188

C -3.493899 3.458700 1.594931

C -2.199253 3.300108 2.422447

C -4.669980 2.810114 2.362878

C -3.816606 4.961614 1.473694

C -2.358149 3.687029 -1.327793

C -1.023794 4.147406 -0.706539

C -2.018501 2.862331 -2.583274

C -3.168152 4.919399 -1.787797

H -2.006026 -5.332758 1.379011

H -2.796509 -5.308912 -0.199757

H -1.614057 -4.050223 0.225454

H -4.299603 -5.487672 2.407464

H -5.483262 -4.191134 2.251638

H -5.182067 -5.290125 0.892302

H -2.088336 -2.543057 2.269445

H -3.694047 -2.509587 3.019139

H -2.661018 -3.932544 3.215592

H -4.755993 -5.359443 -1.156996

H -6.263354 -4.435664 -0.959567

H -5.666348 -4.832607 -2.573125

H -6.442236 -2.104972 -1.959497

H -5.011867 -1.221986 -2.523767

H -5.626380 -2.601842 -3.445530

H -3.552401 -3.766068 -3.507018

H -2.631856 -2.597467 -2.547826

H -2.681797 -4.299082 -2.063227

H -6.573495 -2.645101 0.384408

H -5.745933 -2.018541 1.824377

H -5.578299 3.524753 -0.448080

H -4.991779 2.666934 -1.887773

H -1.896665 2.255759 2.526023

H -1.361061 3.854064 1.985715

H -2.366698 3.701689 3.433094

H -4.716054 3.250357 3.369706

H -5.635249 3.007615 1.880276

H -4.560270 1.730710 2.478781

H -4.023601 5.357267 2.479161

H -2.984709 5.544980 1.065596

H -4.708214 5.157312 0.864377

H -0.430721 4.656640 -1.482279

H -1.167278 4.857270 0.115519

H -0.432533 3.298276 -0.338355

H -1.366890 2.015665 -2.349299

H -2.910391 2.473555 -3.086957

H -1.493689 3.508994 -3.302835

H -4.087905 4.636993 -2.314388

H -3.436147 5.598099 -0.975282

H -2.559961 5.492052 -2.504405

C 7.680473 -1.742666 -1.153354

C 8.807793 -2.127898 -0.417916

C 8.725624 -2.399119 0.956805

C 7.512859 -2.297010 1.648573

H 7.749906 -1.532371 -2.218253

H 9.765892 -2.220139 -0.924629

H 9.620676 -2.699427 1.496994

H 7.452754 -2.518518 2.711611

C -9.528636 1.655868 -0.289316

C -8.239024 2.149711 -0.518287

C -7.170667 1.289527 -0.268410

C -7.382132 -0.031264 0.187829

C -8.667412 -0.519877 0.418904

C -9.738603 0.346311 0.170577

H -10.384585 2.301162 -0.474158

H -8.081292 3.162564 -0.881519

H -8.835594 -1.531609 0.780413

H -10.754327 -0.004508 0.339146

C 4.960340 4.879062 -1.541666

N 3.904414 4.765279 -0.680073

C 3.418014 5.796434 0.075226

C 4.029273 7.037566 -0.042707

C 5.112904 7.204847 -0.913265

C 5.576578 6.116295 -1.663019

H 6.416288 6.223150 -2.343707

H 5.594020 8.175355 -1.005781

H 3.653205 7.867874 0.548091

C 5.366326 3.649092 -2.295572

H 6.271435 3.838682 -2.879848

H 5.559398 2.813597 -1.608842

H 4.570727 3.329547 -2.982922

C 2.255600 5.500958 0.971577

H 1.964902 6.395610 1.529770

H 1.390201 5.152458 0.391290

H 2.502242 4.705089 1.687055

H 3.466929 3.831743 -0.568743

--------------------------------------------------------

**Cartesian coordinate of transition state for proton transfer from LutH^+^ to 1a (1a → A-PCP).** Units are presented in Å.

SCF energy = -4551.76116923 hartree

ZPE = 1.495490 hartree

SCF energy (in toluene) = -4552.79886969 hartree

Imaginary frequency: 876i cm^-1^

-------------------------------------------------------

Atom Coordinates (Angstroms)

X Y Z

--------------------------------------------------------

Mo 2.227379 -0.755491 0.053104

P 2.812184 -1.316285 -2.401078

P 2.870335 -0.605808 2.542541

N 2.485284 1.096191 -0.268459

N 2.447993 2.266344 -0.467646

N 1.593377 -2.755649 0.368236

N 1.176946 -3.790231 0.516335

N 0.104403 -0.446652 0.068762

N -1.038659 -0.312825 0.070532

N 5.150182 -1.274211 -0.969236

N 5.020359 -1.649212 1.182750

C 2.301016 -0.236219 -3.929698

C 0.964466 -0.746531 -4.507141

C 3.361094 -0.159066 -5.050593

C 2.062042 1.192921 -3.410015

C 2.816245 -3.178754 -2.957076

C 3.381827 -3.435553 -4.368996

C 3.703870 -3.976611 -1.972113

C 1.372051 -3.726635 -2.907518

C 4.687764 -0.968358 -2.316541

C 4.279368 -1.262564 0.093495

C 6.430656 -1.654912 -0.564561

C 6.344733 -1.907252 0.823792

C 4.422943 -1.723507 2.509029

C 3.626086 1.074473 3.143193

C 2.539974 2.165966 3.023101

C 4.802351 1.430462 2.206432

C 4.178188 1.081755 4.583136

C 1.907558 -1.398735 4.029956

C 0.861444 -0.399019 4.565427

C 1.140392 -2.617745 3.483733

C 2.802620 -1.893622 5.187515

H 0.587852 -0.010141 -5.231497

H 1.067141 -1.696928 -5.040632

H 0.195603 -0.861856 -3.732951

H 2.976078 0.492148 -5.848906

H 4.301124 0.289221 -4.703383

H 3.592012 -1.125185 -5.504792

H 1.280935 1.221410 -2.645172

H 2.967339 1.641546 -2.984619

H 1.744574 1.827563 -4.250660

H 2.752373 -3.019237 -5.160840

H 4.402300 -3.052209 -4.497123

H 3.428113 -4.522092 -4.532848

H 4.768692 -3.743625 -2.096873

H 3.443375 -3.813007 -0.924461

H 3.582334 -5.049066 -2.180977

H 1.397271 -4.815371 -3.060711

H 0.880063 -3.537487 -1.951188

H 0.742853 -3.301460 -3.694749

H 5.262727 -1.557112 -3.040413

H 4.850031 0.094495 -2.535987

H 5.165649 -1.430394 3.260570

H 4.114618 -2.754853 2.724360

H 2.122485 2.220875 2.013712

H 1.713071 2.007882 3.721776

H 2.988593 3.141656 3.266154

H 5.144733 2.448735 2.441930

H 5.660541 0.760805 2.346125

H 4.515253 1.401672 1.152608

H 4.677547 2.045757 4.760834

H 3.393588 0.984383 5.339604

H 4.927221 0.298705 4.755676

H 0.197890 -0.921791 5.268668

H 1.313040 0.435932 5.111429

H 0.230087 0.006256 3.764453

H 0.422313 -2.329786 2.710299

H 1.804620 -3.388502 3.074951

H 0.578389 -3.079588 4.308322

H 3.496208 -2.682995 4.870829

H 3.382141 -1.100243 5.663591

H 2.157378 -2.334056 5.961512

Mo -3.070536 -0.015181 0.023161

P -4.138352 -2.345194 0.352142

P -3.314187 2.519047 -0.336364

N -2.962712 0.224202 2.037718

N -2.818960 0.365580 3.156036

N -2.920003 -0.301093 -1.985743

N -2.758468 -0.489700 -3.094271

N -6.164295 -0.506150 0.387911

N -5.808118 1.424311 -0.571143

C -3.640509 -3.610618 1.739997

C -2.548415 -4.565214 1.216767

C -4.813720 -4.433282 2.316136

C -3.027881 -2.799090 2.896471

C -4.641235 -3.359168 -1.228311

C -5.436869 -4.652753 -0.958379

C -5.536470 -2.456252 -2.108754

C -3.361788 -3.723164 -2.013935

C -5.845921 -1.789715 0.994248

C -5.164276 0.324563 -0.056503

C -5.048531 2.531947 -1.129157

C -3.608892 3.653705 1.213058

C -2.355800 3.610627 2.116275

C -4.813530 3.083599 1.998268

C -3.933974 5.128365 0.899486

C -2.324016 3.551166 -1.654491

C -1.035852 4.118198 -1.022809

C -1.898643 2.574679 -2.766778

C -3.123950 4.695068 -2.315528

H -2.142183 -5.138743 2.062615

H -2.928870 -5.290051 0.489920

H -1.712766 -4.021524 0.758260

H -4.424889 -5.089025 3.109312

H -5.578687 -3.798943 2.780759

H -5.302286 -5.073309 1.578225

H -2.154203 -2.228447 2.567712

H -3.739802 -2.098556 3.346991

H -2.707673 -3.493564 3.687586

H -4.842282 -5.423650 -0.458186

H -6.346353 -4.479273 -0.369004

H -5.758545 -5.071606 -1.923402

H -6.525484 -2.296803 -1.661440

H -5.090680 -1.480071 -2.308548

H -5.694894 -2.956059 -3.075355

H -3.648868 -4.196747 -2.964650

H -2.756718 -2.844858 -2.250638

H -2.731367 -4.434928 -1.471480

H -6.637668 -2.511277 0.761665

H -5.788046 -1.683790 2.085779

H -5.588567 3.469761 -0.954714

H -4.932793 2.400842 -2.213075

H -2.086981 2.592158 2.405379

H -1.485112 4.071085 1.637981

H -2.561214 4.174923 3.038170

H -4.897732 3.624207 2.952275

H -5.759729 3.225779 1.461674

H -4.707425 2.021131 2.226469

H -4.195764 5.635471 1.839959

H -3.085276 5.669897 0.469310

H -4.793923 5.242413 0.227587

H -0.414228 4.548846 -1.822753

H -1.236260 4.917258 -0.301375

H -0.440624 3.338863 -0.529834

H -1.282556 1.760891 -2.372476

H -2.753511 2.130641 -3.288700

H -1.307443 3.123438 -3.515154

H -4.001859 4.325808 -2.859798

H -3.455504 5.461549 -1.611449

H -2.479111 5.189938 -3.057144

C 7.633651 -1.805601 -1.255125

C 8.746851 -2.225389 -0.519832

C 8.659896 -2.481484 0.858917

C 7.456022 -2.328851 1.554166

H 7.706796 -1.607758 -2.321994

H 9.699247 -2.357910 -1.028149

H 9.545796 -2.809697 1.397744

H 7.391921 -2.538095 2.619343

C -9.546740 1.681666 -0.554755

C -8.250261 2.126824 -0.836861

C -7.193424 1.298766 -0.460529

C -7.423561 0.054724 0.170350

C -8.716154 -0.385016 0.453491

C -9.775176 0.449709 0.078764

H -10.393973 2.304001 -0.834165

H -8.078699 3.078997 -1.333578

H -8.898360 -1.335574 0.949330

H -10.796148 0.136014 0.284590

C 5.492432 4.038104 -0.851406

N 4.230211 4.163098 -0.383947

C 3.796332 5.319282 0.169264

C 4.660761 6.412200 0.283848

C 5.970833 6.300341 -0.178380

C 6.391914 5.102223 -0.754986

H 7.404250 4.987138 -1.133471

H 6.656883 7.140864 -0.095088

H 4.300501 7.336021 0.728905

C 5.869623 2.729504 -1.493654

H 6.930552 2.718066 -1.763229

H 5.674833 1.884282 -0.822043

H 5.282581 2.569700 -2.408659

C 2.367707 5.376617 0.639806

H 1.676484 5.113149 -0.170169

H 2.192589 4.661904 1.453772

H 2.119643 6.379749 1.000790

H 3.332207 3.086586 -0.419738

--------------------------------------------------------

**Cartesian coordinate of product complex for proton transfer from LutH^+^ to 1a (1a → A-PCP).** Units are presented in Å.

SCF energy = -4551.76532343 hartree

ZPE = 1.499087 hartree

SCF energy (in toluene) = -4552.80284930 hartree

-------------------------------------------------------

Atom Coordinates (Angstroms)

X Y Z

--------------------------------------------------------

Mo 2.143088 -0.705313 0.127746

P 2.691437 -1.504645 -2.280955

P 2.796365 -0.380337 2.592625

N 2.552393 1.050691 -0.320640

N 2.580415 2.233512 -0.592493

N 1.376424 -2.668664 0.627961

N 0.889706 -3.649321 0.874159

N 0.049478 -0.357975 0.151378

N -1.101202 -0.266776 0.175425

N 5.003277 -1.626975 -0.796236

N 4.803954 -1.820043 1.374748

C 2.338073 -0.477972 -3.886423

C 0.946332 -0.849826 -4.436939

C 3.403548 -0.627305 -4.994372

C 2.294724 1.005688 -3.475786

C 2.482434 -3.388458 -2.705108

C 3.002178 -3.806753 -4.096377

C 3.283086 -4.216306 -1.671873

C 0.983803 -3.752921 -2.611053

C 4.593172 -1.363853 -2.171641

C 4.135456 -1.439321 0.243731

C 6.229566 -2.111071 -0.336369

C 6.097144 -2.247593 1.064482

C 4.189190 -1.690742 2.692281

C 3.765845 1.236486 3.041538

C 2.841244 2.455220 2.825313

C 4.972787 1.352086 2.082748

C 4.318678 1.282268 4.480415

C 1.713974 -0.896295 4.120405

C 0.814832 0.284772 4.542047

C 0.787837 -2.040382 3.663950

C 2.517973 -1.404088 5.337850

H 0.671644 -0.130590 -5.221676

H 0.921926 -1.845924 -4.890471

H 0.168960 -0.799922 -3.664096

H 3.118585 0.018472 -5.837777

H 4.395731 -0.293963 -4.664188

H 3.495969 -1.643493 -5.382670

H 1.507175 1.206191 -2.744825

H 3.244189 1.354864 -3.052595

H 2.093620 1.612811 -4.370679

H 2.413840 -3.384194 -4.916244

H 4.056460 -3.544227 -4.252062

H 2.929097 -4.900990 -4.179770

H 4.365989 -4.096405 -1.799599

H 3.036826 -3.977375 -0.635534

H 3.057250 -5.280967 -1.826916

H 0.872680 -4.844851 -2.680150

H 0.530752 -3.428488 -1.671030

H 0.402444 -3.312180 -3.426699

H 5.109192 -2.060895 -2.841539

H 4.878577 -0.343213 -2.454523

H 4.959019 -1.426314 3.426378

H 3.748645 -2.648772 2.995662

H 2.445449 2.502193 1.807090

H 1.997229 2.468637 3.521042

H 3.424023 3.371719 3.003564

H 5.450857 2.329911 2.236982

H 5.734262 0.586557 2.279018

H 4.679496 1.286299 1.032045

H 4.941214 2.182796 4.585464

H 3.529538 1.349219 5.235630

H 4.958152 0.423077 4.720848

H 0.073946 -0.075342 5.269760

H 1.375167 1.092298 5.024829

H 0.257560 0.702095 3.693725

H 0.144725 -1.736912 2.831371

H 1.342963 -2.939433 3.370692

H 0.137664 -2.323038 4.504144

H 3.112225 -2.296278 5.102853

H 3.184925 -0.652485 5.765795

H 1.808744 -1.695959 6.125837

Mo -3.113429 0.036953 0.082540

P -4.280626 -2.275710 0.229025

P -3.249416 2.597945 -0.161752

N -3.103280 0.183955 2.112395

N -3.020734 0.267112 3.241461

N -2.880370 -0.163608 -1.928235

N -2.670900 -0.303044 -3.035307

N -6.244145 -0.364319 0.224524

N -5.762894 1.605542 -0.588124

C -3.933108 -3.617063 1.590075

C -2.830367 -4.582315 1.110457

C -5.174089 -4.427316 2.029562

C -3.395460 -2.881851 2.832897

C -4.692236 -3.193561 -1.431295

C -5.540526 -4.472662 -1.283323

C -5.500351 -2.224295 -2.326537

C -3.367018 -3.557335 -2.137020

C -6.013231 -1.694040 0.766962

C -5.191972 0.456346 -0.098956

C -4.934869 2.713682 -1.040749

C -3.580391 3.672653 1.422782

C -2.380784 3.544382 2.388293

C -4.847373 3.117319 2.114461

C -3.834199 5.170369 1.155306

C -2.137390 3.631575 -1.370861

C -0.866111 4.102580 -0.635099

C -1.691366 2.692546 -2.507473

C -2.846300 4.841988 -2.018763

H -2.511805 -5.207885 1.956900

H -3.171406 -5.259023 0.320168

H -1.944210 -4.045840 0.748092

H -4.869213 -5.128224 2.820637

H -5.957162 -3.790125 2.459059

H -5.618159 -5.020461 1.227059

H -2.466416 -2.345569 2.616692

H -4.113494 -2.164209 3.245596

H -3.186248 -3.622645 3.618892

H -5.004332 -5.284723 -0.782642

H -6.482377 -4.296129 -0.748645

H -5.805557 -4.833574 -2.288029

H -6.508872 -2.046908 -1.932561

H -5.012885 -1.257578 -2.463691

H -5.617227 -2.682267 -3.319376

H -3.590449 -3.953449 -3.138710

H -2.711817 -2.691528 -2.260013

H -2.811527 -4.330311 -1.595807

H -6.802507 -2.371852 0.421685

H -6.048262 -1.662829 1.863672

H -5.450110 3.660468 -0.841785

H -4.764751 2.637105 -2.122559

H -2.136397 2.504050 2.614281

H -1.480511 4.028711 1.996946

H -2.633551 4.039509 3.337607

H -4.971188 3.628812 3.079993

H -5.754442 3.310434 1.528883

H -4.791321 2.045200 2.312809

H -4.130127 5.648304 2.100732

H -2.943519 5.695519 0.796087

H -4.650127 5.343747 0.442248

H -0.166782 4.522042 -1.373168

H -1.069706 4.886672 0.101526

H -0.347129 3.274727 -0.136120

H -1.127667 1.835517 -2.126881

H -2.533442 2.314308 -3.097966

H -1.038184 3.254266 -3.191534

H -3.703632 4.540217 -2.633470

H -3.187944 5.586668 -1.296733

H -2.135488 5.342205 -2.692961

C 7.418855 -2.441586 -0.988206

C 8.467364 -2.921479 -0.199398

C 8.333615 -3.062353 1.193605

C 7.145509 -2.730237 1.849358

H 7.529026 -2.330855 -2.064291

H 9.407541 -3.192396 -0.674275

H 9.171995 -3.440916 1.773706

H 7.044440 -2.849096 2.925432

C -9.484253 1.989102 -0.812373

C -8.158707 2.406102 -0.976549

C -7.156400 1.521443 -0.580252

C -7.467842 0.251592 -0.044229

C -8.789523 -0.160698 0.121741

C -9.793803 0.730040 -0.273217

H -10.290652 2.655456 -1.110274

H -7.924805 3.380061 -1.399886

H -9.034649 -1.132246 0.544390

H -10.835666 0.439699 -0.158044

C 6.179759 3.687138 -1.310499

N 4.950823 3.983513 -0.842911

C 4.662853 5.251368 -0.482106

C 5.617270 6.272895 -0.573154

C 6.892448 5.970524 -1.046083

C 7.180308 4.659155 -1.422260

H 8.162702 4.387981 -1.801699

H 7.651328 6.746861 -1.124612

H 5.355984 7.285992 -0.276573

C 6.424974 2.257385 -1.726142

H 7.464892 2.101713 -2.033546

H 6.200038 1.566187 -0.903260

H 5.775476 1.989853 -2.571766

C 3.270344 5.521985 0.030344

H 3.101109 6.594990 0.171247

H 2.514721 5.133655 -0.663545

H 3.105440 5.020314 0.993591

H 3.516582 2.727501 -0.656791

--------------------------------------------------------

**Cartesian coordinate of A-PCP.** Units are presented in Å.

SCF energy = -4225.05566529 hartree

ZPE = 1.354337 hartree

SCF energy (in toluene) = -4226.00441321 hartree

-------------------------------------------------------

Atom Coordinates (Angstroms)

X Y Z

--------------------------------------------------------

C 8.022218 0.744584 1.479419

C 6.877470 0.373297 0.775014

C 6.960982 -0.370361 -0.423351

C 8.192250 -0.751108 -0.955806

C 9.339715 -0.374948 -0.249273

C 9.256347 0.358252 0.945702

N 5.516368 0.587859 1.002296

C 4.752537 0.006905 0.021496

N 5.644383 -0.576112 -0.841821

Mo 2.622389 -0.000066 -0.106820

N 2.443224 -1.300451 1.446531

N 2.263168 -2.031555 2.295501

C 5.179432 -1.304673 -2.012708

P 3.411288 -1.924646 -1.674701

C 2.773536 -2.196177 -3.487795

C 3.846806 -2.707012 -4.474424

C 4.895318 1.316129 2.099950

P 3.187631 1.921934 1.521320

C 3.630515 3.663625 0.787699

C 4.770102 3.473875 -0.240930

N 0.595312 0.070433 -0.080992

N -0.549996 0.157613 0.060286

Mo -2.650456 0.159982 0.178927

N -2.261937 -1.299300 -1.417551

N -1.948891 -2.024904 -2.212002

N -2.776950 1.397789 1.534087

N -2.646148 2.243328 2.419294

C -4.744399 -0.133672 -0.099349

N -5.602292 -0.742195 0.769986

C -6.907792 -0.713377 0.273778

C -6.836015 -0.069116 -0.982301

N -5.493510 0.274003 -1.165369

C -7.973585 0.117836 -1.769528

C -9.186191 -0.348680 -1.257641

C -9.258953 -0.984222 -0.004364

C -8.122311 -1.175787 0.783915

C -5.119959 -1.328887 2.017144

P -3.266535 -1.753040 1.823824

C -2.708339 -1.820843 3.677515

C -3.758179 -2.414836 4.642938

C -4.899079 1.040805 -2.257062

P -3.306077 1.891928 -1.617964

C -4.006753 3.576329 -0.969468

C -5.155313 3.267495 0.019671

C -3.372850 -3.571553 1.148078

C -4.333342 -3.592081 -0.064773

C -1.964206 -4.017114 0.696198

C -3.913498 -4.610650 2.152916

C -2.366752 2.197977 -3.284004

C -1.645214 0.884883 -3.644813

C -3.270703 2.582438 -4.476097

C -1.286243 3.279229 -3.073871

N 2.576601 1.288114 -1.680660

N 2.483972 2.010400 -2.551009

C 3.765077 -3.663716 -0.887354

C 4.755587 -3.466815 0.285230

C 2.441150 -4.245894 -0.343112

C 4.401970 -4.697570 -1.838066

C 2.283784 2.185360 3.215786

C 1.735052 0.817841 3.665066

C 3.189423 2.718251 4.348346

C 1.075796 3.124165 3.018587

C -2.429931 -0.374548 4.127363

C -1.385669 -2.607325 3.788093

C -4.584096 4.496785 -2.064034

C -2.886845 4.336410 -0.223312

C 2.274620 -0.833648 -4.004766

C 1.570713 -3.161568 -3.484784

C 4.122293 4.694318 1.824802

C 2.395681 4.246609 0.064592

H -0.984397 -2.481558 4.803804

H -1.514912 -3.682225 3.625670

H -0.623189 -2.239516 3.089651

H -3.348850 -2.375245 5.662753

H -4.688135 -1.832231 4.656074

H -4.008462 -3.457029 4.436232

H -1.647128 0.100453 3.530861

H -3.322577 0.260191 4.080377

H -2.098261 -0.392234 5.175850

H -3.232069 -4.789443 2.990044

H -4.897997 -4.340666 2.555743

H -4.035125 -5.569514 1.628298

H -5.377895 -3.442231 0.234851

H -4.090948 -2.847011 -0.825312

H -4.274098 -4.580926 -0.541078

H -2.036819 -5.004442 0.217221

H -1.515337 -3.330131 -0.025223

H -1.272061 -4.111560 1.538381

H -5.729525 -2.205743 2.261820

H -5.222421 -0.602404 2.832908

H -5.636075 1.760935 -2.631255

H -4.633239 0.370141 -3.083883

H -2.447978 3.747189 0.586099

H -2.081441 4.651048 -0.893317

H -3.315560 5.248209 0.218759

H -5.455487 4.204085 0.511130

H -6.044664 2.873532 -0.487980

H -4.863614 2.551677 0.792977

H -5.060389 5.360303 -1.577623

H -3.813335 4.891082 -2.733071

H -5.355484 4.006956 -2.671819

H -0.627036 3.299379 -3.952951

H -1.709968 4.282820 -2.963046

H -0.656375 3.066034 -2.200946

H -0.948547 0.577736 -2.858250

H -2.342604 0.061260 -3.840901

H -1.065491 1.041610 -4.565439

H -4.010622 1.805607 -4.707298

H -3.798949 3.528395 -4.336784

H -2.638660 2.692043 -5.369211

H 1.103163 -3.148745 -4.479859

H 1.857223 -4.197552 -3.276776

H 0.804463 -2.860720 -2.758660

H 3.386526 -2.810092 -5.468103

H 4.679107 -2.001088 -4.585788

H 4.258486 -3.682006 -4.205499

H 1.464066 -0.439096 -3.384160

H 3.069290 -0.080281 -4.045217

H 1.892230 -0.960929 -5.028370

H 3.731311 -5.004913 -2.646006

H 5.342489 -4.345622 -2.281287

H 4.642255 -5.600489 -1.257919

H 5.759270 -3.196455 -0.065531

H 4.429838 -2.711021 1.002054

H 4.849663 -4.419437 0.826400

H 2.660496 -5.164271 0.221041

H 1.929226 -3.556884 0.333095

H 1.746740 -4.515274 -1.145611

H 5.873186 -2.124723 -2.230338

H 5.155734 -0.638504 -2.885040

H 5.550086 2.138601 2.409850

H 4.752969 0.650718 2.961506

H 2.015946 3.581414 -0.714142

H 1.576159 4.469093 0.754647

H 2.683164 5.191704 -0.419622

H 4.937304 4.431039 -0.755298

H 5.717297 3.194425 0.236690

H 4.536247 2.725777 -1.001574

H 4.460427 5.593557 1.289409

H 3.334757 5.009768 2.516261

H 4.976425 4.332844 2.411412

H 0.461023 3.109651 3.929857

H 1.370425 4.164226 2.844202

H 0.430769 2.797682 2.193203

H 1.032053 0.404961 2.935237

H 2.526087 0.079259 3.836933

H 1.199249 0.950969 4.616377

H 4.011979 2.029534 4.579183

H 3.614421 3.703453 4.144841

H 2.585260 2.807387 5.263066

H -8.185735 -1.661679 1.754486

H -10.222117 -1.333898 0.359881

H -10.093942 -0.218829 -1.842206

H -7.921828 0.602252 -2.741579

H 10.169660 0.631537 1.469256

H 7.962640 1.307271 2.408014

H 8.263244 -1.313757 -1.883708

H 10.316578 -0.656337 -0.635950

H -3.541418 2.691382 2.693648

--------------------------------------------------------

**Cartesian coordinate of transition state for N_2_ elimination from A-PCP (A-PCP → B-PCP).** Units are presented in Å.

SCF energy = -4225.04685059 hartree

ZPE = 1.354108 hartree

SCF energy (in toluene) = -4225.99776101 hartree

Imaginary frequency: 73i cm^-1^

--------------------------------------------------------

Atom Coordinates (Angstroms)

X Y Z

--------------------------------------------------------

C 7.889583 1.169565 1.410191

C 6.775411 0.654594 0.748514

C 6.913913 -0.217093 -0.353978

C 8.170029 -0.587551 -0.832872

C 9.286794 -0.067065 -0.170105

C 9.149140 0.794402 0.930527

N 5.401711 0.816674 0.942086

C 4.682607 0.085717 0.031601

N 5.615330 -0.543773 -0.752112

Mo 2.550885 -0.046577 -0.122721

N 2.418414 -1.167725 1.568129

N 2.281797 -1.806091 2.495510

C 5.210584 -1.427653 -1.834868

P 3.477626 -2.107411 -1.446418

C 2.881779 -2.621354 -3.219512

C 3.995593 -3.180669 -4.132955

C 4.730789 1.632151 1.944467

P 3.003568 2.074343 1.289524

C 3.366797 3.733390 0.351414

C 4.528757 3.482383 -0.639524

N 0.538337 -0.033872 -0.127008

N -0.621107 0.041255 -0.016419

Mo -2.642246 0.249734 0.301353

N -2.151207 -1.589736 -2.091386

N -1.572227 -2.230220 -2.787622

N -2.833395 1.402707 1.667654

N -2.702933 2.229929 2.588948

C -4.691964 -0.191001 -0.095002

N -5.534005 -0.933860 0.683052

C -6.809660 -0.983468 0.120004

C -6.735679 -0.249911 -1.086049

N -5.423848 0.221172 -1.173524

C -7.846205 -0.108738 -1.920721

C -9.034749 -0.712755 -1.506446

C -9.110429 -1.437526 -0.302165

C -8.000976 -1.584424 0.532398

C -5.064460 -1.566834 1.912379

P -3.166334 -1.765545 1.815513

C -2.721925 -1.877332 3.694360

C -3.716125 -2.699646 4.543401

C -4.854719 1.099018 -2.192891

P -3.327967 1.996992 -1.464037

C -4.129268 3.612955 -0.754733

C -5.275673 3.202717 0.199487

C -2.996265 -3.531060 1.030362

C -3.923533 -3.603820 -0.204674

C -1.525118 -3.702883 0.590781

C -3.384134 -4.714603 1.939293

C -2.368472 2.430637 -3.092602

C -1.600225 1.161619 -3.508227

C -3.253712 2.852995 -4.285544

C -1.332824 3.532855 -2.790997

N 2.495807 1.039785 -1.840590

N 2.406620 1.643538 -2.796766

C 3.915347 -3.723230 -0.460093

C 4.879740 -3.344691 0.688350

C 2.619913 -4.311155 0.141712

C 4.616722 -4.819150 -1.289015

C 2.060581 2.482852 2.930211

C 1.574735 1.150035 3.530771

C 2.922894 3.192442 3.997604

C 0.811524 3.331390 2.614371

C -2.710418 -0.440176 4.248536

C -1.296644 -2.447242 3.842620

C -4.732900 4.552747 -1.816960

C -3.059025 4.390374 0.044055

C 2.308381 -1.362861 -3.897587

C 1.739420 -3.650399 -3.105637

C 3.791916 4.907926 1.255949

C 2.109827 4.152971 -0.442404

H -0.981973 -2.349438 4.891285

H -1.233374 -3.509823 3.584995

H -0.569665 -1.898615 3.230769

H -3.392865 -2.653419 5.593395

H -4.732585 -2.286981 4.509933

H -3.764995 -3.754391 4.267008

H -1.968212 0.189197 3.753218

H -3.684899 0.056534 4.157380

H -2.467261 -0.480526 5.320465

H -2.711938 -4.834349 2.793958

H -4.413875 -4.641378 2.311998

H -3.322418 -5.641049 1.349427

H -4.981906 -3.652503 0.080102

H -3.800055 -2.761177 -0.888857

H -3.698956 -4.523043 -0.764700

H -1.427866 -4.616052 -0.014692

H -1.166643 -2.856742 -0.003880

H -0.851885 -3.805292 1.448723

H -5.583095 -2.523141 2.046170

H -5.301294 -0.926671 2.771169

H -5.627355 1.798128 -2.533969

H -4.532086 0.506212 -3.057925

H -2.598532 3.782915 0.828000

H -2.266580 4.782611 -0.600611

H -3.540290 5.253765 0.527657

H -5.642977 4.104226 0.710754

H -6.129097 2.771493 -0.339437

H -4.955594 2.484640 0.959391

H -5.262117 5.366927 -1.301094

H -3.969829 5.017799 -2.448399

H -5.466419 4.055890 -2.464938

H -0.641772 3.616737 -3.641515

H -1.795874 4.515122 -2.649435

H -0.732439 3.299901 -1.902325

H -0.935555 0.806204 -2.713951

H -2.278368 0.348341 -3.793655

H -0.982022 1.389829 -4.388248

H -3.962769 2.067747 -4.577884

H -3.816247 3.771868 -4.107152

H -2.604718 3.031434 -5.155273

H 1.278107 -3.782572 -4.094890

H 2.086125 -4.634788 -2.775828

H 0.952879 -3.311037 -2.420251

H 3.553498 -3.425643 -5.109843

H 4.786647 -2.444624 -4.322782

H 4.460363 -4.092342 -3.750795

H 1.472017 -0.943511 -3.329473

H 3.057985 -0.575370 -4.031717

H 1.940549 -1.636687 -4.897278

H 3.968267 -5.261510 -2.051674

H 5.532088 -4.460921 -1.777005

H 4.914615 -5.631687 -0.610345

H 5.866382 -3.042785 0.316565

H 4.495405 -2.547954 1.327823

H 5.030857 -4.231927 1.319628

H 2.881454 -5.159736 0.790891

H 2.078825 -3.583492 0.751056

H 1.938820 -4.686586 -0.628344

H 5.951442 -2.227405 -1.947708

H 5.164723 -0.869777 -2.779346

H 5.336683 2.520942 2.155590

H 4.618869 1.065559 2.878271

H 1.746367 3.355691 -1.094797

H 1.289418 4.457897 0.214198

H 2.361088 5.014513 -1.078404

H 4.651523 4.378673 -1.264254

H 5.482719 3.316982 -0.124056

H 4.348789 2.637140 -1.307188

H 4.084145 5.753035 0.615812

H 2.982459 5.259613 1.902673

H 4.659443 4.668485 1.883935

H 0.176603 3.380478 3.509514

H 1.059387 4.360501 2.333184

H 0.200695 2.884527 1.820784

H 0.897758 0.623575 2.851164

H 2.398582 0.475132 3.789345

H 1.024283 1.365222 4.457945

H 3.770633 2.577326 4.325380

H 3.307657 4.162794 3.675917

H 2.298707 3.367889 4.885906

H -8.066631 -2.140249 1.464488

H -10.055059 -1.892866 -0.014026

H -9.921745 -0.621140 -2.128930

H -7.791287 0.446092 -2.854123

H 10.039826 1.178170 1.422746

H 7.789005 1.831437 2.267145

H 8.282216 -1.249272 -1.688551

H 10.282083 -0.335509 -0.516837

H -3.616208 2.619352 2.882876

--------------------------------------------------------

**Cartesian coordinate of product complex for N_2_ elimination from A-PCP (A-PCP → B-PCP).** Units are presented in Å.

SCF energy = -4225.05697496 hartree

ZPE = 1.351437 hartree

SCF energy (in toluene) = -4226.00977212 hartree

-------------------------------------------------------

Atom Coordinates (Angstroms)

X Y Z

--------------------------------------------------------

C 7.949851 1.141398 1.033721

C 6.785801 0.560874 0.531553

C 6.835607 -0.547907 -0.342065

C 8.051057 -1.097371 -0.748301

C 9.218084 -0.511106 -0.246622

C 9.168371 0.586603 0.627988

N 5.430894 0.851778 0.709949

C 4.641668 -0.020254 0.006707

N 5.506996 -0.872838 -0.628567

Mo 2.500750 -0.040853 -0.083116

N 2.360841 -0.717064 1.830913

N 2.232036 -1.106943 2.887768

C 5.010938 -1.947126 -1.477867

P 3.256140 -2.402798 -0.907547

C 2.545562 -3.258211 -2.494833

C 3.570233 -4.117427 -3.266986

C 4.840468 1.921493 1.503381

P 3.130885 2.325261 0.779542

C 3.574414 3.689152 -0.525456

C 4.707478 3.147236 -1.430052

N 0.496709 0.063737 -0.066306

N -0.662980 0.187653 0.047680

Mo -2.637798 0.573763 0.357448

N -3.414337 -3.898260 -3.099352

N -2.603240 -4.652563 -3.096160

N -2.826022 2.095515 1.301240

N -2.656792 3.157728 1.932418

C -4.695638 0.039201 0.107561

N -5.512337 -0.495635 1.063545

C -6.799596 -0.689899 0.562948

C -6.762317 -0.271630 -0.786095

N -5.460139 0.173709 -1.017810

C -7.891630 -0.350488 -1.603867

C -9.061069 -0.848210 -1.026550

C -9.100521 -1.257148 0.319995

C -7.972251 -1.184102 1.138593

C -5.006779 -0.826459 2.391606

P -3.103499 -0.956622 2.305313

C -2.657903 -0.627587 4.155084

C -3.621058 -1.271568 5.176023

C -4.903596 0.676969 -2.268820

P -3.229941 1.534202 -1.911842

C -3.712656 3.396959 -1.978967

C -4.908532 3.595329 -1.019293

C -2.843487 -2.823362 1.876728

C -3.659549 -3.141923 0.603074

C -1.351309 -3.037769 1.544986

C -3.270248 -3.814587 2.975435

C -2.241611 1.000614 -3.476809

C -1.852302 -0.476238 -3.239571

C -3.001063 1.088595 -4.817640

C -0.952341 1.840949 -3.552256

N 2.507876 0.613117 -2.006726

N 2.487775 0.980176 -3.079822

C 3.618354 -3.773893 0.419182

C 4.649167 -3.206293 1.423906

C 2.309497 -4.101361 1.169993

C 4.209131 -5.085812 -0.136334

C 2.248257 3.165858 2.283237

C 1.695731 2.045219 3.184662

C 3.177798 4.046647 3.146935

C 1.045643 3.992700 1.783146

C -2.709798 0.903220 4.339559

C -1.216168 -1.096828 4.431562

C -4.118966 3.912095 -3.372749

C -2.516012 4.220744 -1.454781

C 2.054055 -2.149829 -3.445058

C 1.321912 -4.115525 -2.112010

C 4.078141 5.017085 0.076505

C 2.330968 3.983927 -1.393693

H -0.905140 -0.729858 5.420248

H -1.123727 -2.188637 4.450531

H -0.503627 -0.702746 3.696803

H -3.300556 -0.980853 6.186980

H -4.653389 -0.917681 5.060115

H -3.627343 -2.363985 5.140739

H -1.992082 1.422331 3.698799

H -3.704101 1.318679 4.129091

H -2.473723 1.141721 5.387042

H -2.639168 -3.747911 3.867341

H -4.316353 -3.685253 3.281306

H -3.173426 -4.839322 2.587638

H -4.742738 -3.070054 0.760624

H -3.392976 -2.486792 -0.236356

H -3.443100 -4.175048 0.295420

H -1.199731 -4.080295 1.228285

H -1.019675 -2.382681 0.730977

H -0.698348 -2.856771 2.404418

H -5.482593 -1.751452 2.738045

H -5.263676 -0.025046 3.095075

H -5.621578 1.357752 -2.740220

H -4.739017 -0.162221 -2.954104

H -2.230939 3.925335 -0.440063

H -1.637242 4.132263 -2.102463

H -2.799938 5.283360 -1.430711

H -5.106097 4.672504 -0.923587

H -5.830507 3.132229 -1.394044

H -4.704905 3.197525 -0.020532

H -4.500214 4.938699 -3.272290

H -3.272115 3.951577 -4.066749

H -4.917496 3.315731 -3.833749

H -0.279677 1.414925 -4.309666

H -1.153759 2.878913 -3.841652

H -0.412197 1.843347 -2.597732

H -1.244537 -0.596187 -2.335722

H -2.727977 -1.135503 -3.158920

H -1.260085 -0.832562 -4.094906

H -3.917218 0.483949 -4.828037

H -3.265878 2.111998 -5.094310

H -2.353210 0.694201 -5.613963

H 0.807459 -4.432836 -3.030334

H 1.593618 -5.023802 -1.564973

H 0.601646 -3.548705 -1.507427

H 3.074521 -4.540833 -4.152792

H 4.416094 -3.522619 -3.633828

H 3.965429 -4.952823 -2.685697

H 1.278722 -1.533239 -2.979692

H 2.861059 -1.489094 -3.779782

H 1.625837 -2.620505 -4.342467

H 3.502314 -5.642852 -0.759072

H 5.131563 -4.930258 -0.710405

H 4.469399 -5.735303 0.712147

H 5.639088 -3.073524 0.970544

H 4.341934 -2.254577 1.861641

H 4.765383 -3.926855 2.246077

H 2.540542 -4.756100 2.023033

H 1.821143 -3.206321 1.561512

H 1.590847 -4.632039 0.537463

H 5.699295 -2.798409 -1.427986

H 4.960753 -1.608944 -2.521226

H 5.510184 2.788953 1.500405

H 4.710735 1.593735 2.542975

H 1.930155 3.083840 -1.865019

H 1.527624 4.457059 -0.820578

H 2.615786 4.679777 -2.196425

H 4.871559 3.865145 -2.246310

H 5.657652 3.052155 -0.889917

H 4.473394 2.181695 -1.883577

H 4.403993 5.670249 -0.746075

H 3.300387 5.556515 0.625340

H 4.943288 4.883656 0.738516

H 0.434926 4.291601 2.646060

H 1.348301 4.909045 1.265966

H 0.391549 3.412074 1.121227

H 0.966552 1.423961 2.655456

H 2.482527 1.394963 3.583345

H 1.186328 2.507068 4.042864

H 3.988946 3.466662 3.605462

H 3.619598 4.883912 2.602323

H 2.585763 4.469736 3.971361

H -8.009516 -1.495461 2.179637

H -10.031864 -1.637711 0.732943

H -9.962044 -0.921603 -1.631133

H -7.865170 -0.039065 -2.645215

H 10.096149 1.015218 0.999961

H 7.917021 1.987533 1.716015

H 8.095588 -1.943356 -1.430162

H 10.183697 -0.914676 -0.542550

H -3.559125 3.619418 2.140981

--------------------------------------------------------

**Cartesian coordinate of B-PCP.** Units are presented in Å.

SCF energy = -4115.59636993 hartree

ZPE = 1.346011 hartree

SCF energy (in toluene) = -4116.51111490 hartree

-------------------------------------------------------

Atom Coordinates (Angstroms)

X Y Z

--------------------------------------------------------

C -8.031432 -1.696850 0.483951

C -6.864960 -1.029417 0.103649

C -6.833783 -0.194896 -1.035609

C -7.963024 -0.014250 -1.837162

C -9.126402 -0.684924 -1.456162

C -9.159878 -1.510695 -0.316335

N -5.537281 0.316273 -1.105877

C -4.770497 -0.165286 -0.081469

N -5.579604 -0.992927 0.644371

Mo -2.724627 0.306041 0.351332

P -3.166159 -1.762306 1.720310

C -2.826596 -3.388988 0.729390

C -3.257944 -4.687977 1.435217

C -5.074751 -1.727270 1.799193

C -4.988669 1.198458 -2.129640

P -3.316834 1.903265 -1.522645

C -2.318455 1.855814 -3.170190

C -1.022507 2.665779 -2.974321

N -2.964553 1.462310 1.710381

N -2.833164 2.281344 2.641564

N -0.734684 0.070262 -0.018392

N 0.428712 -0.001486 -0.138055

Mo 2.436491 -0.047652 -0.138700

N 2.441949 1.052977 -1.847460

N 2.419195 1.665375 -2.801841

C -3.793774 3.704246 -1.030456

C -2.600154 4.323178 -0.270196

C -5.000374 3.611526 -0.067988

C -4.180979 4.618678 -2.207165

N 2.305977 -1.179304 1.545834

N 2.188169 -1.823279 2.471546

C 4.571623 0.031140 -0.000321

N 5.315551 0.733897 0.911491

C 6.683331 0.540196 0.702309

C 6.789998 -0.321249 -0.411956

N 5.479834 -0.610606 -0.802428

C 8.032012 -0.717814 -0.906262

C 9.167413 -0.234198 -0.247157

C 9.061342 0.617064 0.864751

C 7.815971 1.018484 1.359995

C 4.671218 1.552329 1.930253

P 2.953462 2.050348 1.290602

C 3.353633 3.712054 0.371709

C 3.798747 4.865976 1.293012

C 5.041231 -1.473432 -1.890603

P 3.300352 -2.119296 -1.480800

C 2.639194 -2.597751 -3.238713

C 1.454765 -3.575487 -3.094159

C 2.019113 2.457226 2.936064

C 0.786815 3.333294 2.628232

C 2.896501 3.136502 4.010811

C 1.504990 1.129081 3.523286

C 3.722101 -3.752056 -0.517106

C 2.429626 -4.320321 0.108009

C 4.717678 -3.402561 0.614036

C 4.381112 -4.855355 -1.370260

C 4.514840 3.450730 -0.617373

C 2.108740 4.166163 -0.421377

C 2.100301 -1.314977 -3.899643

C 3.704390 -3.197926 -4.182937

C -2.755946 -2.014140 3.586416

C -1.297610 -2.491294 3.729164

C -3.697959 -2.983244 4.331892

C -2.870055 -0.620962 4.239649

C -3.592428 -3.294496 -0.611219

C -1.319212 -3.462854 0.404988

C -1.942975 0.371751 -3.386905

C -3.059273 2.354009 -4.428728

H -1.005503 -2.437795 4.787644

H -1.161740 -3.530854 3.409518

H -0.601359 -1.860005 3.163719

H -3.389779 -3.025971 5.386630

H -4.741753 -2.644989 4.322532

H -3.665324 -4.004127 3.942858

H -2.174621 0.098695 3.799266

H -3.880530 -0.198063 4.160115

H -2.641571 -0.713213 5.311463

H -2.652674 -4.900660 2.322334

H -4.315059 -4.683259 1.730858

H -3.122330 -5.532019 0.743098

H -4.681456 -3.281181 -0.480403

H -3.307174 -2.407069 -1.192017

H -3.347968 -4.176442 -1.220790

H -1.137172 -4.330881 -0.245991

H -0.969499 -2.565655 -0.118531

H -0.701772 -3.588851 1.299591

H -5.519407 -2.729571 1.810598

H -5.370039 -1.212328 2.721362

H -5.711764 1.990633 -2.354198

H -4.825349 0.624566 -3.048967

H -2.331574 3.737642 0.614950

H -1.712398 4.422479 -0.904318

H -2.877906 5.333640 0.064735

H -5.189407 4.608836 0.354043

H -5.921458 3.300235 -0.577501

H -4.815572 2.922312 0.761561

H -4.560109 5.569386 -1.805150

H -3.324477 4.859151 -2.846234

H -4.974826 4.194805 -2.836021

H -0.337546 2.474460 -3.811988

H -1.212991 3.745101 -2.947043

H -0.502282 2.380545 -2.052161

H -1.349653 -0.022815 -2.554420

H -2.824761 -0.271810 -3.520481

H -1.340295 0.283718 -4.302399

H -3.973957 1.784204 -4.637903

H -3.322500 3.413152 -4.379392

H -2.399901 2.222581 -5.298976

H 0.152860 3.385980 3.523837

H 1.053700 4.360065 2.356242

H 0.167741 2.905184 1.830482

H 2.277010 3.311950 4.902291

H 3.732955 2.500899 4.328785

H 3.299080 4.103401 3.701311

H 0.818852 0.622693 2.837776

H 2.314317 0.436075 3.779442

H 0.956166 1.348065 4.450388

H 2.994579 5.224506 1.942385

H 4.660536 4.602406 1.919311

H 4.108295 5.713554 0.664454

H 5.462209 3.253807 -0.100852

H 4.318651 2.623380 -1.302333

H 4.661792 4.355141 -1.224933

H 2.378638 5.033479 -1.041634

H 1.735834 3.385988 -1.088631

H 1.289360 4.473914 0.235128

H 5.302021 2.420803 2.152466

H 4.547905 0.974585 2.855733

H 5.762746 -2.287812 -2.021901

H 4.994621 -0.904788 -2.828700

H 1.914259 -3.588003 0.733017

H 1.725946 -4.678092 -0.649802

H 2.689494 -5.178011 0.745729

H 4.858174 -4.296147 1.238645

H 5.704493 -3.123757 0.225201

H 4.365444 -2.599187 1.263962

H 4.683837 -5.675942 -0.703557

H 3.701748 -5.283172 -2.114429

H 5.288210 -4.512486 -1.884055

H 0.963592 -3.686220 -4.071811

H 1.765616 -4.575175 -2.774249

H 0.703330 -3.201976 -2.385836

H 1.295238 -0.863982 -3.310839

H 2.876955 -0.557955 -4.052404

H 1.697606 -1.572775 -4.890205

H 4.528179 -2.498857 -4.374095

H 4.128987 -4.138712 -3.825843

H 3.232607 -3.402217 -5.155251

H -8.064211 -2.330939 1.366472

H -10.086781 -2.014361 -0.051994

H -10.027450 -0.566177 -2.053394

H -7.940707 0.620798 -2.719355

H 10.152439 -0.523974 -0.605768

H 8.120039 -1.371785 -1.770661

H 7.739872 1.671976 2.225814

H 9.965728 0.971734 1.353758

H -3.750691 2.629105 2.970009

--------------------------------------------------------

**Cartesian coordinate of C-PCP.** Units are presented in Å.

SCF energy = -5076.85145942 hartree

ZPE = 1.375164 hartree

SCF energy (in toluene) = -5077.97230008 hartree

-------------------------------------------------------

Atom Coordinates (Angstroms)

X Y Z

--------------------------------------------------------

C -8.307005 1.469774 -0.703656

C -7.144628 0.776230 -0.370374

C -7.200969 -0.517798 0.200899

C -8.421093 -1.138885 0.463133

C -9.587645 -0.437769 0.130512

C -9.531683 0.841417 -0.442178

N -5.792394 1.092245 -0.479347

C -4.996404 0.062904 -0.019582

N -5.879153 -0.914852 0.390092

Mo -2.891823 -0.004681 0.027956

N -2.767696 -0.158723 -1.995041

N -2.654947 -0.261948 -3.120913

C -5.385569 -2.175383 0.923288

P -3.625215 -2.459210 0.245109

C -4.017329 -3.457386 -1.378206

C -4.619720 -4.860504 -1.160404

C -5.195323 2.323344 -0.973701

P -3.456663 2.486987 -0.201272

C -2.605844 3.693857 -1.459037

C -1.393332 4.359885 -0.779238

N -0.825180 -0.150055 0.061812

N 0.316195 -0.299235 0.080760

Mo 2.357050 -0.745790 0.156303

N 2.108681 -2.492794 0.495168

N 1.721484 -3.658399 0.744281

C 4.499758 -0.535962 0.105354

N 5.286829 -0.558669 -1.010928

C 6.592038 -0.178425 -0.695228

C 6.600400 0.087866 0.689631

N 5.300864 -0.145277 1.139405

C 7.744193 -0.036224 -1.469784

C 8.906490 0.373365 -0.814206

C 8.914982 0.638614 0.567955

C 7.761817 0.503541 1.342597

C 4.727331 -0.617272 -2.365126

P 2.936084 -1.253695 -2.309029

C 2.150446 -0.370873 -3.845265

C 3.094252 -0.198181 -5.054231

C 4.747685 0.313544 2.416342

P 2.956064 -0.298272 2.603357

C 2.168133 1.116861 3.668981

C 3.107939 1.749744 4.718025

C 3.238373 -1.845726 3.731232

C 1.919955 -2.637053 3.883197

C 3.781856 -1.521177 5.138191

C 4.280357 -2.749368 3.031053

C 3.223241 -3.120337 -2.745220

C 1.904962 -3.905541 -2.565890

C 4.278921 -3.673020 -1.758128

C 3.756520 -3.377713 -4.169773

N -2.820946 0.147747 2.052680

N -2.733485 0.247265 3.181149

C -2.938412 -3.717109 1.559117

C -1.756373 -4.508862 0.965782

C -3.993123 -4.695239 2.122298

C -2.389136 -2.891641 2.737550

C -3.871168 3.501324 1.408070

C -2.614325 3.568286 2.303576

C -4.990367 2.753500 2.170687

C -4.378582 4.937186 1.165038

C -5.051631 -2.654673 -2.202128

C -2.715072 -3.595722 -2.197380

C -2.068859 2.841244 -2.624901

C -3.532367 4.772076 -2.062488

C 1.740878 2.230861 2.691735

C 0.897889 0.581812 4.360288

C 0.883773 -1.139979 -4.270942

C 1.715399 1.034267 -3.382908

O 4.383983 2.480349 -1.812262

S 3.609964 2.606549 -0.558505

O 4.382102 2.915850 0.664083

O 2.558681 1.534981 -0.377448

C 2.532943 4.107084 -0.825412

F 1.755567 3.947530 -1.910741

F 1.736687 4.317105 0.238269

F 3.299309 5.189916 -1.004024

H 2.120461 -3.539803 4.481673

H 1.149821 -2.065295 4.409418

H 1.520109 -2.960637 2.918452

H 4.028416 -2.466912 5.644032

H 4.699617 -0.919026 5.117239

H 3.045740 -1.005190 5.762869

H 3.991023 -2.997018 2.007212

H 5.278381 -2.293505 3.004618

H 4.367770 -3.688084 3.598192

H 3.449708 1.045787 5.481582

H 3.987957 2.213728 4.255835

H 2.562839 2.553227 5.236025

H 2.596463 2.688627 2.188436

H 1.051636 1.864114 1.924127

H 1.227225 3.017403 3.265631

H 0.336111 1.430069 4.777534

H 0.232119 0.067883 3.655597

H 1.119385 -0.099049 5.189601

H 4.739991 1.408473 2.384417

H 5.390699 -0.021579 3.237634

H 5.368719 -1.240330 -2.998860

H 4.722597 0.407531 -2.752767

H 1.482163 -3.785188 -1.564973

H 1.146570 -3.612248 -3.297897

H 2.107243 -4.977296 -2.719458

H 4.347383 -4.763472 -1.887063

H 5.278837 -3.262779 -1.948845

H 4.027702 -3.462680 -0.715548

H 4.008277 -4.445131 -4.261535

H 3.014793 -3.155661 -4.943597

H 4.669787 -2.811953 -4.395414

H 2.551574 0.338832 -5.846764

H 3.973445 0.409626 -4.806491

H 3.437899 -1.144750 -5.479936

H 1.108670 -2.094318 -4.759803

H 0.215754 -1.330480 -3.421311

H 0.322543 -0.528699 -4.992108

H 1.051506 0.991755 -2.513698

H 2.569508 1.666019 -3.125893

H 1.172182 1.518635 -4.208482

H -1.285858 -5.093468 1.769944

H -2.066752 -5.218203 0.191246

H -0.974450 -3.857835 0.557261

H -3.505601 -5.339448 2.869305

H -4.810276 -4.175349 2.638543

H -4.429669 -5.350419 1.364514

H -1.599400 -2.206457 2.414363

H -3.165342 -2.306932 3.244029

H -1.958072 -3.579476 3.480343

H -3.917506 -5.559271 -0.695738

H -5.536232 -4.836891 -0.556381

H -4.892321 -5.280473 -2.140458

H -6.035964 -2.632145 -1.717988

H -4.741820 -1.625988 -2.393509

H -5.181192 -3.149728 -3.176091

H -2.951713 -4.029924 -3.180659

H -2.232242 -2.630305 -2.366769

H -1.988710 -4.254814 -1.711211

H -6.077916 -2.982798 0.655956

H -5.328243 -2.124156 2.018502

H -5.848069 3.170344 -0.730681

H -5.087135 2.279241 -2.065670

H -2.195217 2.579655 2.503485

H -1.826080 4.189081 1.866936

H -2.888290 4.016017 3.271026

H -5.146860 3.253717 3.137973

H -5.946912 2.782258 1.633974

H -4.743409 1.709647 2.374233

H -4.684684 5.368899 2.129942

H -3.609006 5.595019 0.749219

H -5.256246 4.971139 0.506564

H -0.769676 4.843206 -1.543709

H -1.684215 5.131839 -0.058277

H -0.760635 3.626846 -0.265765

H -1.355996 2.085399 -2.281447

H -2.865284 2.332622 -3.179822

H -1.546455 3.502479 -3.332262

H -4.360140 4.332635 -2.633900

H -3.954097 5.454651 -1.321014

H -2.947672 5.379309 -2.769856

H 7.767285 0.723370 2.407267

H 9.838771 0.963240 1.042247

H 9.823704 0.497375 -1.386048

H 7.735792 -0.224998 -2.540351

H -10.456506 1.357976 -0.690722

H -8.267764 2.459247 -1.153521

H -8.468900 -2.128210 0.912441

H -10.555361 -0.896681 0.322355

H 2.481278 -4.350152 0.619995

--------------------------------------------------------

**Cartesian coordinate of reactant complex for proton transfer from LutH^+^ to 2 (2 → A-PNP).** Units are presented in Å.

SCF energy = -4288.84326889 hartree

ZPE = 1.442248 hartree

SCF energy (in toluene) = -4289.81316214 hartree

-------------------------------------------------------

Atom Coordinates (Angstroms)

X Y Z

--------------------------------------------------------

Mo 3.023143 0.324860 -0.114354

Mo -1.982360 -1.047237 0.575512

P -2.454856 -2.387444 -1.537800

P 2.936292 2.860432 0.010507

N 5.180745 0.897697 -0.397384

N -4.069569 -1.764061 0.941828

P -2.462602 -0.094942 2.885671

P 4.172276 -1.947833 -0.359632

N 1.077819 -0.175229 0.133870

N -0.035535 -0.459186 0.272783

N 2.540165 0.493095 -3.233146

N 2.715942 0.439348 -2.110360

N 3.295863 0.197263 1.888287

N 3.445606 0.139459 3.013881

N -3.201070 1.452062 -0.839999

N -2.716163 0.539151 -0.326652

N -1.271218 -2.734190 1.517730

N -0.897721 -3.676498 2.021630

H -2.630823 -1.628386 -5.357380

H 1.230054 5.725584 -2.009339

H 2.800529 4.986495 -2.327431

H -3.954074 -1.557818 -4.188687

C -2.994568 -2.045449 -4.406430

C 2.075880 5.205692 -1.533849

H 1.621688 2.793650 -2.758056

H -2.672189 0.290700 -3.012608

H 2.536988 5.907582 -0.834396

H -3.185894 -3.108561 -4.570547

H 0.087395 3.599136 -2.416775

H -1.370398 0.114396 -4.202121

C -1.740539 -0.243603 -3.230142

C 0.918097 3.041102 -1.957242

H -0.222112 -1.894829 -4.614096

H -0.403196 4.710086 -0.403005

C -1.945552 -1.768862 -3.308831

C 1.554383 3.917874 -0.861209

H 4.004146 2.926754 -2.148955

H -4.387292 -1.004505 -1.876458

H -1.008953 0.030262 -2.463566

H 0.520175 2.104560 -1.552260

H -4.895785 -2.667399 -2.183366

H 4.793973 4.142861 -1.144489

C -0.591042 -2.390976 -3.704902

C 0.441930 4.267473 0.146633

H -2.276426 -4.820964 -3.688849

H 2.644872 5.824611 1.306711

H 0.166746 -2.246094 -2.924627

H 0.067960 3.376331 0.667038

C -4.310248 -2.035501 -1.503462

C 4.404840 3.117193 -1.142990

H -6.799405 -2.820757 -0.764843

H 7.073573 3.514702 -1.418904

H -0.663293 -3.460599 -3.925469

H 0.757023 5.001031 0.894994

H -3.935611 -4.693988 -3.058976

H 4.285029 5.607519 0.654470

H 4.962419 -0.542139 -2.864920

H -4.669044 1.006886 1.235404

C -2.910450 -5.001106 -2.814989

C 3.605738 5.325673 1.469293

H 3.721921 -3.185725 -3.960855

H -3.387131 3.591032 2.449803

C -6.191074 -2.550300 0.095289

C 6.851012 2.517584 -1.046281

H -2.932116 -6.089151 -2.654159

H 4.028435 5.738416 2.397316

C -4.880187 -2.113360 -0.106133

C 5.519987 2.141280 -0.855689

H 5.667996 -1.814262 -3.869990

H -5.380889 2.280860 2.244382

H 2.769003 -2.032045 -3.015861

H -2.463474 2.487722 1.403018

C 5.491813 -1.497212 -2.831718

C -4.840938 1.321809 2.267130

C 3.422884 -2.905317 -2.939705

C -2.748695 2.692977 2.439033

H 8.918233 1.900665 -0.885253

H -7.714717 -3.004287 1.563469

C -2.379363 -4.326607 -1.533487

C 3.474853 3.796380 1.624575

H 6.473264 -1.332616 -2.372519

H -5.505323 0.591693 2.743053

C 7.875903 1.620881 -0.749532

C -6.699122 -2.655687 1.389668

H 2.840974 -3.736549 -2.530384

H -1.840420 2.937072 2.998097

H -0.259213 -4.506088 -2.124891

H 1.473758 3.899523 2.556057

H -4.319549 -4.699657 -0.556501

H 5.655042 3.589960 1.366819

C 4.691393 -2.605942 -2.110449

C -3.529793 1.515120 3.061588

C -0.921666 -4.778776 -1.297909

C 2.467552 3.486392 2.753944

C -3.249756 -4.841148 -0.363812

C 4.864099 3.269929 2.054666

H -3.079483 -5.921398 -0.249088

H 5.103024 3.688781 3.042966

H 5.918754 -4.080197 -3.107964

H -4.608937 2.746714 4.474993

C -5.871483 -2.307457 2.455691

C 7.531523 0.357514 -0.273384

H -0.504497 -4.362220 -0.377877

H 2.363316 2.412082 2.926924

C -4.570647 -1.860618 2.212823

C 6.186785 0.015242 -0.113767

C 5.587051 -3.861045 -2.082207

C -3.931545 1.879920 4.504956

H 6.490212 -3.717460 -1.475384

H -4.473305 1.067196 5.005267

H -0.897723 -5.874781 -1.205786

H 2.829124 3.938951 3.689402

H 8.298247 -0.373591 -0.028218

H -6.226547 -2.376387 3.481273

H -3.009213 -4.366413 0.589946

H 4.903019 2.181515 2.140530

H 3.188344 -4.940884 -0.797153

H -1.018636 2.028221 4.761326

C 5.805589 -1.359832 0.375396

C -3.676432 -1.462305 3.361915

H 5.064162 -4.750381 -1.717268

H -3.077585 2.160124 5.128697

H 6.635067 -2.059441 0.214552

H -4.282466 -1.227779 4.245857

H 1.876176 -3.836023 -0.327579

H 0.024662 1.436816 3.445096

C 2.744039 -4.395806 0.041979

C -0.412919 1.182709 4.419032

H 5.624530 -1.323866 1.459169

H -3.036461 -2.313450 3.636868

C 3.750880 -3.478129 0.763206

C -1.200420 -0.143179 4.361519

H 2.178250 -2.345939 1.800753

H 0.315959 -1.122650 3.106448

H 2.371235 -5.145028 0.755316

H 0.417859 1.070314 5.129902

C 3.061111 -2.949425 2.035672

C -0.178334 -1.260733 4.073118

H 5.533036 -4.740093 0.369458

H -2.557523 0.317165 6.058596

H 5.683353 -3.697772 1.797417

H -2.340435 -1.416535 5.751946

C 4.982814 -4.297180 1.203524

C -1.839496 -0.440493 5.735199

H 2.736883 -3.806895 2.643345

H 0.595711 -1.237128 4.853365

H 3.731077 -2.346130 2.656637

H -0.628972 -2.258337 4.095319

H 4.644358 -5.122280 1.847521

H -1.042332 -0.478096 6.492100

H -4.325689 2.675324 -1.693765

C -6.712805 3.880112 -3.622758

C -6.544444 5.241763 -3.336359

C -5.545904 5.643511 -2.439380

C -4.729500 4.696435 -1.838037

N -4.928517 3.379215 -2.154331

C -5.890657 2.938233 -3.022971

H -7.480975 3.545419 -4.314105

H -7.186345 5.982086 -3.806943

H -5.397082 6.693141 -2.202451

C -3.641548 5.006569 -0.857354

H -2.684384 4.572587 -1.177935

H -3.519985 6.088145 -0.747851

H -3.867076 4.579730 0.129878

C -5.974581 1.461115 -3.259360

H -5.060948 1.085961 -3.741981

H -6.090629 0.917100 -2.311182

H -6.825630 1.223291 -3.904255

--------------------------------------------------------

**Cartesian coordinate of transition state for proton transfer from LutH^+^ to 2 (2 → A-PNP).**  Units are presented in Å.

SCF energy = -4288.82852770 hartree

ZPE = 1.437114 hartree

SCF energy (in toluene) = -4289.79502953 hartree

Imaginary frequency: 1087i cm^-1^

-------------------------------------------------------

Atom Coordinates (Angstroms)

X Y Z

--------------------------------------------------------

Mo 3.067889 0.342202 -0.079213

Mo -2.005556 -0.905026 0.416849

P -2.336603 -2.413179 -1.617619

P 2.961723 2.862964 -0.390804

N 5.248496 0.897163 -0.318716

N -4.042086 -1.748015 0.799888

P -2.585187 0.155495 2.657907

P 4.243780 -1.926850 0.117392

N 1.125141 -0.107263 0.106372

N -0.005573 -0.340090 0.190410

N 2.777626 -0.022904 -3.206656

N 2.888352 0.116314 -2.083592

N 3.209006 0.545243 1.931599

N 3.280776 0.665628 3.060105

N -2.945098 1.559613 -1.171464

N -2.656632 0.583106 -0.559742

N -1.182378 -2.532351 1.492825

N -0.730907 -3.397803 2.052913

H -2.498836 -1.884090 -5.473747

H 1.401124 5.345628 -2.955160

H 3.012018 4.630964 -3.012628

H -3.842372 -1.848707 -4.330328

C -2.848633 -2.279499 -4.508729

C 2.208352 4.934118 -2.330535

H 1.909463 2.357489 -3.230390

H -2.697193 0.151064 -3.248679

H 2.581588 5.748563 -1.704807

H -2.961216 -3.360079 -4.623396

H 0.343615 3.170053 -3.149230

H -1.381318 -0.016381 -4.419339

C -1.732647 -0.335811 -3.427286

C 1.131496 2.700798 -2.541044

H -0.084638 -1.962448 -4.683124

H -0.354803 4.523950 -1.360594

C -1.836824 -1.872730 -3.415849

C 1.651835 3.738800 -1.527180

H 4.197194 2.617435 -2.445550

H -4.352323 -1.192792 -2.057549

H -1.025489 0.035099 -2.679140

H 0.701840 1.824591 -2.045381

H -4.743104 -2.900537 -2.275785

H 4.899427 3.983092 -1.576383

C -0.439270 -2.430508 -3.753939

C 0.444956 4.193677 -0.681571

H -1.988157 -4.940930 -3.614002

H 2.528486 5.980337 0.401538

H 0.294352 -2.195069 -2.972596

H 0.042032 3.373045 -0.073981

C -4.211666 -2.193700 -1.626011

C 4.516311 2.962929 -1.451162

H -6.649224 -3.115353 -0.875304

H 7.191098 3.353191 -1.609089

H -0.442268 -3.512738 -3.917103

H 0.677663 5.036635 -0.022836

H -3.659228 -4.861457 -3.010907

H 4.225325 5.718014 -0.059126

H 5.154740 -0.912691 -2.511369

H -4.711944 0.999802 0.834099

C -2.624580 -5.107863 -2.739864

C 3.477253 5.540025 0.724089

H 3.967231 -3.732915 -3.247937

H -3.692152 3.741201 1.936440

C -6.079886 -2.744297 -0.026317

C 6.950170 2.420863 -1.103894

H -2.596523 -6.185688 -2.522161

H 3.805293 6.097504 1.614018

C -4.798944 -2.228932 -0.236308

C 5.610938 2.060146 -0.939441

H 5.882001 -2.317061 -3.303581

H -5.567635 2.305702 1.682509

H 2.964912 -2.455745 -2.544244

H -2.622705 2.642590 1.035224

C 5.676098 -1.854489 -2.327370

C -4.983606 1.383923 1.820776

C 3.621782 -3.294708 -2.299782

C -2.995361 2.893869 2.032784

H 9.005656 1.858616 -0.733427

H -7.599080 -3.204493 1.443713

C -2.141786 -4.340362 -1.492076

C 3.354018 4.048441 1.095226

H 6.644053 -1.627406 -1.865632

H -5.641198 0.652733 2.305287

C 7.957813 1.590404 -0.617973

C -6.607113 -2.795932 1.263681

H 3.028292 -4.056897 -1.785698

H -2.149891 3.238139 2.635088

H -0.006080 -4.418336 -2.056717

H 1.286690 4.262899 1.842266

H -4.063474 -4.787135 -0.507704

H 5.548806 3.848148 1.039249

C 4.850756 -2.847602 -1.476926

C -3.741815 1.706210 2.681113

C -0.662382 -4.696492 -1.227128

C 2.258998 3.885157 2.172700

C -2.988732 -4.845288 -0.301251

C 4.709022 3.624190 1.707005

H -2.749235 -5.903805 -0.124720

H 4.868726 4.196810 2.632359

H 6.125401 -4.443105 -2.183732

H -4.996679 2.926228 3.951024

C -5.831848 -2.316284 2.317288

C 7.589217 0.408077 0.019967

H -0.279478 -4.220264 -0.321119

H 2.135217 2.842091 2.474841

C -4.561014 -1.792504 2.065887

C 6.237601 0.080399 0.152325

C 5.752892 -4.070938 -1.217741

C -4.250164 2.127228 4.073874

H 6.632635 -3.822940 -0.610379

H -4.743838 1.306161 4.609033

H -0.576418 -5.784510 -1.090129

H 2.547272 4.458876 3.066078

H 8.341599 -0.269751 0.416089

H -6.205224 -2.339334 3.338292

H -2.790851 -4.304782 0.627360

H 4.748221 2.563789 1.965953

H 3.298974 -4.963870 0.134336

H -1.303687 2.477948 4.406314

C 5.832694 -1.202757 0.831625

C -3.727107 -1.251269 3.201518

H 5.223278 -4.899841 -0.737974

H -3.456879 2.528269 4.712116

H 6.672546 -1.908324 0.833813

H -4.372580 -0.993370 4.050200

H 1.962380 -3.819018 0.385355

H -0.209284 1.852722 3.147227

C 2.824444 -4.300020 0.864087

C -0.647069 1.645463 4.132048

H 5.590335 -0.992088 1.883970

H -3.042236 -2.034730 3.558601

C 3.801123 -3.262955 1.455673

C -1.368482 0.282592 4.164396

H 2.185495 -2.007341 2.255807

H 0.223482 -0.728329 3.031648

H 2.439227 -4.932415 1.676848

H 0.177116 1.629551 4.858996

C 3.070703 -2.546087 2.607807

C -0.295879 -0.810785 3.991598

H 5.602782 -4.558939 1.335594

H -2.790865 0.819628 5.781889

H 5.699659 -3.292950 2.575417

H -2.487589 -0.922686 5.633266

C 5.022036 -3.987565 2.063989

C -2.031736 0.071138 5.542250

H 2.745629 -3.298148 3.341636

H 0.453326 -0.699673 4.788195

H 3.715126 -1.834990 3.134918

H -0.711725 -1.820566 4.080636

H 4.663983 -4.698339 2.823659

H -1.254333 0.135564 6.317614

H -4.029836 2.085841 -1.424080

C -7.402823 3.210098 -2.299107

C -7.186492 4.585398 -2.379503

C -5.905008 5.087534 -2.162060

C -4.863304 4.206096 -1.858587

N -5.098332 2.874733 -1.779352

C -6.331990 2.365386 -1.999582

H -8.388624 2.786429 -2.472130

H -8.008657 5.258346 -2.614454

H -5.702145 6.153299 -2.226627

C -3.459518 4.687385 -1.609585

H -2.737949 4.130083 -2.218292

H -3.371743 5.754166 -1.838660

H -3.173849 4.533563 -0.560914

C -6.492734 0.871084 -1.934940

H -5.927103 0.388314 -2.743475

H -6.124103 0.467777 -0.984004

H -7.544527 0.587398 -2.043020

--------------------------------------------------------

**Cartesian coordinate of product complex for proton transfer from LutH^+^ to 2 (2 → A-PNP).** Units are presented in Å.

SCF energy = -4288.83502974 hartree

ZPE = 1.440569 hartree

SCF energy (in toluene) = -4289.80139428 hartree

-------------------------------------------------------

Atom Coordinates (Angstroms)

X Y Z

--------------------------------------------------------

Mo 3.086109 0.397505 -0.080724

Mo -1.923941 -0.975100 0.377947

P -2.283782 -2.261034 -1.811983

P 2.790716 2.922281 -0.374384

N 5.218383 1.117396 -0.345413

N -3.906310 -1.966204 0.749396

P -2.473169 -0.151291 2.727439

P 4.439636 -1.784077 0.080044

N 1.192063 -0.163300 0.119551

N 0.066637 -0.436176 0.206713

N 2.789880 0.029531 -3.206366

N 2.902803 0.168529 -2.084366

N 3.236842 0.601556 1.931431

N 3.313669 0.717159 3.059148

N -2.880995 1.647896 -0.874717

N -2.606453 0.563995 -0.389208

N -1.021660 -2.734825 1.296383

N -0.504726 -3.613609 1.762944

H -2.710480 -1.390155 -5.575862

H 1.003520 5.280798 -2.897359

H 2.660726 4.684084 -2.992622

H -3.986724 -1.556968 -4.370165

C -2.976885 -1.894415 -4.635559

C 1.852456 4.929366 -2.292525

H 1.736004 2.339243 -3.184547

H -2.952443 0.405335 -3.128604

H 2.179167 5.768122 -1.672858

H -3.017765 -2.965909 -4.843226

H 0.109219 3.021628 -3.074250

H -1.720926 0.472883 -4.394109

C -1.966926 0.026764 -3.419392

C 0.944506 2.620271 -2.482287

H -0.270213 -1.306055 -4.926209

H -0.662181 4.319010 -1.270589

C -1.937048 -1.506305 -3.562348

C 1.398540 3.697565 -1.479524

H 4.007723 2.753770 -2.447592

H -4.398916 -1.135070 -2.007695

H -1.239477 0.386406 -2.686621

H 0.595328 1.715889 -1.975039

H -4.683913 -2.832860 -2.399373

H 4.620526 4.170935 -1.593988

C -0.518664 -1.893632 -4.031179

C 0.177827 4.061847 -0.609902

H -1.819590 -4.531590 -4.073238

H 2.168364 5.997190 0.437465

H 0.239813 -1.661923 -3.273000

H -0.148947 3.217789 0.009735

C -4.164424 -2.165062 -1.701017

C 4.316002 3.125959 -1.459277

H -6.509057 -3.299555 -0.953436

H 6.952316 3.716314 -1.650771

H -0.429272 -2.949502 -4.306480

H 0.360689 4.925786 0.037487

H -3.460536 -4.691963 -3.407277

H 3.865465 5.840615 -0.069523

H 5.226731 -0.691306 -2.584282

H -4.708803 0.783361 1.045309

C -2.394697 -4.851716 -3.199601

C 3.151512 5.618561 0.733997

H 4.234174 -3.543952 -3.311499

H -3.768329 3.438179 2.441881

C -5.921265 -2.984859 -0.094756

C 6.788881 2.767104 -1.146210

H -2.246597 -5.936688 -3.096009

H 3.468251 6.197322 1.614179

C -4.677511 -2.382667 -0.300478

C 5.482611 2.306658 -0.964631

H 6.073255 -2.034489 -3.359997

H -5.587008 1.933999 2.063913

H 3.142463 -2.384517 -2.539852

H -2.716792 2.516507 1.350801

C 5.824954 -1.583797 -2.388177

C -4.953886 1.035463 2.080366

C 3.878032 -3.168378 -2.340841

C -3.034369 2.619364 2.392515

H 8.885389 2.358683 -0.806663

H -7.352110 -3.659709 1.378250

C -1.939459 -4.167604 -1.893929

C 3.132221 4.122939 1.109514

H 6.768792 -1.271839 -1.926246

H -5.555769 0.221896 2.503322

C 7.862316 2.012179 -0.678423

C -6.389641 -3.183952 1.202490

H 3.368418 -3.996348 -1.838472

H -2.168655 2.926118 2.986351

H 0.160002 -4.042187 -2.557105

H 1.065066 4.184871 1.885870

H -3.788004 -4.863373 -0.917314

H 5.334990 4.077248 1.022704

C 5.085671 -2.640356 -1.534066

C -3.698300 1.331126 2.931216

C -0.424097 -4.404822 -1.706393

C 2.068988 3.891051 2.206289

C -2.705298 -4.865575 -0.746142

C 4.521807 3.787117 1.697821

H -2.386984 -5.916985 -0.702392

H 4.653786 4.358107 2.628324

H 6.453829 -4.138068 -2.277188

H -4.958432 2.347452 4.361173

C -5.601607 -2.759326 2.269292

C 7.592806 0.802032 -0.043441

H -0.034842 -3.919342 -0.807000

H 2.034016 2.846880 2.528095

C -4.370420 -2.145083 2.022585

C 6.271557 0.373032 0.104406

C 6.079197 -3.796106 -1.301229

C -4.172294 1.578413 4.377256

H 6.952489 -3.485206 -0.714031

H -4.606944 0.683693 4.840734

H -0.235998 -5.484450 -1.613468

H 2.324910 4.500968 3.085307

H 8.399455 0.180136 0.337183

H -5.935127 -2.891184 3.295567

H -2.517188 -4.426890 0.236123

H 4.638112 2.728679 1.941684

H 3.726275 -4.883261 0.076837

H -1.224322 2.046490 4.647164

C 5.975534 -0.943857 0.776806

C -3.529654 -1.654450 3.175805

H 5.623374 -4.661938 -0.811891

H -3.372999 1.950689 5.025311

H 6.867251 -1.582205 0.756903

H -4.165405 -1.493654 4.055389

H 2.307871 -3.852245 0.368565

H -0.145018 1.590035 3.306668

C 3.217108 -4.265987 0.823975

C -0.543866 1.272017 4.278283

H 5.738451 -0.762822 1.835492

H -2.804617 -2.431781 3.458315

C 4.120336 -3.160079 1.410131

C -1.209908 -0.117314 4.200431

H 2.426188 -2.038734 2.244601

H 0.367395 -0.960238 2.922128

H 2.901909 -4.935789 1.637033

H 0.302086 1.222242 4.978076

C 3.354102 -2.512581 2.579527

C -0.103050 -1.144334 3.892656

H 6.021182 -4.296182 1.241727

H -2.597286 0.200921 5.904920

H 6.031792 -3.047547 2.501946

H -2.226861 -1.503959 5.582067

C 5.406784 -3.789210 1.989523

C -1.816386 -0.486449 5.571606

H 3.097441 -3.294464 3.309239

H 0.675598 -1.065523 4.664255

H 3.949086 -1.759716 3.106895

H -0.473294 -2.175816 3.916263

H 5.120737 -4.539353 2.741488

H -1.015483 -0.459950 6.324912

H -3.900497 1.886763 -1.006499

C -7.906685 3.071051 -2.051204

C -7.779752 4.458782 -2.089378

C -6.541704 5.030209 -1.803892

C -5.462292 4.196431 -1.483128

N -5.591551 2.853667 -1.446798

C -6.786321 2.296657 -1.728104

H -8.856458 2.589132 -2.271282

H -8.633749 5.085396 -2.340133

H -6.404631 6.108806 -1.826870

C -4.105149 4.767351 -1.154652

H -3.323172 4.292667 -1.759823

H -4.077702 5.848901 -1.326331

H -3.848932 4.580264 -0.102950

C -6.862190 0.790078 -1.695686

H -6.247739 0.357384 -2.497994

H -6.484971 0.399032 -0.741968

H -7.890600 0.437316 -1.831665

--------------------------------------------------------

**Cartesian coordinate of A-PNP.** Units are presented in Å.

SCF energy = -3962.12947960 hartree

ZPE = 1.296398 hartree

SCF energy (in toluene) = -3963.00677881 hartree

-------------------------------------------------------

Atom Coordinates (Angstroms)

X Y Z

--------------------------------------------------------

Mo -2.586570 0.025975 -0.015056

Mo 2.612099 0.054475 0.092727

P 3.082495 -1.999242 1.558963

P -2.991640 2.042931 1.496187

N -4.854182 0.127200 0.067272

N 4.809155 -0.267578 -0.270449

P 3.173740 1.975383 -1.501967

P -3.296347 -1.969803 -1.491128

N -0.607224 0.014971 -0.023535

N 0.554651 0.054763 0.038190

N -2.532795 -1.875292 2.511221

N -2.559155 -1.185811 1.609254

N -2.593308 1.207941 -1.662392

N -2.599235 1.866436 -2.587707

N 3.234193 1.789600 2.537198

N 2.887799 1.104856 1.571517

N 2.321378 -1.212906 -1.681183

N 2.119920 -1.847521 -2.581391

H 2.908773 -2.900352 5.333866

H -2.165870 3.017855 5.172314

H -3.652985 2.282750 4.574223

H 4.289501 -2.237764 4.459041

C 3.384905 -2.847820 4.344003

C -2.814944 2.930551 4.288543

H -2.266780 0.273263 3.884134

H 2.906558 -0.177956 4.017717

H -3.209763 3.926679 4.078214

H 3.686676 -3.862328 4.075668

H -0.847192 1.117289 4.504964

H 1.562393 -0.935884 4.885818

C 2.025930 -0.816849 3.895779

C -1.462492 0.962493 3.606406

H 0.609620 -3.006046 4.305104

H -0.108805 3.221425 3.727198

C 2.383373 -2.214067 3.354131

C -1.988909 2.329378 3.130303

H -4.384718 0.676807 2.913672

H 4.793534 -0.679728 2.617479

H 1.311840 -0.302571 3.245273

H -0.842781 0.475834 2.847342

H 5.478021 -2.283980 2.331260

H -5.221594 2.208941 2.668523

C 1.078819 -3.036912 3.311712

C -0.760830 3.218043 2.840170

H 2.973125 -5.095391 2.494340

H -2.987609 5.162306 2.393932

H 0.355068 -2.622196 2.599168

H -0.175365 2.835111 1.993492

C 4.868989 -1.491936 1.878970

C -4.643638 1.430573 2.155451

H 7.491467 -1.690663 1.219068

H -7.343404 1.394650 1.980829

H 1.243948 -4.090745 3.067241

H -1.024841 4.260630 2.635697

H 4.663338 -4.636000 2.187354

H -4.652338 4.535142 2.395206

H -4.624125 -2.558815 1.112607

H 4.951792 2.397425 0.861277

C 3.703085 -4.871102 1.711427

C -3.819349 4.850413 1.754165

H -3.004976 -5.170365 0.593777

H 3.485917 5.096830 0.674161

C 6.926260 -1.146070 0.467033

C -6.880815 0.866293 1.150687

H 3.848110 -5.794103 1.131166

H -4.155465 5.743552 1.207259

C 5.554008 -0.955041 0.646283

C -5.488001 0.785183 1.083472

H -5.163299 -4.233259 0.946871

H 5.612197 4.013772 0.598149

H -2.172126 -3.616595 0.744489

H 2.573347 3.598109 0.908732

C -4.940847 -3.312660 0.388397

C 5.213096 3.124570 0.089388

C -2.654016 -4.291836 0.032616

C 2.979027 4.272805 0.151085

H -8.744626 0.332705 0.192935

H 8.618934 -0.791507 -0.827719

C 3.235698 -3.756751 0.752079

C -3.417210 3.770252 0.729330

H -5.878702 -2.978729 -0.070312

H 6.024013 2.717711 -0.526842

C -7.658981 0.275619 0.157796

C 7.552814 -0.642350 -0.670604

H -1.896716 -4.639554 -0.676283

H 2.144937 4.704475 -0.410038

H 1.112726 -4.329494 0.934435

H -1.344052 4.508670 0.553481

H 5.298000 -3.572701 0.001017

H -5.542438 3.361137 0.305638

C -3.861871 -3.633820 -0.672428

C 3.999704 3.559707 -0.764097

C 1.862910 -4.146634 0.159181

C -2.204559 4.277437 -0.081730

C 4.279777 -3.694226 -0.385963

C -4.615065 3.591235 -0.231787

H 4.254061 -4.645219 -0.936933

H -4.776269 4.538398 -0.766147

H -4.879297 -5.496933 -1.068533

H 5.063904 5.359185 -1.299569

C 6.791477 0.065095 -1.597327

C -7.012728 -0.389370 -0.881440

H 1.465554 -3.377692 -0.510093

H -1.886126 3.557103 -0.839210

C 5.425352 0.255156 -1.371505

C -5.617281 -0.455069 -0.904332

C -4.485566 -4.650402 -1.649816

C 4.514055 4.560180 -1.817530

H -5.327003 -4.231030 -2.215822

H 5.210702 4.102802 -2.531606

H 1.970395 -5.074901 -0.420669

H -2.486048 5.204842 -0.601818

H -7.580715 -0.862414 -1.678821

H 7.248657 0.486093 -2.489184

H 4.095021 -2.894355 -1.106373

H -4.450742 2.815449 -0.983327

H -1.760754 -4.433902 -2.789437

H 1.573248 4.553755 -2.452124

C -4.915632 -1.176145 -2.029556

C 4.606073 1.071971 -2.341184

H -3.760291 -5.059868 -2.359518

H 3.705532 5.038607 -2.378236

H -5.605080 -1.883562 -2.506643

H 5.264055 1.741882 -2.907655

H -0.667265 -3.160727 -2.201606

H 0.549908 3.314166 -1.688409

C -1.362287 -3.437426 -3.005043

C 1.091330 3.582496 -2.604333

H -4.630009 -0.445918 -2.800996

H 4.131440 0.408313 -3.078604

C -2.466293 -2.377447 -3.196946

C 2.087593 2.482900 -3.025866

H -1.076268 -0.693089 -2.975401

H 0.666551 0.857986 -2.612968

H -0.778655 -3.515581 -3.933448

H 0.344156 3.712166 -3.399996

C -1.788302 -1.090980 -3.704758

C 1.265864 1.247366 -3.441522

H -3.975987 -3.770329 -4.037954

H 3.491994 3.838660 -4.082712

H -4.209177 -2.076426 -4.510575

H 3.554056 2.155786 -4.640064

C -3.456552 -2.842093 -4.286398

C 2.891472 2.943850 -4.260727

H -1.241182 -1.322103 -4.630430

H 0.576804 1.535509 -4.247753

H -2.508493 -0.301409 -3.943320

H 1.895394 0.439966 -3.833463

H -2.896163 -3.019581 -5.216051

H 2.184033 3.180358 -5.068831

H 2.419827 2.153717 3.071305

--------------------------------------------------------

**Cartesian coordinate of transition state for N_2_ elimination from A-PNP (A-PNP → B-PNP).**  Units are presented in Å.

SCF energy = -3962.11795020 hartree

ZPE = 1.294489 hartree

SCF energy (in toluene) = -3962.99789746 hartree

Imaginary frequency: 74i cm^-1^

-------------------------------------------------------

Atom Coordinates (Angstroms)

X Y Z

--------------------------------------------------------

Mo -2.528343 0.039534 -0.038439

Mo 2.604905 0.092278 0.190092

P 3.021371 -1.993633 1.602864

P -2.863030 2.177621 1.346275

N -4.794557 0.180117 0.126383

N 4.777018 -0.334023 -0.243335

P 3.166681 1.951974 -1.453329

P -3.328443 -2.035556 -1.347578

N -0.569157 -0.033274 -0.102959

N 0.603015 -0.056913 -0.082103

N -2.461498 -1.642527 2.637359

N -2.484083 -1.036244 1.678073

N -2.574304 1.112128 -1.757244

N -2.606540 1.714826 -2.718981

N 2.666417 1.952132 2.576820

N 2.776082 1.167922 1.613054

N 2.309613 -1.651693 -2.382640

N 1.925863 -2.280171 -3.211068

H 2.933807 -3.045266 5.340084

H -1.873138 3.403547 4.893496

H -3.347084 2.546513 4.442505

H 4.335812 -2.479836 4.432414

C 3.371200 -2.993372 4.332488

C -2.568681 3.219301 4.061852

H -1.923152 0.571744 3.774005

H 3.153904 -0.253356 4.073395

H -3.034948 4.175087 3.811214

H 3.560373 -4.018951 4.008872

H -0.497923 1.503616 4.250540

H 1.838301 -0.918806 5.048087

C 2.213419 -0.813824 4.019487

C -1.164466 1.271682 3.408124

H 0.591488 -2.870279 4.421808

H 0.098699 3.577925 3.321312

C 2.396047 -2.219809 3.419055

C -1.776526 2.589693 2.895298

H -4.178811 0.888290 2.902810

H 4.866331 -0.867000 2.656784

H 1.499696 -0.210150 3.454775

H -0.568446 0.770812 2.638976

H 5.421551 -2.489117 2.245372

H -5.028858 2.406458 2.621412

C 1.014473 -2.906203 3.407784

C -0.612912 3.515635 2.487465

H 2.659027 -5.109698 2.415459

H -2.898882 5.338641 2.032286

H 0.307683 -2.393200 2.744333

H -0.055820 3.120446 1.629802

C 4.852115 -1.631546 1.865771

C -4.478285 1.600867 2.120358

H 7.442644 -1.897099 1.139133

H -7.186375 1.569623 2.076940

H 1.062156 -3.960729 3.116901

H -0.944476 4.533245 2.256864

H 4.379767 -4.816310 2.074537

H -4.548213 4.680858 2.130334

H -4.572571 -2.444006 1.328602

H 4.907650 2.300852 0.900976

C 3.386822 -4.924446 1.620333

C -3.744495 4.972350 1.442222

H -2.993323 -5.092799 0.932179

H 3.548800 5.102709 0.674367

C 6.880996 -1.311307 0.415804

C -6.764814 0.991844 1.257998

H 3.417111 -5.828352 0.993931

H -4.118902 5.822255 0.852887

C 5.521883 -1.074673 0.634787

C -5.376669 0.899889 1.130696

H -5.137458 -4.117354 1.284268

H 5.613566 3.917970 0.699751

H -2.154253 -3.534945 0.958591

H 2.582987 3.639479 0.937280

C -4.921879 -3.236403 0.662789

C 5.210733 3.048137 0.161406

C -2.655382 -4.254350 0.305584

C 3.022052 4.280204 0.167187

H -8.672743 0.416117 0.419288

H 8.555939 -0.983825 -0.909777

C 3.015290 -3.722322 0.728831

C -3.356207 3.837017 0.473002

H -5.869510 -2.918390 0.213394

H 6.031860 2.629849 -0.433621

C -7.590114 0.350179 0.337562

C 7.499689 -0.801809 -0.723473

H -1.917350 -4.649931 -0.398532

H 2.211258 4.727702 -0.414795

H 0.854488 -4.101914 0.932203

H -1.300044 4.577780 0.159239

H 5.075193 -3.705216 -0.046556

H -5.492498 3.386314 0.161754

C -3.879671 -3.636943 -0.406535

C 4.032476 3.519789 -0.721539

C 1.601417 -3.944503 0.147410

C -2.189361 4.312510 -0.419671

C 4.045193 -3.695758 -0.422352

C -4.586113 3.572034 -0.425965

H 3.915473 -4.598232 -1.036942

H -4.772664 4.469453 -1.033282

H -4.915964 -5.517173 -0.649189

H 5.184681 5.265651 -1.258560

C 6.739584 -0.051258 -1.616954

C -6.995456 -0.378399 -0.690281

H 1.266675 -3.092897 -0.454463

H -1.906458 3.559062 -1.158989

C 5.386599 0.179873 -1.355051

C -5.603157 -0.455009 -0.772046

C -4.541397 -4.710024 -1.295777

C 4.603062 4.488218 -1.775028

H -5.403912 -4.321604 -1.852348

H 5.281789 3.997550 -2.484429

H 1.603561 -4.839049 -0.492764

H -2.505332 5.212301 -0.968001

H -7.601454 -0.893520 -1.431647

H 7.188011 0.368428 -2.513905

H 3.936855 -2.829350 -1.079706

H -4.444238 2.734666 -1.113286

H -1.882840 -4.598927 -2.534192

H 1.595559 4.551423 -2.414047

C -4.953048 -1.249484 -1.878875

C 4.572236 1.019533 -2.306975

H -3.847494 -5.168194 -2.007760

H 3.818406 4.999674 -2.341138

H -5.668233 -1.972060 -2.290918

H 5.236644 1.673710 -2.884512

H -0.742984 -3.322604 -2.055325

H 0.571291 3.322810 -1.633870

C -1.466987 -3.628314 -2.821567

C 1.104773 3.582821 -2.556447

H -4.682602 -0.568463 -2.699351

H 4.073809 0.364888 -3.036955

C -2.551831 -2.554977 -3.046055

C 2.091855 2.477104 -2.980186

H -1.112267 -0.892822 -2.973009

H 0.678004 0.839981 -2.570178

H -0.914589 -3.776663 -3.760352

H 0.351800 3.714207 -3.346521

C -1.860775 -1.316826 -3.650533

C 1.266222 1.245046 -3.399379

H -4.111356 -3.964027 -3.759548

H 3.500060 3.830220 -4.033030

H -4.327393 -2.296036 -4.325483

H 3.558578 2.149092 -4.595480

C -3.582746 -3.062150 -4.077548

C 2.897338 2.937402 -4.213965

H -1.354389 -1.616185 -4.579976

H 0.568873 1.537868 -4.197060

H -2.570686 -0.524979 -3.910342

H 1.899450 0.449275 -3.807734

H -3.054089 -3.306125 -5.010709

H 2.191290 3.178125 -5.022141

H 3.596427 2.266578 2.908277

--------------------------------------------------------

**Cartesian coordinate of product complex for N_2_ elimination from A-PNP (A-PNP → B-PNP).** Units are presented in Å.

SCF energy = -3962.12551840 hartree

ZPE = 1.293761 hartree

SCF energy (in toluene) = -3963.00719543 hartree

-------------------------------------------------------

Atom Coordinates (Angstroms)

X Y Z

--------------------------------------------------------

Mo -2.523386 0.006767 -0.080014

Mo 2.581632 0.321163 0.376530

P 2.906542 -1.505041 2.058597

P -2.992835 2.291351 0.997937

N -4.805699 0.074312 0.011453

N 4.792149 -0.155220 0.102366

P 3.118248 1.700163 -1.656824

P -3.217393 -2.256120 -1.132953

N -0.560394 0.026091 -0.051307

N 0.610935 0.068420 0.043953

N -2.519082 -1.321150 2.788897

N -2.513389 -0.842753 1.759586

N -2.549022 0.849895 -1.925043

N -2.581785 1.320243 -2.957515

N 2.547386 2.592005 2.374254

N 2.716919 1.651527 1.569772

N 4.512320 -2.759281 -3.631619

N 4.332977 -3.838771 -3.801964

H 2.737817 -2.193971 5.873931

H -2.121375 3.993455 4.372535

H -3.549831 3.020494 4.014967

H 4.177744 -1.804402 4.934545

C 3.182514 -2.261145 4.870418

C -2.791720 3.671946 3.562332

H -2.038722 1.028649 3.611332

H 3.136599 0.461447 4.317797

H -3.291435 4.565601 3.180793

H 3.306719 -3.323002 4.641598

H -0.671229 2.077207 4.006667

H 1.788069 -0.014267 5.355452

C 2.163836 -0.046102 4.322181

C -1.300818 1.714599 3.182069

H 0.424477 -1.968581 4.931069

H -0.103757 4.015506 2.811224

C 2.260343 -1.520171 3.878209

C -1.951801 2.932564 2.498715

H -4.293210 1.167730 2.684450

H 4.777702 -0.421809 3.091073

H 1.485728 0.534543 3.691681

H -0.660321 1.155237 2.493104

H 5.259633 -2.086741 2.785408

H -5.195932 2.596993 2.183166

C 0.846071 -2.128496 3.928572

C -0.815100 3.843728 1.992752

H 2.502598 -4.537236 3.087255

H -3.172836 5.504000 1.284150

H 0.166570 -1.654076 3.211338

H -0.247059 3.375947 1.179900

C 4.743779 -1.225869 2.341232

C -4.601388 1.755416 1.807472

H 7.381223 -1.441806 1.865481

H -7.302175 1.600185 1.713476

H 0.846234 -3.209374 3.748405

H -1.175894 4.821353 1.656065

H 4.210196 -4.289314 2.658597

H -4.799658 4.797157 1.412411

H -4.538913 -2.353490 1.522431

H 4.671651 2.961873 0.476730

C 3.199263 -4.419550 2.250981

C -3.983116 5.034123 0.718431

H -2.822489 -4.995233 1.511571

H 2.815918 5.321948 -0.520429

C 6.867473 -0.957510 1.038802

C -6.837022 0.938896 0.986555

H 3.195665 -5.367182 1.692285

H -4.367146 5.788965 0.016741

C 5.484928 -0.766301 1.111578

C -5.443456 0.891708 0.900535

H -5.002593 -4.047000 1.719894

H 5.117513 4.552142 -0.171891

H -2.069640 -3.397679 1.397051

H 2.221288 3.833933 0.241823

C -4.823508 -3.255999 0.977538

C 4.873375 3.515688 -0.445803

C -2.506427 -4.209621 0.809586

C 2.497260 4.287063 -0.715860

H -8.697883 0.173793 0.194275

H 8.647921 -0.656405 -0.148240

C 2.794967 -3.275764 1.301956

C -3.517184 3.805673 -0.089390

H -5.777054 -3.067017 0.470941

H 5.766384 3.087885 -0.918952

C -7.611741 0.145759 0.143386

C 7.570514 -0.521283 -0.080729

H -1.720779 -4.634158 0.177276

H 1.604098 4.335809 -1.348450

H 0.620448 -3.518164 1.619529

H -1.494035 4.621536 -0.434904

H 4.784722 -3.230068 0.333439

H -5.619095 3.215582 -0.407536

C -3.733525 -3.743611 -0.004783

C 3.656716 3.528812 -1.398855

C 1.350190 -3.497462 0.804709

C -2.339376 4.219288 -1.000186

C 3.724945 -3.329685 0.069703

C -4.701002 3.374039 -0.985145

H 3.600320 -4.303348 -0.425849

H -4.905959 4.180516 -1.703745

H -4.694020 -5.677874 -0.026553

H 4.477311 5.251925 -2.412596

C 6.862786 0.083611 -1.115684

C -6.960968 -0.684370 -0.766103

H 1.042163 -2.712599 0.102281

H -1.978291 3.387733 -1.610872

C 5.479274 0.247373 -1.009580

C -5.565039 -0.708032 -0.810153

C -4.321982 -4.950707 -0.762950

C 4.067000 4.268905 -2.685989

H -5.172166 -4.672242 -1.398106

H 4.845399 3.739821 -3.250861

H 1.292517 -4.464498 0.283358

H -2.681698 5.008465 -1.685690

H -7.525368 -1.319631 -1.444408

H 7.371113 0.425089 -2.013807

H 3.482415 -2.553992 -0.668372

H -4.493360 2.467895 -1.558778

H -1.611735 -4.865277 -1.941719

H 1.108821 3.469522 -3.303549

C -4.857933 -1.611900 -1.788869

C 4.711391 0.830037 -2.165648

H -3.583126 -5.470429 -1.380939

H 3.216825 4.449437 -3.352045

H -5.532155 -2.415009 -2.109744

H 5.368273 1.463352 -2.774695

H -0.547206 -3.468009 -1.668714

H 0.312035 2.279441 -2.247318

C -1.253989 -3.925129 -2.373223

C 0.849617 2.410134 -3.194248

H -4.595534 -1.037150 -2.688839

H 4.400267 0.000645 -2.816303

C -2.399881 -2.962049 -2.744303

C 2.090387 1.499801 -3.274201

H -1.063502 -1.226280 -2.901194

H 1.008143 -0.218911 -2.427162

H -0.695382 -4.179799 -3.285435

H 0.153658 2.157121 -4.006292

C -1.781974 -1.779061 -3.513385

C 1.615701 0.030058 -3.302769

H -3.859986 -4.553327 -3.269391

H 3.195147 2.810056 -4.678623

H -4.179519 -2.990585 -4.045306

H 3.730873 1.119346 -4.703940

C -3.388581 -3.666889 -3.699216

C 2.859050 1.774585 -4.584159

H -1.253441 -2.170847 -4.395082

H 0.998736 -0.128412 -4.198775

H -2.535885 -1.072670 -3.876510

H 2.449901 -0.683270 -3.360493

H -2.836339 -3.992843 -4.592802

H 2.189067 1.571852 -5.432442

H 3.456793 2.968592 2.696107

--------------------------------------------------------

**Cartesian coordinate of B-PNP.** Units are presented in Å.

SCF energy = -3852.66433849 hartree

ZPE = 1.286697 hartree

SCF energy (in toluene) = -3853.50844334 hartree

-------------------------------------------------------

Atom Coordinates (Angstroms)

X Y Z

--------------------------------------------------------

Mo -2.433642 -0.044751 0.057572

Mo 2.691054 -0.066141 -0.175717

P 3.037224 2.066921 -1.445724

P -2.728343 -2.136666 -1.406677

N -4.698894 -0.196193 -0.156980

N 4.891729 0.368772 0.240131

P 3.207367 -1.808909 1.564599

P -3.279810 1.981181 1.422590

N -0.475915 0.033627 0.144415

N 0.699028 0.064788 0.115142

N -2.380158 1.722899 -2.561724

N -2.387423 1.087737 -1.621068

N -2.520885 -1.176298 1.737638

N -2.595499 -1.809718 2.676710

N 2.797468 -1.894185 -2.583783

N 2.902914 -1.130109 -1.601350

H 2.904869 3.502554 -5.047206

H -1.647316 -3.205033 -4.975509

H -3.143397 -2.388780 -4.521881

H 4.336132 2.930770 -4.190594

C 3.339883 3.366577 -4.046446

C -2.362770 -3.065135 -4.152228

H -1.749455 -0.424010 -3.752587

H 3.296066 0.583598 -4.028853

H -2.818768 -4.037690 -3.951420

H 3.460600 4.360550 -3.607234

H -0.306993 -1.327291 -4.230563

H 1.962548 1.252250 -4.978330

C 2.322786 1.084097 -3.952895

C -0.993169 -1.130789 -3.395086

H 0.575861 3.079962 -4.188781

H 0.294268 -3.422475 -3.362025

C 2.409951 2.443651 -3.230198

C -1.603446 -2.472497 -2.944995

H -4.030565 -0.811283 -2.945027

H 4.923913 1.193072 -2.640013

H 1.636274 0.393747 -3.455901

H -0.417086 -0.653496 -2.596208

H 5.399527 2.771571 -2.022264

H -4.868059 -2.345349 -2.724146

C 0.994003 3.049628 -3.172603

C -0.439678 -3.401799 -2.546003

H 2.669815 5.245996 -1.838486

H -2.707718 -5.268759 -2.213382

H 0.313341 2.447389 -2.559331

H 0.091011 -3.034961 -1.659807

C 4.878244 1.843875 -1.754233

C -4.334399 -1.550197 -2.189229

H 7.504939 1.979176 -1.189527

H -7.041164 -1.541273 -2.197017

H 0.992477 4.077480 -2.792265

H -0.765301 -4.431061 -2.363810

H 4.366134 4.897831 -1.437589

H -4.363775 -4.629401 -2.319487

H -4.484760 2.477718 -1.262990

H 4.849698 -2.561485 -0.698213

C 3.348431 4.954671 -1.030447

C -3.569424 -4.935636 -1.626831

H -2.907483 5.115698 -0.744058

H 3.015276 -5.140354 -0.263541

C 6.978772 1.344183 -0.481123

C -6.640723 -0.985862 -1.352433

H 3.340001 5.769172 -0.291155

H -3.943215 -5.812029 -1.077147

C 5.599883 1.162943 -0.619562

C -5.256114 -0.888341 -1.194333

H -5.047467 4.149097 -1.169824

H 5.293567 -4.258460 -0.418658

H -2.073413 3.556796 -0.821254

H 2.421929 -3.540107 -0.748255

C -4.842714 3.247239 -0.575388

C 5.035190 -3.311992 0.077458

C -2.581186 4.251440 -0.147000

C 2.672614 -4.165530 0.114565

H -8.569469 -0.447515 -0.536718

H 8.736598 0.833991 0.667726

C 2.916967 3.646268 -0.342571

C -3.215905 -3.832785 -0.607582

H -5.797610 2.916225 -0.151382

H 5.912969 -3.000260 0.657559

C -7.489227 -0.377338 -0.430730

C 7.662223 0.708305 0.551595

H -1.851867 4.615304 0.583023

H 1.759654 -4.342598 0.693991

H 0.755831 3.989808 -0.648960

H -1.163086 -4.576724 -0.281956

H 4.884551 3.388083 0.636603

H -5.362912 -3.415310 -0.328434

C -3.817266 3.611258 0.523123

C 3.795496 -3.540353 0.970909

C 1.462191 3.774925 0.158290

C -2.060927 -4.327709 0.290816

C 3.820120 3.438367 0.894156

C -4.468297 -3.615047 0.272714

H 3.688145 4.290656 1.576188

H -4.658747 -4.537203 0.840220

H -4.860549 5.480852 0.807457

H 4.621970 -5.418758 1.646986

C 6.938291 -0.084485 1.438009

C -6.920311 0.324132 0.629816

H 1.127379 2.857330 0.658459

H -1.788430 -3.592230 1.052079

C 5.559044 -0.231784 1.271342

C -5.530534 0.406083 0.743007

C -4.492685 4.656027 1.435200

C 4.184618 -4.515642 2.097198

H -5.360995 4.250409 1.969869

H 4.936136 -4.099696 2.780736

H 1.400856 4.600519 0.882641

H -2.383145 -5.241125 0.812066

H -7.544385 0.813568 1.373594

H 7.430641 -0.586618 2.266921

H 3.562375 2.529502 1.454356

H -4.351339 -2.803653 0.994961

H -1.840831 4.490002 2.711872

H 1.183634 -3.885406 2.777176

C -4.911195 1.169574 1.888351

C 4.770197 -1.027958 2.275959

H -3.807787 5.094392 2.168007

H 3.319695 -4.837567 2.687369

H -5.639760 1.876642 2.303461

H 5.420351 -1.757046 2.774598

H -0.696725 3.208814 2.251680

H 0.390430 -2.516830 1.961099

C -1.446142 3.509126 2.994770

C 0.912172 -2.827771 2.874254

H -4.658388 0.465791 2.695061

H 4.425696 -0.341261 3.062881

C -2.547748 2.442389 3.157077

C 2.137764 -1.938875 3.162417

H -1.115552 0.776709 3.075371

H 1.068989 -0.092772 2.625935

H -0.928664 3.633141 3.957105

H 0.196919 -2.749526 3.705179

C -1.884017 1.180842 3.741765

C 1.640519 -0.507327 3.462415

H -4.114110 3.843425 3.872875

H 3.208082 -3.494769 4.315140

H -4.358441 2.161649 4.382987

H 3.751366 -1.843993 4.669691

C -3.601341 2.927744 4.175970

C 2.878484 -2.457664 4.412721

H -1.405904 1.447500 4.695900

H 0.982591 -0.533871 4.342706

H -2.605095 0.384920 3.953755

H 2.460915 0.185392 3.699126

H -3.094383 3.138525 5.129018

H 2.193279 -2.412940 5.271738

H 3.730617 -2.181261 -2.928894

--------------------------------------------------------

**Cartesian coordinate of C-PNP.** Units are presented in Å.

SCF energy = -4813.91148709 hartree

ZPE = 1.315764 hartree

SCF energy (in toluene) = -4814.96116730 hartree

-------------------------------------------------------

Atom Coordinates (Angstroms)

X Y Z

--------------------------------------------------------

Mo -2.878837 -0.005686 0.037107

Mo 2.308314 0.529290 0.384036

P 2.896258 -0.820682 2.510119

P -3.509272 2.427834 0.536749

N -5.135027 -0.062691 0.038145

N 4.518637 0.796370 0.485808

P 2.739538 2.014657 -1.635401

P -3.362333 -2.455665 -0.486229

N -0.867061 0.044199 0.075738

N 0.288609 0.091956 0.123283

N -2.893777 -0.583495 3.146985

N -2.881829 -0.373800 2.029318

N -2.870768 0.381185 -1.952333

N -2.882367 0.611701 -3.065615

N 1.816178 2.958394 2.150306

N 2.045849 1.957448 1.430471

H 1.865449 -0.559582 6.250342

H -2.973481 4.944413 3.461575

H -4.327546 3.832993 3.244712

H 3.208163 0.260141 5.448767

C 2.579016 -0.638920 5.416261

C -3.570430 4.414884 2.703945

H -2.651961 1.952754 3.441068

H 1.482690 1.459283 3.985270

H -4.083281 5.173187 2.106550

H 3.213157 -1.507157 5.611263

H -1.329223 3.124104 3.568414

H 0.228992 0.556006 4.858053

C 0.907556 0.529103 3.992275

C -1.936136 2.543980 2.859256

H 0.081393 -1.784970 4.909105

H -0.887503 4.795483 2.005283

C 1.786719 -0.729357 4.094959

C -2.625647 3.512665 1.882142

H -4.786856 1.616962 2.403020

H 3.865902 1.243356 3.295768

H 0.298774 0.517749 3.084521

H -1.265706 1.859596 2.334313

H 5.000186 -0.049017 3.704194

H -5.778897 2.833658 1.595844

C 0.828958 -1.940604 4.117673

C -1.523320 4.362127 1.221282

H 3.250936 -3.368410 4.496254

H -3.935450 5.603168 0.039061

H 0.282756 -2.037892 3.170628

H -0.868987 3.752518 0.587684

C 4.342443 0.329246 2.911928

C -5.114483 1.978784 1.417374

H 7.025143 0.753232 2.753509

H -7.795724 1.618334 1.292445

H 1.330407 -2.890749 4.324321

H -1.925577 5.188619 0.625063

H 4.799787 -2.498842 4.413338

H -5.511646 4.819426 0.295283

H -4.799007 -2.024475 2.065979

H 3.929183 3.511258 0.630404

C 4.112294 -3.150602 3.858161

C -4.685741 4.949174 -0.416184

H -2.940763 -4.463222 2.746921

H 1.963428 5.654216 -0.681206

C 6.540436 0.858765 1.785958

C -7.260788 0.848548 0.740870

H 4.639486 -4.101815 3.689935

H -5.093067 5.483151 -1.288240

C 5.160347 0.670226 1.685608

C -5.864908 0.881126 0.708689

H -5.181278 -3.650274 2.647791

H 4.205804 5.143399 -0.006107

H -2.273281 -2.896176 2.263369

H 1.448815 4.157433 0.119152

C -5.010436 -3.046176 1.744321

C 4.145888 4.078760 -0.277639

C -2.638025 -3.853411 1.882035

C 1.764679 4.582249 -0.838734

H -9.037190 -0.193879 0.076527

H 8.361096 1.283319 0.702509

C 3.733325 -2.565547 2.482217

C -4.099960 3.604442 -0.891116

H -5.946732 -3.040560 1.173999

H 5.136367 3.780376 -0.639501

C -7.949926 -0.157192 0.065760

C 7.281937 1.155302 0.643140

H -1.802475 -4.365292 1.395445

H 0.934233 4.506915 -1.546070

H 1.866178 -3.727011 2.334178

H -2.123308 4.464197 -1.367814

H 5.813992 -1.903458 2.239753

H -6.133397 2.781160 -1.105397

C -3.850872 -3.677232 0.941283

C 3.053319 3.908502 -1.357648

C 2.775034 -3.531630 1.754164

C -2.916668 3.880789 -1.844388

C 5.046798 -2.454837 1.681930

C -5.211808 2.886845 -1.689332

H 5.435188 -3.466738 1.501569

H -5.453662 3.493238 -2.574947

H -4.691637 -5.624940 1.354847

H 3.817269 5.683045 -2.328199

C 6.619015 1.294642 -0.572596

C -7.208213 -1.114920 -0.623199

H 2.482237 -3.139567 0.777814

H -2.476741 2.957373 -2.229395

C 5.229801 1.135603 -0.629515

C -5.812996 -1.054010 -0.618442

C -4.333341 -5.066154 0.477008

C 3.557062 4.651998 -2.612430

H -5.171365 -5.005463 -0.229775

H 4.462139 4.195875 -3.033762

H 3.281567 -4.494410 1.593170

H -3.280033 4.460059 -2.706846

H -7.701157 -1.919343 -1.164337

H 7.162858 1.541143 -1.481088

H 4.912020 -1.999243 0.701541

H -4.911287 1.897328 -2.039865

H -1.595027 -5.080455 -0.597495

H 0.992663 3.820565 -3.604913

C -5.005740 -2.099609 -1.341960

C 4.490004 1.368931 -1.925036

H -3.538074 -5.662697 0.019500

H 2.806176 4.716493 -3.405238

H -5.618602 -2.992723 -1.516590

H 5.089778 2.013544 -2.580617

H -0.620003 -3.591489 -0.620504

H -0.003464 2.700449 -2.646320

C -1.262789 -4.244730 -1.222744

C 0.687594 2.774850 -3.495267

H -4.714794 -1.713438 -2.329838

H 4.360583 0.412518 -2.451932

C -2.435280 -3.470100 -1.855663

C 1.870735 1.794379 -3.357009

H -1.190557 -1.733358 -2.371714

H 0.609225 0.185668 -2.551644

H -0.635802 -4.663560 -2.020270

H 0.118681 2.506827 -4.397365

C -1.845516 -2.461126 -2.859496

C 1.279797 0.371614 -3.396684

H -3.784423 -5.223596 -2.036571

H 3.300283 2.931794 -4.615915

H -4.141280 -3.913804 -3.179447

H 3.618698 1.182045 -4.535091

C -3.336030 -4.439283 -2.650986

C 2.831455 1.944567 -4.554909

H -1.246838 -3.008816 -3.599733

H 0.695393 0.263178 -4.323075

H -2.621456 -1.914609 -3.406145

H 2.058667 -0.391238 -3.405143

H -2.721626 -4.934699 -3.417462

H 2.261284 1.794095 -5.484022

H 2.691285 3.464308 2.378461

S 3.631022 -2.216384 -1.925565

O 3.984824 -1.503533 -3.175307

O 4.687026 -3.041899 -1.320103

O 2.856130 -1.367525 -0.940401

C 2.344518 -3.454536 -2.462899

F 1.945589 -4.207936 -1.421043

F 1.262596 -2.839438 -2.974139

F 2.855550 -4.262884 -3.399357

--------------------------------------------------------
